# Clinical benefits of modifying the evening light environment in an acute psychiatric unit: A single-centre, two-arm, parallel-group, pragmatic effectiveness randomised controlled trial

**DOI:** 10.1371/journal.pmed.1004380

**Published:** 2024-12-06

**Authors:** Håvard Kallestad, Knut Langsrud, Melanie Rae Simpson, Cecilie Lund Vestergaard, Daniel Vethe, Kaia Kjørstad, Patrick Faaland, Stian Lydersen, Gunnar Morken, Ingvild Ulsaker-Janke, Simen Berg Saksvik, Jan Scott

**Affiliations:** 1 Division of Mental Health Care, St. Olavs hospital, Trondheim University Hospital, Trondheim, Norway; 2 Department of Mental Health, Norwegian University of Science and Technology, Trondheim, Norway; 3 Department of Public Health and Nursing, Norwegian University of Science and Technology, Trondheim, Norway; 4 Department of Health Promotion, Norwegian Institute of Public Health, Bergen, Norway; 5 Institute of Neuroscience, Newcastle University, Newcastle, United Kingdom; Massachusetts General Hospital, UNITED STATES OF AMERICA

## Abstract

**Background:**

The impact of light exposure on mental health is increasingly recognised. Modifying inpatient evening light exposure may be a low-intensity intervention for mental disorders, but few randomised controlled trials (RCTs) exist. We report a large-scale pragmatic effectiveness RCT exploring whether individuals with acute psychiatric illnesses experience additional benefits from admission to an inpatient ward where changes in the evening light exposure are integrated into the therapeutic environment.

**Methods and findings:**

From 10/25/2018 to 03/29/2019, and 10/01/2019 to 11/15/2019, all adults (≥18 years of age) admitted for acute inpatient psychiatric care in Trondheim, Norway, were randomly allocated to a ward with a blue-depleted evening light environment or a ward with a standard light environment. Baseline and outcome data for individuals who provided deferred informed consent were used. The primary outcome measure was the mean duration of admission in days per individual. Secondary outcomes were estimated mean differences in key clinical outcomes: Improvement during admission (The Clinical Global Impressions Scale–Improvement, CGI-I) and illness severity at discharge (CGI-S), aggressive behaviour during admission (Broset Violence Checklist, BVC), violent incidents (Staff Observation Aggression Scale-Revised, SOAS-R), side effects and patient satisfaction, probabilities of suicidality, need for supervision due to suicidality, and change from involuntary to voluntary admission. The Intent to Treat sample comprised 476 individuals (mean age 37 (standard deviation (SD) 13.3); 193 (41%) were male, 283 (59%) were female). There were no differences in the mean duration of admission (7.1 days for inpatients exposed to the blue-depleted evening light environment versus 6.7 days for patients exposed to the standard evening light environment; estimated mean difference: 0.4 days (95% confidence interval (CI) [−0.9, 1.9]; *p* = 0.523). Inpatients exposed to the blue-depleted evening light showed higher improvement during admission (CGI-I difference 0.28 (95% CI [0.02, 0.54]; *p* = 0.035), Number Needed to Treat for clinically meaningful improvement (NNT): 12); lower illness severity at discharge (CGI-S difference −0.18 (95% CI [−0.34, −0.02]; *p* = 0.029), NNT for mild severity at discharge: 7); and lower levels of aggressive behaviour (difference in BVC predicted serious events per 100 days: −2.98 (95% CI [−4.98, −0.99]; *p* = 0.003), NNT: 9). There were no differences in other secondary outcomes. The nature of this study meant it was impossible to blind patients or clinical staff to the lighting condition.

**Conclusions:**

Modifying the evening light environment in acute psychiatric hospitals according to chronobiological principles does not change duration of admissions but can have clinically significant benefits without increasing side effects, reducing patient satisfaction or requiring additional clinical staff.

**Trial registration:**

Clinicaltrials.gov NCT03788993; 2018 (CRISTIN ID 602154).

## Abstract 2

Håvard Kallestad and colleagues report that modifying the evening light environment may improve clinical outcomes for patients admitted to acute psychiatric inpatient units.

## Author summary

**Why was this study done?:**

The quality and fabric of many psychiatric inpatient facilities have improved over recent decades, but little attention has been given to how to use the environment of the unit to further optimise treatment and improve the mental state of patients.

Evening exposure to light at specific frequencies in the blue spectrum may have deleterious effects on sleep, circadian rhythms, and mental state.

Reducing evening exposure to blue light may improve clinical outcomes for patients with mental disorders, but few studies have been performed.

**What did the researchers do and find?:**

We built a new acute psychiatric unit with 2 wards, where each hospital ward had the same staffing levels, layout, and facilities, but had different evening light environments: standard light and blue-depleted light. We then randomly allocated 476 patients admitted for acute inpatient psychiatric care to the ward with the blue-depleted evening light environment or the ward with the standard light environment.

All patients received standard care, and there were no differences in length of stay, but we observed that patients admitted to the blue-depleted light environment had additional clinical improvement and displayed less aggressive behaviour during admission.

The effect sizes indicated moderate but clinically relevant effects, especially considering the short exposure in this study, where patients had a median length of admission of about 4 days, and the unobtrusiveness of the intervention, which was without adverse events or side effects.

**What does these findings mean?:**

Modifying the evening light environment may improve clinical outcomes for patients admitted to acute psychiatric inpatient units.

This may also be relevant for inpatient units in different populations and specialties.

Future research may address dosage and adherence, as well as finding optimal patient populations.

A limitation with the study was that it was not possible to blind patients or clinical staff to the randomised lighting condition.

## Introduction

Light is the most critical environmental factor for circadian rhythmicity and research over several decades indicates that manipulating light and dark exposure may improve clinical outcomes [1–3]. The study of chronotherapies has been galvanised by research demonstrating that the circadian effect of light on humans is primarily mediated by intrinsically photosensitive retinal ganglion cells (ipRGC) that have peak sensitivity to blue light [4–7]. This discovery indicates that it may be feasible to achieve clinical improvements by specifically blocking the blue part of the light spectrum in the evening, so-called virtual darkness, without recourse to prolonged sensory deprivation which is employed in dark therapies [8]. For example, several studies, including small-scale randomised controlled trials (RCTs), have examined the potential benefits of the adjunctive evening use of “blue blocking glasses” (BBG) compared with standard treatment for individuals with insomnia, delayed sleep phase, major depression, postpartum disorders, bipolar disorder, and mania [1,9–15]. At this stage, it is impossible to determine reliably whether the use of BBG is associated with robust, clinically significant effects in certain clinical subgroups. In many instances, the studies recruited small convenience samples (typically 5 to 20 participants selected by the investigators), some intervention groups included cases with a range of different primary diagnoses or problems, some studies lacked a control or comparator group and/or the analyses were reported only for completers. Even allowing for methodological limitations, the interpretation of the benefits of BBG is hampered by the fact that different researcher groups used different rating scales to measure outcomes, e.g., some focused only on changes in sleep patterns, other focused on mood or social functioning. The studies focused on mood disorders showed some putative benefits, but effect sizes varied widely and those studies reporting the largest effects often recruited smaller or less representative clinical samples. Overall, we suggest that cross-study findings are inconsistent in terms of the magnitude of any benefits of BBG or the specific nature of any benefits (BBG could improve sleep or mood but might influence other acute psychiatric symptoms, such as agitation and aggression or suicidality, across a broad range of mental disorders [1,9–15].

Another issue in optimising the effectiveness of BBG and other individual chronotherapy interventions is that many individuals, especially acutely mentally ill inpatients, struggle to adhere with the interventions or lack capacity to participate in research. One way to reduce reliance on individual behaviour and therefore increase access to chronotherapy for severe mental disorders is to consider changing the environment in which the individual is residing. For example, some new-build general hospitals now employ dynamic “circadian” lighting systems that change light spectrum and intensity according to time of day, e.g., evening blue-depleted light [16]. A particular appeal of such innovations is that, once installed, the intervention is “low intensity” in as much as no additional clinical staff is required to manage the system. Our own research has shown that changing the evening light environment in an acute psychiatric unit has positive effects on the circadian, sleep and neurocognitive systems of healthy young adult volunteers [17,18]. However, it is unknown whether any change would translate into better clinical outcomes. Several mental health inpatient projects are underway, but there is limited research on the potential benefits of exposing trans-diagnostic inpatient populations to dynamic, programmable lighting conditions. To our knowledge, only 2 small-scale RCTs have been published [19,20]. Canazei and colleagues (2022) used actigraphy to monitor sleep and rest-activity rhythms in 30 individuals with depressive disorders admitted to inpatient rooms with circadian or standard lighting conditions. The study showed that although exposure to the experimental lighting was associated with some significant improvements in selected sleep and circadian metrics, the duration of admission did not differ between groups (20 versus 21 days). Okkels and colleagues (2020) randomly allocated 54 individuals admitted to an affective disorder unit to a pre-set circadian lighting condition where light intensity and spectrum is changed throughout the 24-h cycle or standard lighting environment. Most inpatients had an International Classifications of Diseases (ICD)-10 diagnosis of depression, but a minority had mania, anxiety, or personality disorders. The intervention group demonstrated nonsignificantly greater improvements in sleep quality and other clinical symptoms, and no differences in length of stay (22 versus 19 days in the control group).

In summary, uncertainties exist regarding the potential extent or magnitude of any improvements in clinical outcomes, including duration of general psychiatric acute admissions, that may be associated with exposure to evening blue-depleted light alongside usual inpatient care and treatment. The only consistent published finding is the lack of clinically significant side effects or adverse events. However, the small sample sizes and selective recruitment strategies mean that many inpatient subpopulations were underrepresented (e.g., psychotic disorders) [21]. Lastly, the trials of acutely ill patients employed eligibility criteria that de facto limited the generalizability of findings to real-world settings, such as the permanent exclusion of inpatients with severe symptoms, complex presentations, or impaired capacity to give immediate consent.

Given the above, we employed an ethically approved, deferred consent procedure that permitted acute admissions to be randomised to adult inpatient care in a ward with programmable dynamic lighting with a blue-depleted evening light environment (experimental group) or a similar ward with standard light environment (control group). This article reports the key findings. We aimed to examine any differences in mean duration of admission in days per individual according to group allocation (i.e., evening light environment) as the primary outcome, and any between-group differences in clinical symptom and function ratings, risk of or actual incidents of harm to self or others, patient satisfaction, and side effects as secondary outcomes.

## Methods

The reporting of this single-centre, two-arm, parallel-group, pragmatic effectiveness randomised controlled trial follows the CONSORT guidelines [22] (S1 CONSORT Checklist). The trial was an investigator-initiated study sponsored by St. Olavs Hospital, Trondheim University Hospital, Trondheim, Norway.

Here, we give an overview of the trial methodology while the online Supporting information provides other relevant information including the CONSORT checklist and further details of consent procedures, data management, and analyses (S1–S3 Files, S1 CONSORT Checklist, S1–S6 Tables, and S1 Fig).

### Ethics statement and registration

The trial was prospectively registered to the Regional Committee for Medical and Health Research Ethics in Central Norway, May 8, 2018 (REK 2018/946). The trial was approved on June 6, 2018 and all participants gave a written informed consent. Upon approval by the Committee, the trial registration and study protocol were made publicly available in a searchable database (see S1 File). The RCT was listed on the Current Research Information System in Norway July 19, 2018 (CRISTIN ID 602154). The initial trial registration and protocol (version 1) is available in the S1 File, p. 1. An updated protocol was submitted to the Committee on October 17, 2018 and was later registered on clinicaltrials.org and published (version 2, see S1 File p. 23). Due to logistical issues in the study group, and the need to start inclusion based on seasonal variations in daylight, the trial was retrospectively registered on clinicaltrials.org on December 28, 2018 (Clinicaltrials.gov NCT03788993; see S3 File, section A) which was updated with a detailed statistical snalyses plan (SAP) that was written and published prior to unblinding (see S2 File). No major changes were made to the study design between the prospectively registered protocol and the current manuscript.

### Study design and participants

The sample comprised individuals whose acute clinical presentation required inpatient care in the newly built 40-bedded adult psychiatric unit at St. Olavs Hospital, Trondheim, Norway (catchment area 300,000). The target was to recruit a minimum of 400 adults from the acute psychiatric admissions that occurred at any time and on any day of the week from October to March (to minimise the effect of seasonal variation in daylight; see S3 File, section B). There were 2 recruitment periods: October 23, 2018 to March 29, 2019 and October 1 to November 15, 2019 (see S3 File, section D).

An independent randomisation procedure was developed and managed by the Unit for Clinical Research at the Faculty of Medicine and Health Sciences, NTNU. As soon as the decision to admit an individual was confirmed, the person was randomly allocated to one of the 2 arms of the RCT on a 1:1 basis via a web-based programme using blocks of randomly varying size. Randomisation could be instigated at any time of the day or night without consultation with the hospital ward staff (regarding bed availability, case-mix, or staffing levels, etc.) and hospital staff could not influence the allocation procedure in any way.

### Eligibility criteria (pre- and post-randomisation)

To minimise exclusions, we employed a post-randomisation, deferred (delayed) consent procedure as utilised in many RCTs aiming to recruit representative samples of severely ill patient populations (also S1 File, p. 35) [23–25].

With ethical approval, the RCT eligibility criteria were as follows:

Inclusion criteria: All individuals aged > = 18 years who were admitted to the acute inpatient unit during the recruitment period were eligible for randomisation. Individuals who were re-admitted were also eligible for re-randomisation.

Exclusion criteria: There were no pre-randomisation exclusion criteria, but individuals could be withdrawn from the study post-randomisation.

Immediately post-randomisation, there were 2 options for withdrawing a participant from the RCT:

1. Lack of availability of rooms in the ward to which the individual was allocated, i.e., the randomisation process could not be completed.
2. Clinical imperative: a senior clinician decided that it was inappropriate to admit an individual to the room to which they are randomised. Reasons for which could be because admission to the allocated room might adversely affect the clinical case mix within the ward or compromise patient safety (see S3 File, section E for examples).

During the admission, or at the point of discharge, withdrawal from the RCT could occur because:

1. Lack of consent. The individual was unwilling to give written informed consent during an admission, according to the deferred consent procedure; was unable to give informed consent for the duration of the study, i.e., they persistently lacked mental capacity; or the consent procedure was incomplete: the individual had been discharged early or had an unplanned discharge so they were not approached about participation or had given verbal, but not written consent.
2. A patient could be withdrawn from the study if they were absent for >24 h from the ward to which they were randomised. This could be in instances where patients were transferred to a somatic hospital ward for several days; or clinicians instigated transfer to another psychiatric ward, etc.
3. An individual could decline to participate or withdraw their consent at any stage of the study and/or a mental health professional could recommend withdrawal of an inpatient from the RCT if they had any clinical concerns regarding an individuals’ participation. Potential reasons could be if a clinician believed a patient had experienced an RCT-related adverse event.

## Experimental and control conditions

**Fig 1:**
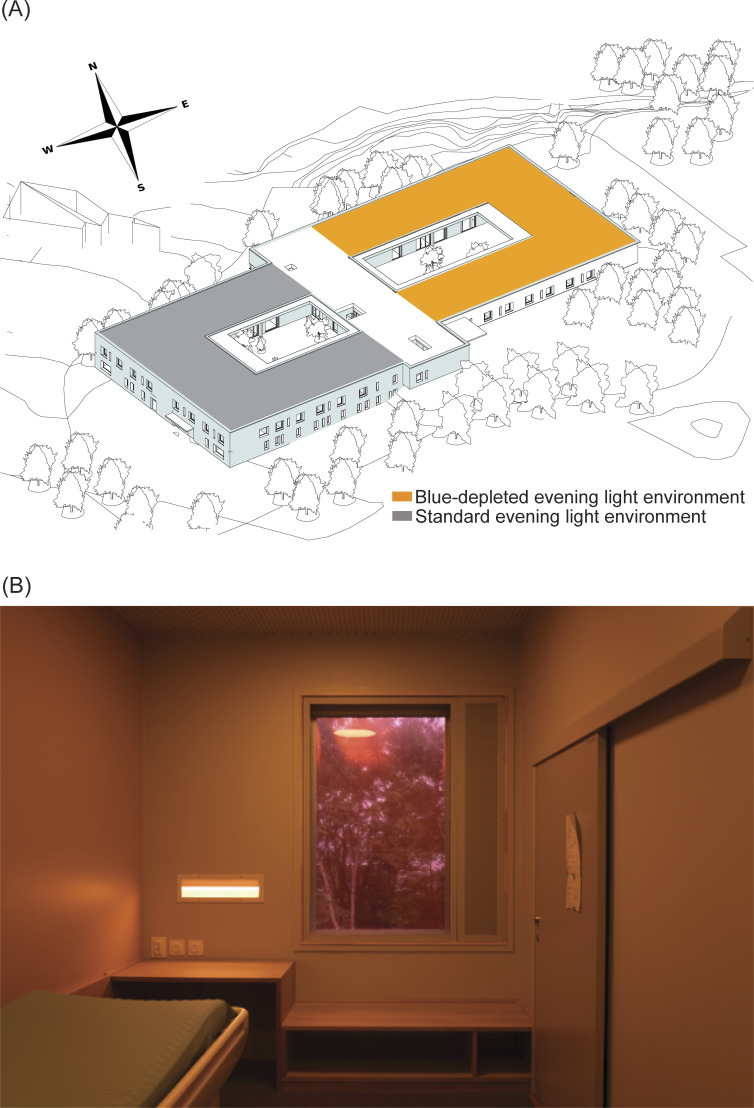
Unit layout. (A) Shows the layout of the unit. The experimental ward is shown in yellow and the control ward in grey. The 40 patient rooms are located along the outer walls, and the common areas face the atriums and are divided into 2 wards. There are 8 rooms facing east in the experimental ward, whereas 6 rooms in each ward face north or south. All patient rooms are located on the top floor. The white area is reserved for dining and personnel rooms. (B) Shows a patient-room in the evening blue-depleted light environment.

The construction of the 40-bedded psychiatric inpatient unit was initiated in 2015 and finished in December 2017. The unit is divided between 2 wards built around 2 atriums (Fig 1). Each hospital ward has the same staffing levels, layout, and facilities and 5 rooms in each ward are designated as “psychiatric intensive care” beds. The staff rotated between the wards every 6 weeks.

The lighting fittings and fixtures were identical in both wards (Glamox AS, Oslo, Norway), while the diodes were different. From 07:00 h to 18:00 h, the light spectrum and intensity was similar in the experimental and control conditions. From 18:00 h to 07:00 h, the light intensity (photopic lux) was similar in both wards, but individuals were exposed to a different spectrum of evening light in each ward. While light exposure will vary with the direction of gaze, we performed light assessments using a Mavospec Base light metre (Gossen Foto- und Licht messtechnik GmbH, Nürnberg, Germany) horizontally at eye level (160 cm) at standardised locations in both wards. The melanopic EDI was assessed to be between 7 and 21 in different rooms of the blue-depleted ward, whereas it was assessed to be between 27 and 63 at the same locations in the standard light environment. The standard light environment, see Table 1 in Vethe and colleagues [18] for details about the composition of the evening LE in the 2 wards. We confirmed that LE was similar to Vethe and colleagues [18] before the recruitment was initiated.

(a) Experimental condition (Blue-depleted Evening Light Environment): The ward had tuneable light emitting diode (LED) fixtures and the amount of blue light in the ward was tested prior to commencing the RCT. At 18:00 hours (h), the lighting underwent a 30-min transition during which the green and blue LEDs were dimmed to produce blue-depleted amber coloured lighting. At 06:50 h, a 10-min transition programme changed the light colour to normal indoor lighting (3,000 Kelvins of colour temperature) which then continued until 18:00 h. The light intensity was dimmed to 20% (of the maximum) from 23:00 h to 06:50 h.

As well as the LED system, blue-blocking window filters were deployed in the evening. All television sets had permanent blue-blocking filters, and the outdoor area had external lights that block blue light. Use of electronic media was not restricted (unless an individual treatment plan limits access), but patients were provided with blue-blocking screens that could be attached to the front of all electronic devices (lowbluelights.com). If a patient left the blue-depleted unit after 18:30, they were offered blue-blocking glasses to wear (circadianeyewear.com).

(b) Control condition (Standard Light Environment): The ward had normal indoor hospital lighting installed. The light intensity was dimmed to 20% (of the maximum) during the night (from 23:00 h to 06:50 h). The light spectrum remained constant throughout the 24-h cycle at 3,000 K.

**Table 1:** Summary of participant demographics and clinical characteristics for patients in the Intention to Treat population, randomised to admission in a standard light environment or evening blue-depleted light environment.

Variable	Blue-depleted evening light environment (*n* = 232)		Standard light environment (*n* = 244)		Total (*N* = 476)	
*Demographic information*						
Sex, male, *n* (%)	102	(44.0)	91	(37.3)	193	(40.5)
Age, years, mean (SD)	37.1	(13.3)	37.2	(14.4)	37.2	(13.9)
Ethnicity, European, *n* (%)	189	(81.5)	180	(73.8)	369	(77.5)
Education, primary school only, *n* (%)	44	(19.0)	49	(20.1)	93	(19.6)
Employment status, full-time, *n* (%)	39	(16.8)	52	(21.3)	91	(19.1)
Living situation, no fixed abode, *n* (%)	17	(7.3)	15	(6.1)	32	(6.7)
*Diagnostic data for ICD-10 mental disorder diagnoses*, *criteria for research*						
Number of diagnoses (*n*, %)						
0	21	(9.1)	12	(4.9)	33	(6.9)
1	123	(53.0)	145	(59.7)	268	(56.4)
2	65	(28.0)	64	(26.4)	129	(27.2)
3+	23	(9.9)	22	(9.0)	45	(9.5)
Primary diagnoses, according to ICD-10 chapters, *n* (%)						
Affective disorders	60	(25.9)	61	(25.1)	121	(25.2)
Psychotic disorders	36	(15.5)	51	(20.9)	87	(18.3)
Personality disorders	23	(9.9)	32	(13.2)	55	(11.6)
Addiction disorders	41	(17.7)	29	(11.9)	70	(14.7)
Anxiety disorders	24	(10.3)	29	(11.9)	53	(11.2)
Organic disorders	7	(3.0)	11	(4.5)	18	(3.8)
*Other clinical characteristics*						
Duration of illness episode before admission < = 1 month, *n* (%)	89	(36.4)	84	(34.4)	173	(36.3)
Somatic comorbidity, *n* (%)	66	(28.4)	55	(22.6)	121	(25.4)
Involuntary admission, *n* (%)	40	(17.2)	42	(17.2)	82	(17.2)
Admission last 2 years > = 1, *n* (%)	108	(46.6)	114	(46.7)	222	(46.6)
Previous criminal conviction, *n* (%)	18	(7.8)	18	(7.4)	36	(7.6)
Violence towards others past year, *n* (%)	15	(6.5)	19	(7.8)	34	(7.2)
Suicidality (> = 1 previous suicide attempt), *n* (%)	74	(31.9)	62	(25.4)	136	(28.6)
Baseline CGI-S, median, (IQR)	5	(4 to 5)	5	(4 to 5)	5	(4 to 5)
Sleep-wake disruption *n*, (%)*	112	(48.3)	97	(39.8)	209	(44.0)

Percentages are reported for all individuals regardless of missingness which ranged from 0% for many demographic variables to 41% for previous suicide attempt (detailed baseline characteristics found in S2 Table).

*Sleep-wake disruption = Difficulties falling asleep, nocturnal awakenings, or early morning awakenings; 3 or more times per week.

IQR, interquartile range; ICD, International Classification of Disease; CGI-S, Clinical Global Impression—Severity sub-scale (Severity at discharge); SD, standard deviation.

### Assessments

Full details of all assessments employed in the main and ancillary studies are reported in the published protocol [1]. Here, we briefly describe the assessments employed in the RCT.

### Diagnosis

A preliminary diagnosis/diagnoses was recorded at admission, but the analyses used the consensus discharge diagnosis/diagnoses of mental disorders according to the ICD-10 “criteria for research” (Chapter F) (World Health Organization, WHO) [26].

### Baseline assessment

At intake, the following information was recorded:

- Age, sex, ethnicity, marital status, living situation, years of education, and employment status.
- Other key characteristics include type of admission (voluntary or involuntary), current alcohol and substance use, risk of or actual harm to self or others, physical health status, disrupted sleep (operationalised as disturbances > = 3 nights per week the last 30 days before admission), and medications used before/at admission (categorised according to the WHO class of medication; S1 Table).
- Details of past psychiatric history, specifically recording total number of psychiatric admissions and number of inpatient bed-days in the 2 years prior to the index admission, and forensic history (including history of violence).

### Primary outcome

The primary outcome was mean duration of admission in days per individual. Admission was defined as the time and date of the initiation of the intake assessment. Time of discharge was defined as midday of the day the patient left the light environment to which they were randomised for >24 h. Duration of admission was calculated as the date and time of discharge minus the date and time of admission. Patients who remained admitted 14 days after the conclusion of each recruitment period were considered to have an admission duration equal to their current length of stay at that date.

### Secondary outcomes

#### Clinical outcomes

(a) Clinical Global Impression (CGI)

The CGI is a well-established, practical measurement tool that is widely used in RCTs, including studies of inpatient and trans-diagnostic samples [27–29]. The CGI has 2 components, the improvement and severity scales, which together are used to quantify and track clinical progress and outcome [30].

Clinical Global Impression, Improvement subscale (CGI-I): We used the improved version of the CGI-I [31]. Scores can range from −6 (maximum deterioration) to +6 (ideal improvement), with a higher CGI-I rating indicating greater improvement relative to baseline status. The CGI-I was rated at discharge. A change of 4 or more on the CGI-I denotes a considerable improvement that is clear and clinically meaningful [31].

Clinical Global Impression, Illness Severity scale (CGI-S): The CGI-S is rated on a 1–7 Likert with high scores indicating worse clinical status and/or functioning [30]. The instruction states: Considering your total clinical experience with this particular population, how mentally ill is the patient at this time? (1 = Normal, not at all ill; 7 = Among the most extremely ill patients). A CGI-S rating of 3 or less denotes that the individual is mildly unwell or better relative to other acutely admitted patients [30]. The CGI-S was rated at admission and discharge.

(b) Risk of harm to self or others

Broset Violence Checklist (BVC): Aggressive behaviour was assessed 3 times per 24 h using the 6-item BVC assessing the presence of 6 specific behaviours (e.g., irritability, physically, and verbally threatening behaviour) on a binary scale (0 = not observed, 1 = observed). We analysed BVC sum scores > = 2 which has been shown to be a severity of aggressive behaviour which predicts short-term risk of inpatient violence [32,33].

Staff Observation Aggression Scale-Revised (SOAS-R): Incidents of actual violence were systematically recorded using the SOAS-R [34] after an incident had occurred. The SOAS-R total score ranges from 0 to 22 and a score > = 9 indicates that more serious incidents have occurred (e.g., inflicting physical pain or injury).

Suicide Risk: Suicide risk was assessed and recorded daily. At the daily staff meetings, the staff categorised suicidality as present or not. This was an assessment of high suicide risk over the next 24 h based on national guidelines by the Norwegian Directorate of Health [35], in addition to local guidelines and evaluations of the current clinical state.

Required supervision due to risk of suicide: Increased supervision could be required if there was a need for more frequent observations than every 30 min throughout the 24 h (i.e., every 5, 10, or 15 min, or continuous observation of the patient)

(c) Change in admission status

For patients who were involuntarily admitted, we assessed the time from intake to when admission status was changed from involuntary to voluntary admission.

(d) Side effects

The frequency and severity of side effects was rated on a 4-point scale using 8 items measuring side effects of acute psychiatric treatments (e.g., difficulties with concentration, change in appetite) supplemented by the Headache and Eyestrain Scale (e.g., headache, dry eyes) [36].

(e) Self-reported satisfaction

Individuals routinely rate their satisfaction with an admission using a standard 11-item questionnaire (each item is rated from 1–5, with higher scores indicating greater satisfaction).

### Statistical methods

The analyses reported in the main text refer to the intent to treat (ITT) population which included all randomised participants who ultimately consented to the trial and were admitted to their assigned ward.

### Sample size calculations

The sample size calculation is described in detail in the published protocol [1]. Briefly, the calculation was performed for the comparison of the primary outcome, mean number of days hospitalised per individual exposed to the experimental or control lighting conditions. Assuming the experimental lighting conditions led to a reduction in the mean length of stay of 1 day [8], from about 6 to 5 days (with a standard deviation (SD) of about 3.5 days), then 194 participants in each condition would give an 80% chance at an alpha = 0.05 to detect a difference in the length of stay of 1 day using an ITT analysis.

### Statistical analysis

A detailed SAP was written and published prior to the unblinding of the data set, and the data were analysed according to this plan by statisticians prior to unblinding the data set (see S2 File). Multivariable linear regression was used to analyse the effect of blue-depleted evening lighting on the duration of admission in days (primary outcome). These analyses were adjusted for a prespecified set of baseline variables considered to be predictors of length of admission, including age, sex, psychiatric diagnosis, comorbid personality disorder, or substance use disorder, whether the admission was voluntary or not, and the number and duration of previous admissions in the past 2 years. For the main analysis, the psychiatric diagnosis was categorised as either psychotic episode/disorder, manic episode, severe depression, or other (see SAP for further details). As expected, duration of admission was right-skewed, and we used bootstrapping to obtain the 95% confidence interval (CI) with the bias-corrected and accelerated (BC_a_) method and B = 5,000 bootstrap samples. Time to discharge for all participants and in diagnosis subgroups are also illustrated using Kaplan–Meier curves.

To assess if the effect of the blue-depleted evening lighting differed between subgroups, the regression analyses were repeated with an interaction term between each of the covariates listed above and randomisation group. A Wald test was used to jointly test the interaction term coefficients when there was more than 1 category/subgroup included.

Secondary outcomes were analysed using linear regression models with the same covariates and bootstrapping approach as the primary variable: CGI-I and CGI-S at discharge, number of episodes with a BVC of 2 or more, number of episodes with a SOAR-S score of 8 or more, and side effects and satisfaction score. The remaining secondary outcomes were binary variables and were analysed using logistic regression with the same covariates as above. Additionally, for CGI-S the baseline value of this variable was included as a covariate. For change from involuntary to voluntary admission, we additionally compared the lighting groups using a Cox regression model. The average score for each of the side effects scales are compared using two-sample *t* test, excluding individuals missing more than 2 items.

Post hoc analyses: We estimated the Number Needed to Treat (NNT) to enable the investigators to interpret the clinical meaningfulness of any statistically significant between-group differences in key outcome measures (see S3 File, section F, for details of how it was calculated). We chose the NNT because it is widely used across medicine to communicate the effectiveness of different health care interventions and represents the average number of patients who need to be treated to prevent one additional bad outcome [37]. In this RCT, it is the number of patients that need to be exposed to the experimental lighting condition for one patient to benefit compared with the control condition. The user written package bcii in Stata/MP 18.0 was used to calculate the NNT with 95% CIs. This package employs the confidence interval calculation methods described by Bender (2001) [38]. Additionally, a post hoc analysis was completed to assess if the results are affected by participants who remained admitted after data collection ceased (S3 File, section H).

### Study monitoring

A Data and Safety Monitoring Committee (DSMC) scrutinised trial progression, technical issues, and the safety of patients in weekly meetings and the DSMC offered guidance on the resolution of 3 issues (S3 File, section G).

## Results

**Fig 2:**
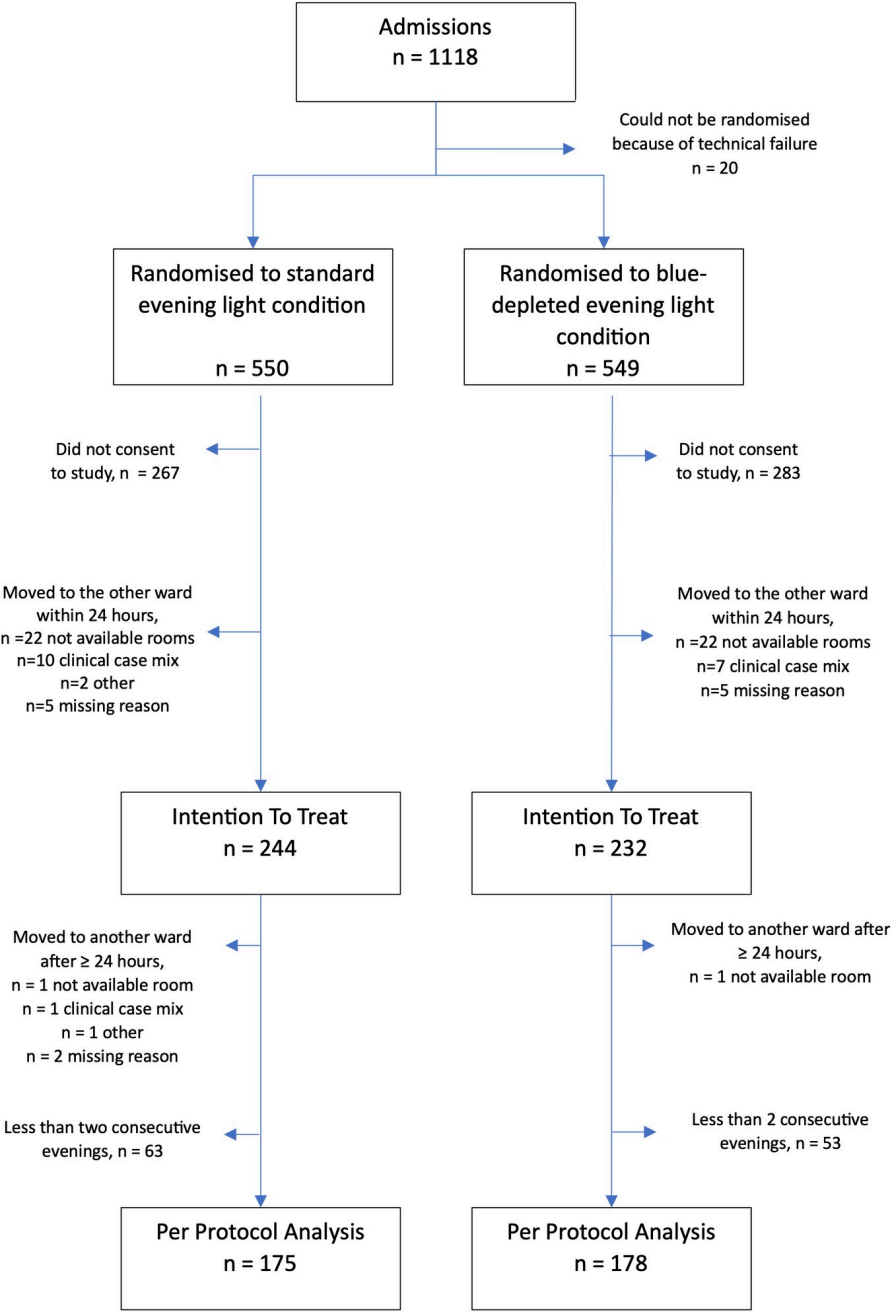
CONSORT flow-chart.

**Fig 3:**
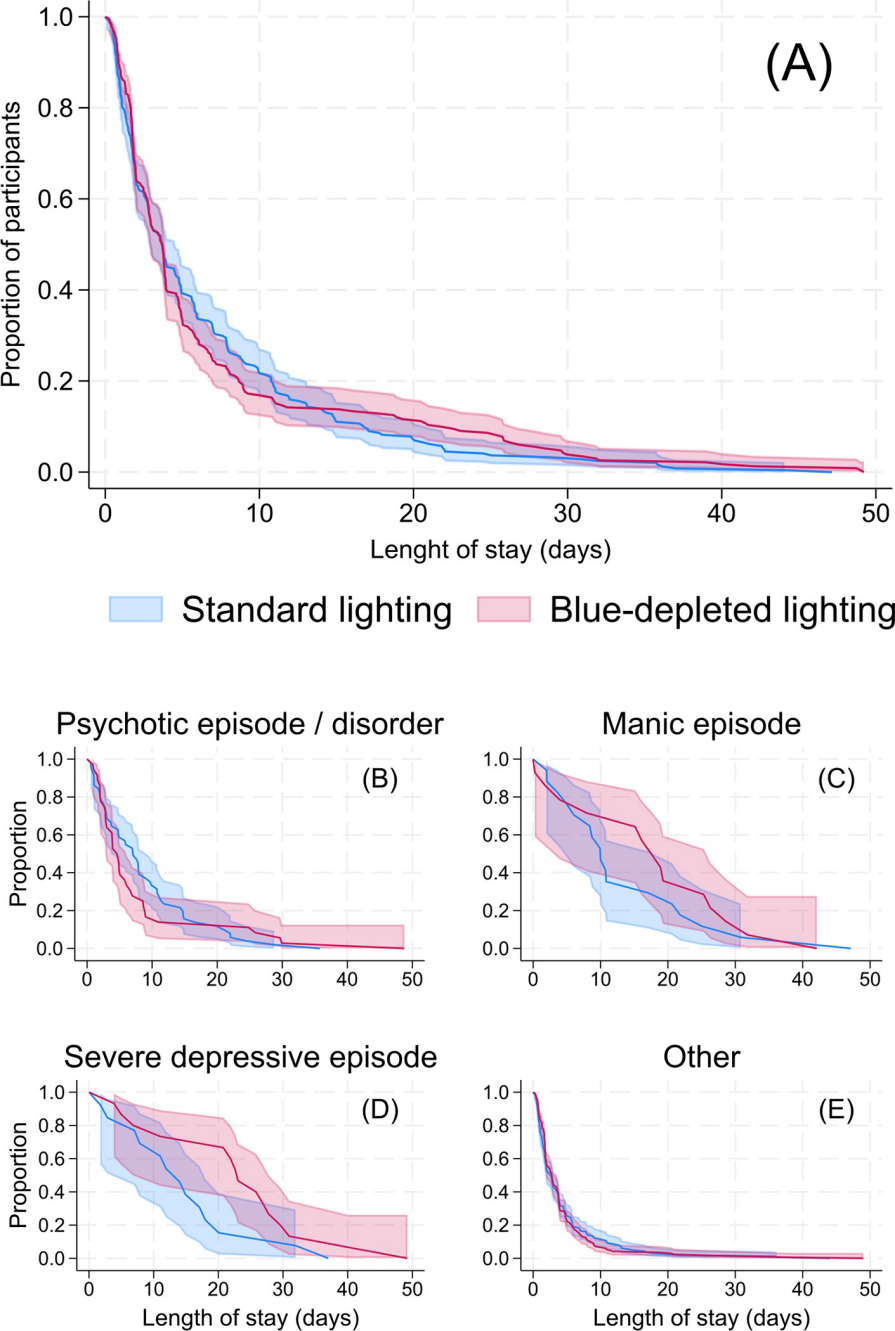
Proportion of patients by duration of admission for preselected diagnostic categories. Red line and shaded area = Evening blue-depleted light environment with 95% CI. Blue line and shaded area = Standard light environment with 95% CI. (A) Data for all participants (*N* = 476). (B) Data for patients diagnosed with a psychotic episode (*n* = 87). (C) Data for patients diagnosed with a manic episode (*n* = 31). (D) Data for patients diagnosed with a severe depressive episode (*n* = 28). (E) Data for patients with all other diagnoses (*n* = 329). There were no statistically significant differences in the effect of the evening blue-depleted light environment across the subgroups (*p*-value for interaction = 0.239).

**Fig 4:**
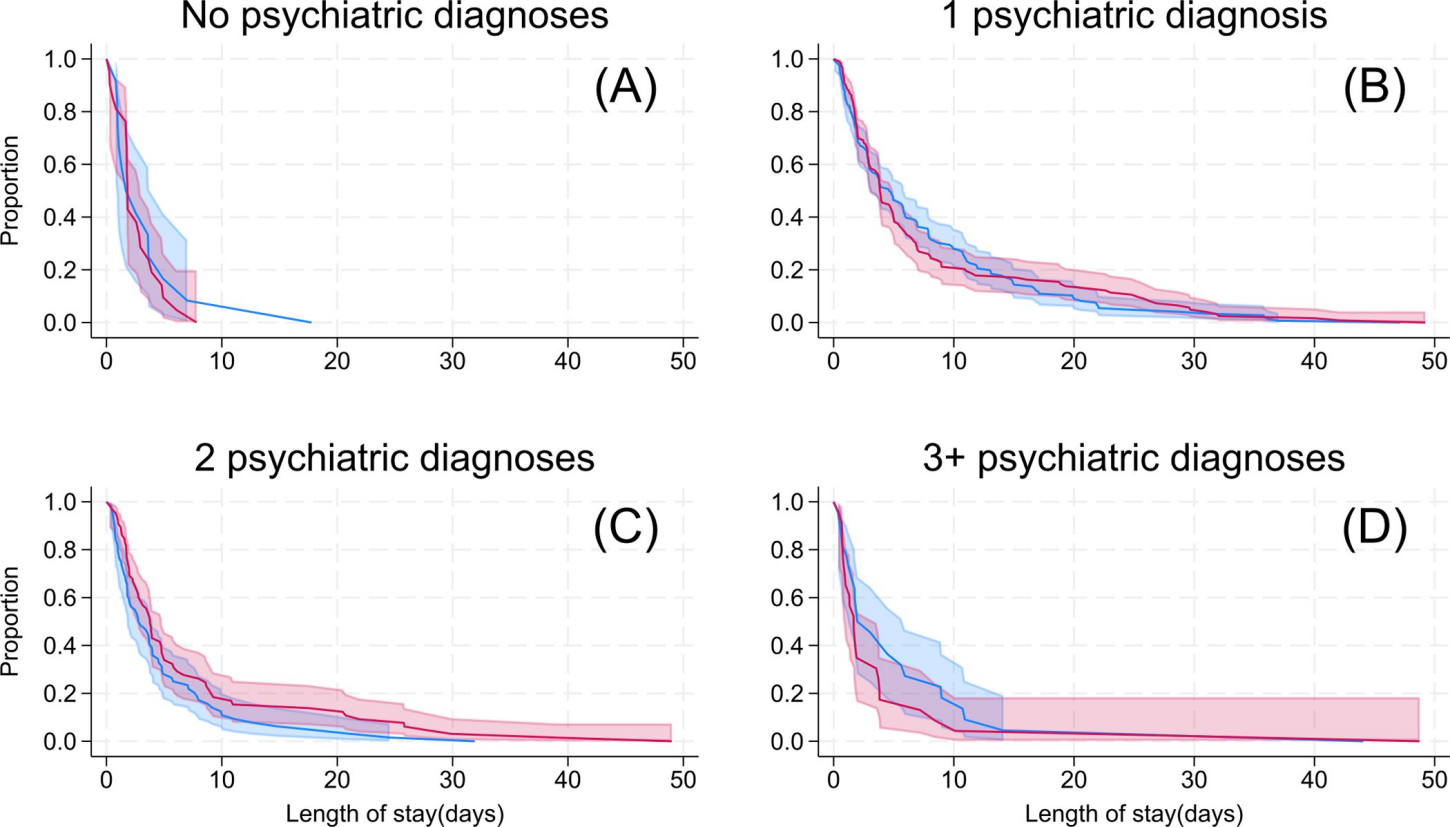
Proportion of patients by duration of admission for number of psychiatric diagnoses. Red line and shaded area = Evening blue-depleted light environment with 95% CI. Blue line and shaded area = Standard light environment with 95% CI. (A) Data for no psychiatric diagnoses (*n* = 33). (B) Data for patients with one psychiatric diagnosis (*n* = 268). (C) Data for patients with 2 psychiatric diagnoses (*n* = 129). (D) Data for patients diagnosed with 3 or more psychiatric diagnoses (*n* = 45). There were no statistically significant differences in the effect of the evening blue-depleted light environment across the subgroups (*p*-value for interaction = 0.209).

Fig 2 provides an overview of the flow of participants through the clinical trial. The core characteristics of the ITT sample and key clinical outcomes are summarised in Tables 1 and 2 and Figs 3 and 4 (see online Supporting information for additional details for all findings: S1–S6 Tables and S1 Fig).

**Table 2:** Primary and secondary outcomes for patients (*N* = 476) in the Intention to Treat population, allocated to admission in a standard light environment (*n* = 244) or evening blue-depleted light environment (*n* = 232).

			Blue-depleted evening light environment	Standard light environment		
**Linear regression**		***N***	**Estimated mean** [**95% CI**]	**Estimated mean** [**95% CI**]	Mean difference [95% CI]	***p*-value**
**Length of stay (days)**		**476**	**7.1** [**6.1, 8.1**]	**6.7** [**5.8, 7.5**]	0.4 [−0.9, 1.8]	**0.523**
**Clinical improvement during admission (CGI-I)**		442	2.13 [1.94, 2.31]	1.85 [1.67, 2.03]	0.28 [0.02, 0.54]	0.035
**Clinical state at discharge (CGI-S)**		443	3.37 [3.26, 3.48]	3.55 [3.43, 3.67]	−0.18 [−0.34, −0.02]	0.029
**Aggressive behaviour (BVC)**						
No. of serious events		475	0.04 [0.00, 0.09]	0.29 [0.17, 0.41]	−0.25 [−0.38, −0.12]	<0.001
No. of serious events/100 days		475	0.31 [0.00, 0.71]	3.30 [1.34, 5.27]	−2.99 [−5.00, −0.98]	0.004
**Violent incidents (SOAS-R)**						
No. of serious events		476	0.18 [0.04, 0.32]	0.12 [0.05, 0.18]	0.07 [−0.09, 0.23]	0.414
No. of serious events/100 days		476	0.97 [0.19, 1.75]	1.79 [0.34, 3.24]	−0.82 [−2.5, 0.91]	0.353
**Side effects**						
	Headache and eye strain scale	215	1.99 [1.87, 2.11]	1.94 [1.82, 2.06]	−0.04 [−0.21, 0.12]	0.599
	Other side effects	217	2.00 [1.90, 2.09]	2.10 [1.99, 2.18]	0.09 [−0.05, 0.22]	0.199
	Total side effects	217	1.99 [1.89, 2.09]	2.03 [1.93, 2.13]	0.04 [−0.09, 0.18]	0.532
**Patient satisfaction** ^a^		205	3.77 [3.62, 3.91]	3.66 [3.52, 3.80]	0.10 [−0.10, 0.31]	0.322
**Logistic regression**		***N***	**Estimated percentage** [**95% CI**]	**Estimated percentage** [**95% CI**]	**aOR** [**95% CI**]	***p*-value**
**Risk of suicide**		412	42.2 [36.1, 48.3]	40.8 [34.8, 46.8]	1.07 [0.69, 1.67]	0.752
**Required supervision due to risk of suicide**		412	17.8 [13.0, 22.7]	20.1 [14.9, 25.2]	0.85 [0.49, 1.44]	0.538
**Change from involuntary to voluntary status**		79^b^	27.5 [15.8, 43.4]	47.6 [33.0, 62.7]	0.43 [0.13, 1.42]	0.167
			**Median time to change in admission status (IQR)**	**Median time to change in admission status (IQR)**	**aHR** [**95% CI**]	***p*-value**
**Time to change from involuntary to voluntary status (days)**		82^b^	31 (11, 73)	19 (10, 51)	0.73 [0.30, 1.84]	0.513

^a^Ten-item satisfaction score with each item score from 1 to 6, averaged across all items with a response so long as 9 of 10 items are answered.

^b^The adjusted logistic and Cox regression analyses includes only those who were admitted involuntarily at the review on day 2, additionally all 3 participants with admitted involuntarily with depression episode subsequently ended up with a voluntary admission status and were excluded from the logistic regression.

iCGI-I, Clinical Global Impression Scale—Improvement (clinicians’ assessment of improvement at time of discharge); CGI-S, Clinical Global Impression—Severity subscale (Severity at discharge); BVC, Broset Violence Checklist (score > = 2 considered “severe”); SOAS-R, Staff Observation Aggression Scale-Revised (score > = 9 considered severe); IQR, interquartile range; CI, 95% confidence interval; aOR; adjusted odds ratio; aHR, adjusted hazard ratio.

### Sample characteristics

As shown in Table 1, the ITT sample comprised 476 individuals (Blue-depleted light environment = 232; Standard light environment = 244). The sample mean SD age was 37.2 (13.9) years, 193 were male (41%), 369 (78%) were of European origin, and 91 (19%) were full-time employed. The most frequent primary diagnoses were: affective disorder (*n* = 121; 25%), 31 of which were in a manic episode and 28 had a severe depressive episode and psychotic episodes (*n* = 87; 18%). Thirty seven percent of the sample (*n* = 174) received 2 or more ICD-10 diagnoses. At admission, the sample mean CGI-S rating was 4.6 (median 5, interquartile range 4 to 5) and 174 (37%) had been unwell for > = 30 days. See S2 Table for details.

There were 121 (25%) individuals with at least 1 somatic condition and 209 (44%) reported current sleep disturbances. Notably, there was no record that 299 (63%) of the sample were receiving psychotropic medications at the time of admission. Of those receiving medications, the most frequently used medications were: antipsychotics (*n* = 129, 27%), benzodiazepines/z-hypnotics (*n* = 59, 12%), and anti-depressants (*n* = 56, 12%).

### Clinical outcomes

As summarised in Table 2, there were no statistically significant differences in the duration of admission in individuals allocated to the evening Blue-depleted light environment (Mean: 7.1, CI [6.1, 8.1]; Median: 3.71) as compared with the Standard light environment (Mean: 6.72; CI [5.8, 7.5]; Median: 3.68) estimated mean difference: 0.4 days CI [−0.9, 1.9]; *p* = 0.523, nor in the effect of lighting environment in the duration of admission according to diagnostic subtypes (*p* = 0.22) or number of diagnoses per individual (*p* = 0.21) (Figs 3 and 4). See S3 Table for observed values and S4 Table for subgroup analyses for duration of admission. There were no relevant differences in duration of admission between the ITT and Per Protocol samples (see S5 Table) or after extending the follow-up period for participants who remained admitted after data collected concluded (S6 Table).

Compared with the Standard light environment, individuals allocated to the Blue-depleted light environment showed significantly higher improvement during admission (Clinical Global Impressions scale-Improvement, CGI-I, estimated mean difference: 0.28 (95% CI [0.02, 0.54]; *p* = 0.035), NNT: 12 (95% CI [6, 61]) and lower illness severity at discharge (Clinical Global Impressions Scale-Severity of illness, CGI-S, estimated mean difference: −0.18 (95% CI [−0.34, −0.02]; *p* = 0.029), NNT: 7 (95% [CI 4, 22)]).

The BVC ratings of aggressive behaviours were significantly lower in the Blue-depleted light environment compared with the Standard light environment. The estimated difference in predicted serious events per 100 days: −2.99 (95% CI [−5.00, −0.98]; *p* = 0.003), NNT: 9 (95% CI: [7, 15]).

There were no significant between-group differences in actual violence episodes (estimated mean difference: 0.07 (95% CI [−0.09, 0.23]; *p* = 0.414)), risk of suicide (estimated mean difference: 1.07 (95% CI [0.69, 1.67]; *p* = 0.752)), or the probability of changing from involuntary to voluntary status (estimated mean difference: 0.43 (95% CI [0.13, 1.42]; *p* = 0.167)). Likewise, side effects (estimated mean difference: 0.04 (95% CI [−0.09, 0.18]; *p* = 0.532)) (see also S1 Fig for details) and satisfaction ratings did not differ between groups (estimated mean difference: 0.10 (95% CI [−0.10, 0.31]; *p* = 0.322)) (Table 2).

## Discussion

In this RCT, we tested if there are any benefits of modifying the evening light environment in an acute psychiatric hospital according to chronobiological principles. We found no differences between the Blue-depleted light environment and Standard light environment in our primary outcome of duration of admission. However, there were benefits observed by clinicians on ratings of clinical improvement (CGI) and aggressive behaviour (BVC). In terms of clinical significance, the NNTs for greater clinical improvements and lower levels of aggressive behaviour are similar to other low intensity interventions employed in psychiatry and in general medicine that are included in NICE guidelines. Further, these clinical benefits were achieved without any discernible differences in patient-rated side effects or treatment satisfaction levels. Moreover, we did not find any association with lighting conditions and transition from involuntary to voluntary status, nor any differences in actual violent incidents or suicidality, though the latter were low-frequency events, and it is likely that the trial was insufficiently powered to detect such events.

We acknowledge that our primary outcome measure and the design of this RCT was ambitious. Nevertheless, we believe these choices were justified as inpatient admissions are the largest contributor to the cost of psychiatric care. A reduction in occupied bed-days would not only be welcomed by patients but also offset the cost of new lighting systems. Further, we wanted to test if there were any benefits of the lighting conditions in a real-world clinical setting. A defining characteristic of a public health system that takes all admissions from a defined catchment area is that the inpatient population is heterogeneous, and the throughput of admissions is rapid, with short lengths of stay being the norm. This was true of our study setting with 1,118 admissions over 8 months and a median duration of admission of only about 4 days. Employing a pragmatic effectiveness design with deferred consent meant that this RCT recruited nearly half of all the patients admitted from a population of 300,000 people, including involuntary admissions and many other individuals with severe or complex problems who would normally be excluded from research studies. As such, this RCT has greater external validity, and findings are more generalizable, than efficacy studies undertaken in specialist clinics and other selective research settings that tend to recruit small homogenous samples.

While we set a very high threshold for finding between-group differences in the duration of acute admissions, it is notable that this is the third RCT exploring lighting in psychiatric inpatient settings that has failed to find a significant reduction in the length of admission [19,20]. Length of acute admission and decisions about hospital discharge are not only based on the clinician and patient observations regarding progress, but also on structural and practical issues, such as vacancies for suitable placements at other hospital facilities units and the need to accommodate new acute admissions. While the duration of admission was an obvious choice as the primary outcome, it is also true that these other factors may have reduced the precision of the findings. The absence of significant between-group differences in outcomes related to involuntary status, suicidality or violent incidents may be explained as these were low-frequency events, which reduced the statistical power to find differences. As such, we can only conclude that exposure to the blue-depleted light environment did not exacerbate any of these problems.

The key differences we identified between the light environments were that exposure to the blue-depleted light environment was associated with better clinical evaluations of outcome, greater improvement, and lower levels of aggression during admission. The NNTs were between 7 and 12 for these outcomes, which matches that of other low-intensity interventions in clinical psychology or medicine [39,40]. The CGI-S and CGI-I are widely employed in RCTs in inpatient units and have clearly defined anchor points [27,28,30,31], and the BVC is a measure of observed behaviour that has been validated and implemented in more than 20 countries [41]. Our findings, therefore, suggest some cause for optimism that modifying the evening light environment in a psychiatric inpatient unit could benefit a wide range of acute admitted inpatients. However, we propose two potential research directions that may enhance the outcomes in the future: (i) determining the optimal intervention dosage and enhancing adherence; and (ii) balancing global versus specific outcome measures and beginning to disentangle moderators and mediators of response.

We have previously shown that the same light environment as employed in the current RCT produced changes in sleep, circadian rhythms, and arousal in healthy young adults [17,18]. Further, there were no negative effects in that population, nor in nurses’ ability to work in the blue-depleted light environment at the present unit [42], or elsewhere [43]. However, it is possible that aspects of the “dosage” we employed are suboptimal. The evening light in the current trial was between 7 and 21 melanopic lux [18], whereas new guidelines suggest that 10 melanopic lux is the maximum intensity to avoid negative impact on the circadian system in healthy individuals [44]. The melanopic lux we employed is lower than in previous trials [19,20], but we were mindful that patients in an acute psychiatric ward may have an altered light sensitivity compared with the normal population [45–48]. Moreover, we note that Henriksen and colleagues found a large effect on manic symptom severity after 7 days of evening use of BBG as an adjunct to standard treatments, which is a longer exposure than most patients in our trial. It has also been argued that combining chronotherapeutic interventions may be synergistic [21,49], although our ambition was to test the isolated benefit of only modifying the evening light environment. It will be important for future research to test if changing the dose by increasing the number of days the patients are exposed and/or by adding other chronotherapeutic interventions, such as morning bright light exposure in non-manic patients, will influence key clinical outcomes.

On a related theme to dosage, is the issue of adherence with the intervention. Previous trials of psychiatric hospital lighting have been limited to single hospital rooms [20] or have had limited control over other light sources [19] that could impact adherence. We had a specific focus on securing adherence, such as installing the light environment in patient rooms, bathrooms, and common areas in unit, deploying filters in front of windows in patient rooms after 18:10 h and permanent filters on TV screens, and offering blue-blocking filters on mobile devices. However, due to the unit’s location close to the Arctic Circle, daylight varies substantially throughout the year. Therefore, we included patients over a fixed season from October to April as continued recruitment in the summer half-year would introduce a bias where we could not control the bright light exposure in the morning or evening. Although the RCT design would cause this to be evenly distributed in the two light environments, it could introduce bias specifically to the experimental condition in this study. First, patients would be exposed to daylight if they entered the atriums in the evening, which could reduce the effects of the evening blue-depleted light environment. To mitigate this issue, lights in the outside recreation/smoking area were blue-depleted and BBGs were available at the doors when leaving the ward. However, patients may have chosen not to use the BBG, and other adherence issues could have attenuated the observed effects. It is currently unknown how high exposure to ordinary evening light is sufficient to disrupt any clinical effect of evening blue depletion. However, a 15-s pulse of bright light exposure (9,500 lux) has been found to induce circadian phase delays in healthy individuals [50]. Secondly, there were 8 rooms in the experimental ward that faced east that could receive morning light exposure during the summer half-year, which could selectively impact the clinical outcomes for patients allocated to these rooms [51]. Finally, it would not be feasible to continue recruitment to an RCT in an acute ward during a time with vacation substitutes for the original staff. Thus, the trial results may not generalise to times of the year when sunset occurs late in the evening or at night.

Many RCTs of neuropsychiatric patients employ CGI ratings to measure outcomes, even in disorder-specific studies [27,29,52,53]. The advantage is that it is a clinically valid measure that clinicians can incorporate into day-to-day practice with minimal disruption [30]. These global outcome measures are especially useful in large-scale pragmatic RCTs with heterogenous samples. The 476 participants in the current trial reported 125 different ICD-10 primary and secondary diagnoses with many presenting with comorbid mental and physical disorders. It was unfeasible to employ disorder-specific rating scales for such a broad and complex range of clinical presentations. However, using global outcome measures to assess the benefits of relatively low-intensity interventions risks missing subtle but important effects the intervention may have on both specific symptoms and/or transdiagnostic processes such as sleep-wake regulation [13]. Further studies should strive to determine how specific patient characteristics, including mental disorder type, nature of sleep-wake disturbances, illness episode duration, and the interaction with certain medications [45–48,54,55] may influence the efficacy of the intervention. Additionally, age-related factors could also modulate the intervention’s effectiveness [56]. This complex array of potential moderators underscores the challenges clinicians currently face in assessing patient suitability for this intervention and may ultimately influence whether the intervention is employed in general psychiatric settings or restricted to specialist units.

In addition to the above issues, other limitations should be acknowledged. First, the nature of this study meant it was impossible to blind patients or clinical staff to the lighting condition, but although we cannot rule out a potential bias, we have no evidence that conscious or unconscious bias would explain these findings, either in the decision to discharge a patient or in any of the clinical evaluations. Moreover, we cannot know specifically the degree to which the intervention may have affected those recording the outcomes, as opposed to the subject participants. Related, we found significant differences in the observations of behaviours and evaluations of clinical staff, but we did not find significant differences in objective outcomes of violent incidents. However, violent incidents were low-frequency events to which the trial was insufficiently powered, and we note that although it did not reach significance, the number of registered incidents was 80% higher in the standard light environment. The primary outcome was heavily skewed. As described in the pre-publication Statistical Analytical Plan, this was expected, and bootstrapping was used to address this issue. The use of deferred consent meant that some patients left the unit before they could be approached regarding study participation and others did not sign the consent form. Therefore, about 50% of all admitted patients were included in the ITT population. There was a variable degree of data incompleteness at baseline, which may be attributable to the patients’ compromised capacity to provide data.

Changing the evening light environment moderately enhances the clinical outcomes of standard acute psychiatric hospital inpatient care and treatment with estimated NNTs between 7 and 12 for clinical state, improvement, and aggressive behaviours. These benefits, coupled with the absence of side effects and the low intensity of the intervention, indicate that there is a reasonable case for broader adoption of this strategy, particularly in new units where the lighting system is being installed for the first time and is not replacing previous systems. Of course, further research is required on the nature and magnitude of benefits of evening blue-depleted light environment and future research should consider whether the dose of light exposure and adherence to the condition improves patient outcomes, reduces length of stay, and/or identifies specific patient groups or symptoms that benefit more than others from admission in a blue-depleted light environment.

## Supporting information

S1 File Approved registration and protocol. (PDF)

S2 File Statistical Analytic Plan (SAP). (PDF)

S3 File Section A–H. Section A: Trial registration. Section B: Daylight in recruitment period. Section C: Reporting of the trial. Section D: Study period, Section E: Clinical imperative: withdrawing a participant from the RCT. Section F: Number needed To Treat (NNT). Section G: Study Monitoring. Section H: Additional analysis: extended follow-up of length of stay.
(PDF)

S1 CONSORT Checklist CONSORT 2010 checklist of information to include when reporting a randomised trial. (PDF)

S1 Table Baseline medication assessment. (PDF)

S2 Table Baseline characteristics. (PDF)

S3 Table Observed values for primary and secondary outcomes. (PDF)

S4 Table Subgroup analyses of duration of admission. (PDF)

S5 Table Per protocol analyses of duration of admission (*n* = 349). (PDF)

S6 Table Additional analysis: extended follow-up period for duration of admission (*N* = 476). (PDF)

S1 Fig Side effects. (PDF)

## Abbreviations

BBG: blue blocking glasses
BVC: Broset Violence Checklis
CGI: Clinical Global Impression
CI: confidence interval
DSMC: Data and Safety Monitoring Committee
ICD: International Classifications of Diseases
ipRGC: intrinsically photosensitive retinal ganglion cell
ITT: intent to treat
LED: light emitting diode
NNT: Number Needed to Treat
RCT: randomised controlled trial
SAP: statistical analysis plan
SD: standard deviation
SOAS-R: Staff Observation Aggression Scale-Revised
WHO: World Health Organization
