# Supplementary material for: Clinical benefits of modifying the evening light environment in an acute psychiatric unit: A single-centre, two-arm, parallel-group, pragmatic effectiveness randomised controlled trial
Source: PLoS Med. 2024 Dec 6;21(12):e1004380. doi: 10.1371/journal.pmed.1004380 (PMC11661622; doi:10.1371/journal.pmed.1004380)
Supplement: S1 File — (PDF) [file pmed.1004380.s001.pdf]

S1 Supplementary information. Approved registration and protocol

Project application Form for application for approval of research projects in the Regional Committees for Medical and Health Research Ethics (REK)

2018/946-1
Document ID: 1018880 Document received 07.05.2018

Use of lights in psychiatric hospital buildings to reduce hospitalization time and stabilize sleep-wake rhythm in acute hospitalization. A randomized controlled trial.

1. General information

1.1 Project manager

CRIStin Person ID 36899

Name: Håvard Kallestad Academic degree: PhD

Clinical competence: Specialist in psychology

Position: Position: Researcher

Main workplace: NTNU

Work address: Department of Mental Health Postal code: 7441

Place: Trondheim

Telephone: 93027262

E-mail address: havard.kallestad@ntnu.no

1.2 Project title

Norwegian title: Use of light in psychiatric hospital buildings to reduce hospitalization time and stabilize sleep rhythm in acute hospitalization. A randomized controlled trial.

Academic title: An environmental approach to reducing duration of hospitalization by optimizing circadian rhythmicity in individuals with acute mental disorders. A Randomized controlled trial.

1.3 Research Manager

Institution	Contact person	Position	E-mail address
1. St. Olavs Hospital HF	Pål Sandvik	Manager	pal.sandvik@stolav.no

1.4 Initiator

Who is the initiator of the project?	Project manager and/or institution responsible for research (contributory research)

1.5 Education project

Is the project part of an education or doctoral degree?	Yes
Studies	Medicine and Health
Level	PhD

1.6 Project staff

Name	Position	Institution	Academic role	Project role
1. Knut Langsrud	Head of Section Acute ward	St. Olavs Hospital HF	Cand. Med.	Project participant
2. Gunnar Morken	Professor	Norwegian University of Science and Technology	Phd	Project participant
3. Daniel Vethe	Fellowship	Norwegian University of Science and Technology	Cand. Psychol.	Project participant
4. Janine Linda Scott	Professor	Norwegian University of Science and Technology	Phd	Project participant

1.7 Project timeframe

Project start date	10.09.2018
Project end date	30.06.2038

1.8 Public access

Is an exemption from public access to applications or attachments sought?	No

1.9 Cooperation with foreign countries

Does the project have any form of cooperation with foreign countries?	No

1.10 Other project of significance for the assessment

Is there any other project that may have a bearing on the assessment of the project in question?	Yes

Name of the research project

Development of a therapeutic hospital building

Processed in REK	Yes
After May 5, 2009	
Processed by	REK Midt
Project number in REK	2017/916/REK Midt

2. Project information

2.1 Summary of the research project

Project description

In recent years, new knowledge has emerged about the importance of light and darkness for sleep and mental health. In particular, it has been shown that blue light in the evening is destructive to the circadian rhythm, while blocking blue light in the evening can be a new and effective drug-free treatment for serious mental disorders. In this project we will use a lighting technology where you can adjust the color of the light during the day. Thus, you can create a light environment where there is no blue light in the evening and night, while there is normal light the rest of the day. The main objective is to investigate whether this can reduce the time until the patients' condition improves and whether patients can be discharged to home more quickly. We will investigate this in a study at St. Olavs Hospital, Østmarka, where 500 patients by lot will either be admitted to a ward with a blue-blocked light environment between 1830 and 0700 hours or in an identical ward with normal light around the clock. All other treatment is normal.

2.2 Drug trials

Drug trials:	No

2.3 Research data

**2.3.1 Previously registered information**	Yes

Specify the types of data

Patient records

Regional or local health registry

Which(t)	Patient Administrative System (PAS)

What information is retrieved from the register?

Dates of admission and discharge, diagnoses, admission clause.

Patient records or other treatment-oriented registry

Patient records

Enter which patient records

Pasient records at St. Olavs Hospital

What information is retrieved from patient records?

Information on the patient's level of functioning, symptoms, improvement, side effects, sleep, medication prescriptions, use of coercive mental health care, use of coercive measures, threatening behaviour/risk of violence, cases of physical acting out, diagnosis.

**2.3.2 New health information**	Yes

Specify the types of health information

Patient records, Patient Administrative System (PAS)

**2.3.3 Human biological material**	No

2.4 Study population

2.4.1 Number of research participants and calculation of strength

As there are no other comparable data, we have calculated strength based on being able to show results that will be clinically and operationally relevant. Number of participants and

Statistical power is calculated for the main outcome measure, which is the number of hospitalization days per year. This has been chosen because the length of admission will be an indicator of the time to stabilization of the condition, and in addition because emergency admissions represent the largest cost in mental health care with an estimated daily price of approximately NOK 10000.

There were 1639 admissions to the emergency ward in a 12-month period from May 2016 to April 2017. The average length of stay in this period was 6.3 days (median 4 days, range 0-158 days). This corresponds to > 10,000 bed days per annum. A 10%-15% reduction corresponds to 1000-1500 fewer bed days per annum, which gives a reduction in average hospitalisation time by one day, from 6 to 5 days (median from 4 to 3.5 days). This reduction also means that the hospital will free up 5 extra beds over the course of a year, which will be a significant operating gain.

Given these assumptions, 194 patients in each condition will give us an 80% probability (alpha = 0.05) of detecting a difference of 1 bed day with an Intention To Treat (ITT) analysis, and > an 85% probability of detecting a reduction of 1000 bed days in one year. We calculate a 25% dropout rate from the study and will therefore include 500 patients.

2.4.2 Description of research participants/sample

Patients/Clients

Specify which patients

All patients admitted to an acute ward, St. Olavs Hospital, Division of Mental Health Care, Østmarka Department

Justify the choice of patient group

The intervention is part of the building structure at the acute ward, St Olavs Hospital, Østmarka and the trial is designed to investigate the effect of the intervention on the population admitted there.

Persons with reduced capacity to give consent

Specify which persons have reduced capacity to give consent

The study is conducted in a psychiatric acute ward. Patients admitted to an acute ward may have significant mental symptoms and/or behavioral abnormalities, be in a psychotic and/or manic episode, be acutely suicidal and some have been involuntarily hospitalized.

Justify why these people should be included

The patients with reduced capacity to provide consent will be the most poorly functioning patients. It is very important that new low-intensity treatments are investigated for this patient group so that these patients can also be offered evidence-based treatment.

2.5 Research methods

2.5.1 Method of analyzing data

Statistical (quantitative) analysis methods

2.5.2 Method of data retrieval

Clinical examination

Specify

Data from clinical examinations performed as part of normal clinical practice. This includes the patient's functioning, improvement and side effects (Clinical Global Impression scale), risk of violence (Brøset Violence Checklist), physical acting out (The Staff Observation Aggression Scale – Revised), diagnosis and other information from medical records.

Questionnaire

Observation

Film/video	No
Photographers	No

2.6 Rationale for choice of data and method

Explain the scientific and scientific rationale for the choice of data and method

In order to compare the effect of two therapies, it is assumed that the best method is to use a randomized design. It is not possible to blind patients to the type of treatment in this study. In order to conduct a randomized treatment study involving all patients admitted to an emergency ward, it is necessary that the data collection is not so extensive that it becomes practically difficult to perform, or entails an extra burden for the patients. The medical record data collected in this study are standardized and based on internationally recognized methods. This will provide reliable and valid answers to the research questions in the study while making the study practically feasible.

3. Information, consent and privacy

3.1 Consent will be obtained

Consent will be obtained	No

3.2 Consent has already been obtained

Consent has already been obtained	No

3.3 Exemption from the requirement to obtain consent is sought

Exemption from the requirement to obtain consent is sought Yes

For which participants, information and, if applicable, samples do you apply for exemption from the requirement to obtain consent?

Exemption from the requirement to obtain consent for all patients upon inclusion in the study is sought. Instead, we apply for patients to be given an opportunity to have their data deleted from the research database once the patient's condition has stabilized.

Why is an exemption from the general rule on obtaining consent sought?

We also refer to the previously submitted description of the study «Development of a therapeutic hospital building. A randomized controlled trial" and subsequent meeting with the Secretariat in REK Midt, where we have discussed various ways of handling consent in this study. The procedure described below was developed after consultation with the Secretariat.

In this study, we will include patients who are admitted to an acute psychiatric ward. Several of these patients are in a state where they do not have the capacity to consent, such as manic episodes or psychosis. These patients are also often hospitalized under compulsory hospitalization. This makes it difficult to obtain informed consent for participation in the study on admission, and such a procedure will lead to a bias in the sample of participants. From previous research projects on the emergency ward in Østmarka, we have found that if consent is obtained at intake or in the first days after admission, many patients with psychotic disorders or patients who are involuntarily hospitalized will not consent to participation. The condition of many of these patients will improve up to discharge and after discharge. We apply for exemption from the main rule of obtaining consent upon admission/inclusion and instead apply for approval of a procedure where all patients are included on admission, but where the patients are given an opportunity to withdraw from the study when their condition is stable.

The study involves testing a new way to use a light intervention to improve treatment outcomes for patients in an acute condition. Since admissions in general, and enforced hospitalizations in particular, entail a considerable intervention in the patient's life, measures that can potentially reduce the time spent on stabilization of the condition and length of stay will be of great importance for this patient group and for society. There has been little development of new types of treatment for this patient group, and treatment today is mainly based on pharmacological treatments and shielding. The Government and patient organizations have also demanded that patients admitted for treatment in mental health care be offered drug-free treatment alternatives in addition to ordinary drug treatment. A significant problem in order to meet this requirement is that there is too few evidence-based drug-free interventions to offer patient groups admitted to an acute psychiatric ward, especially for patients with manic and psychotic episodes. In this way, this research project will contribute to meeting the requirement from the Government and the patient organizations for the Health Regions for more patients.

At today's emergency ward, this light intervention has already been installed and is in clinical use in half of the ward. This means that half of all acutely admitted patients today receive this treatment as part of ordinary clinical practice. The emergency ward has been in clinical operation since November 2017 and during this period approximately 900 patients have been admitted, approximately 450 in each type of lighting. We have not observed any adverse effects, and only one patient has wanted to be transferred to beds with traditional lighting. In a pilot study with healthy controls that voluntarily allowed them to be admitted to the emergency ward for 10 days, 5 days in each half of the ward (previously applied for and approved by REK), we also found no side effects. Preliminary experience thus indicates that the risk of adverse events as a result of the intervention is small, and clinical experience shows that patients perceive the lighting as pleasant.

The data collection that we propose in this study is based on data from medical records and data that are already collected as part of ordinary clinical operations. This means that no additional examinations of patients will be carried out beyond current practice. The difference for the patients will be that in this study we want to use an external randomization algorithm (developed by the Department of Clinical Research, NTNU, and outside our control) to randomly allocate the patients to one of the two conditions.

Based on this, we wish to conduct a randomized treatment study in which all patients are included upon admission, without consent. As part of current practice, all patients receive written information about the department upon admission. This booklet contains practical descriptions of visiting hours, meal times, etc. In addition, the booklet contains a description of the new technologies installed (lighting technology and sensor technology). We will also describe that there is an ongoing research project using these technologies in this booklet and that patients can ask their environmental contact to set up a meeting with a person from the research group when the patient's condition is stable. The patient will then receive oral and written information about the research project and have questions answered. In addition, patients who do not wish to participate will sign a form confirming that they wish to have their data excluded from the research database.

Since the proposed procedure deviates from the general rule on obtaining consent prior to inclusion in the study, an international member of the research group (Professor Jan Scott) has sent letters to editors of leading professional journals in psychiatry asking for their assessment of the above issue and procedure. Specifically, given that the Regional Committee for Medical and Health Research Ethics in Norway considers this procedure to be satisfactory:

«Do you think this <procedure> is in keeping with the ethical approach of your journal and/or is sufficient for the study to be considered ethical by an international journal?

Is the option of delayed opt out (rather than written consent to opt in) likely to be an issue if the findings of the study are submitted for consideration for publication in your journal?

Is there anyone else who is linked to your journal that we should approach for advice regarding

this subject?"

The description and questions were sent to editors of the American Journal of Psychiatry, the Lancet Psychiatry, the British Journal of Psychiatry, and the Australia and New Zealand Journal of Psychiatry. The editors have found the issue interesting and the considerations relevant. No one has described misgivings about the procedure. The Lancet Psychiatry has also had the matter discussed in an editorial meeting and they would like to print a letter with us describing the issue and the proposed procedure. See copy of response from editor Joan Marsh below:

Begin forwarded message:

From: "Marsh, Joan (ELS-CAM)" <joan.marsh@lancet.com>

Subject: Ethics query: sleep wards in Norway and delayed opt-out

Date: 12 April 2018 at 14:59:22 BST

To: "jan.scott@newcastle.ac.uk" <jan.scott@newcastle.ac.uk>

Dear Jan,

We discussed your query at today's Lancet editorial meeting. Basically, everyone agreed that the delayed opt-out was acceptable in these circumstances. We have two articles that support this argument:

The Lancet published a letter discussing a similar question with respect to the CRASH trial:

http://www.thelancet.com/journals/lancet/article/PIIS0140-67 36(11)60317-6/fulltext

This concluded:

If consent rituals delay the start of a trial treatment such that the treatment effect could be reduced or obscured, we maintain that seeking consent is actually unethical. There might be other treatments whose benefits have been missed or underestimated as a result of insistence on the rituals of informed consent, against the precepts of the Declaration of Helsinki, with resultant avoidable harm to patients.

The debate was also covered in The Lancet in 2014 regarding HEAT PPCI.

One person asked if you could obtain permission from next-of-kin but we realise this probably isn't feasible for this adult population.

We'd be interested to know what the other journals said: could you let me know, once you have all the feedback? As a learning point, I'd like to publish a letter about this in European Science Editing, an editors' journal: perhaps we could co-author one?

Regards,

Joan

4. Balancing the benefits and risks of the project

4.1 Advantages

Indicate physical, mental, social and/or practical benefit/benefit now or in the future for the individual patient/participant, groups of persons, society and/or science.

If the new intervention is effective, patients who are randomly assigned to the intervention group will have less time to recovery, sleep can be improved and consumption of sedative medications can be reduced, but we will not know until the study is finished.

New interventions are rarely developed for this group of patients, and even less frequently new interventions are tested in large randomised treatment trials in representative samples. This may have a major impact on the patient group admitted to an acute psychiatric ward.

This is the first study of its kind in the world and the scientific quality of the experiment means that the results could potentially be of great scientific interest. The experiment may lead to new knowledge about how serious mental disorders are maintained and acutely treated. The results of this study may also influence how future hospital wards are created.

4.2 Disadvantages

Indicate physical, mental, social and/or practical risk/injury/discomfort/strain/inconvenience now or in

the future of the individual patient/participant, groups of persons, society and/or environment.

To date, we have not identified any disadvantages of the intervention. We have investigated this in a pilot study and as part of 6 months of clinical operation. It thus appears as a Low-intensity treatment without obvious side effects, but we cannot rule out that there may be some who may have side effects from the lighting.

4.3 Measures

Describe any special measures to safeguard and protect the patients/participants in the research project and to limit possible risk/disadvantage

All patients will receive the treatment they would normally be entitled to and as their condition warrants. This entails the highest level of preparedness in mental health care: Awake staff are present at all times and patients are supervised at least every 30 minutes, 24 hours a day. For emergency situations, there are emergency switches that immediately turn all lights in the patient's room and/or in the rest of the ward to normal white hospital lighting. If patients experience obvious side effects of the intervention, it will be possible to set the lighting in the patient's room permanently to normal light.

4.4 Prudence

Why is it justifiable to carry out the project? Provide a reasoned balance between the advantages and disadvantages of the research project.

If the intervention tested here can lead to faster recovery and discharge, this will have future benefits for this patient group, and the trial may be of scientific and social interest. Since the study does not entail any changes to the emergency ward beyond clinical practice, the patient's welfare and integrity will be safeguarded. We believe it is necessary to conduct the study without consent at inclusion to ensure that the results can be generalizable to the patient population that will receive the treatment in the future, but that all patients have a delayed opportunity to withdraw from the study. This solution is proposed by REK and subsequently also assessed by editors of important medical journals.

5. Security, interests and publication

5.1 Personally identifiable information

In what form will personally identifiable information and samples be used in the project?

De-identified with link key

Provide information about how the link key is stored and who has access to it

All information will be retrieved from the patient record and transferred to a data file that has been de-identified. The link key will be stored on paper in a locked cabinet and digitally on an encrypted storage device as recommended by the Vice Dean for Research at the Faculty of Medicine and Health.

5.2 Internal control and security

Your institution's server

Password-protected retention

5.3 Insurance for research participants

The Patient Injury Act (Pasientskadeloven)

5.4 Assessment of other agencies

The project has been assessed/will be assessed by:

Own institution

5.5 Interests

Funding sources

St. Olavs Hospital, Division of Mental Health Care is funding the study as part of normal operations. PhD candidates working on the study are funded by: NTNU (two PhD candidates), Extrastiftelsen / Council for Mental Health and the Norwegian Research Council. St. Olavs Hospital funds research nurses.

Remuneration to institution

None

Remuneration project manager/employees

None

Compensation for research participants

None

Any conflicts of interest for project managers/employees

None

5.6 Publication

Are there restrictions with regard to publication and publication of the results of the project?	No

Explain how the results will be made publicly available

We will publish the results of the study in international peer-reviewed journals, scientific conferences/congresses and popular science lectures. As part of the publication, we plan to collaborate with the media department of St. Olavs Hospital and the journal Gemini to make the results known in Norwegian/international media. The user group at St. Olavs Hospital and the Council for Mental Health will disseminate information through their networks.

5.7 Handling of data after project completion

How will personal data be handled after the end of the project?

The personal data in the patient's records will remain there in accordance with current rules for St. Olavs Hospital and data storage of medical records.

6. Appendix

#	Type	Filename	Posted date
1.	Information booklet given to all patients upon admission	Information leaflet patients.docx	07.05.18
2.	Questionnaire	Patient satisfaction.pdf	07.05.18
3.	SOAS-RE, please note that the form's graphic design is different in clinical use	SOAS-RE.pdf	07.05.18
4.	BVC, please note that form's graphical design is different in clinical use	BVC.pdf	07.05.18
5.	CGI, please note that	CGI table English and Norwegian.pdf	07.05.18

	the graphical design of the form is different in clinical use		
6.	Research protocol	Protocol_REK_AcuteStudy.docx	06.05.18
7.	Information about the study and reservation from participation	Informasjon_reservasjon.docx	06.05.18
8.	CV for the project manager	CV_Kallestad_2018.docx	06.05.18

7. Legal Notice

I declare that the project will be implemented
in accordance with applicable laws, regulations and guidelines
in accordance with information provided in this application;
in accordance with any conditions for approval granted by REK or other agencies

1. An environmental approach to reducing duration of hospitalization by optimizing circadian rhythmicity in individuals with acute mental disorders. A Randomized controlled trial.

Overview of the project:
Individuals with mental disorders are admitted to acute psychiatric inpatient units for several reasons: acute distress associated with risk of suicide, significant increase in illness severity, or major impairment in day-to-day functioning arising from mental or behavioral dysregulation. Although the quality and fabric of inpatient facilities have improved over recent decades with more attention to providing a homely environment and better quality accommodation, little attention has been given to how to use the environment of the unit to optimize treatment and improve the mental state and social functioning of the patients. In general, there has been a lack of innovation in treatments facilities for acutely admitted patients in mental health care over the last 50 years. This project specifically focuses on the development of an innovative program to enhance the environment within an acute inpatient unit to try to reduce time to stabilization of mental state, and to enable patients to achieve earlier discharge back to their home and community.

This project is based on two important empirical findings. First, poor sleep and circadian dysrhythmia are highly prevalent and significantly associated with relapse or worsening of major mental disorders (e.g. psychotic, bipolar and major depressive disorders) and with increased suicidality; whilst improvement in sleep and circadian rhythmicity are associated with earlier improvement in mental state. Thus, an important target for all acute psychiatric treatment is stabilization of the sleep-wake cycle. Second, recent findings from basic physiology and treatment research has elucidated how light at specific frequencies can have a profound impact on arousal, sleep, and circadian disruption. Moreover, blocking these specific light frequencies can be a highly effective treatment for severe mental disorders, leading to early improvement in symptoms and functioning. This is a potential breakthrough in our understanding of how severe mental disorders are maintained, and offers the opportunity for the development of innovative non-pharmacological interventions.

Based on these findings, we have incorporated new light technology into a newly-built psychiatric inpatient unit to create a hospital environment and infrastructure that we hypothesize will: promote sleep-wake cycles and circadian stability, and lead to a shortening of the average length of admission. Also, it may allow a reduction in the number or dosages of medications used to achieve improvements in mental state and functioning. This is a new approach and there is a critical need to evaluate all aspects of the approach to determine the benefits and possible drawbacks or adverse effects.

Aim: To test the effects of this new hospital environment in a randomized controlled treatment trial of 500 in-patients admitted to the new hospital. The trial will test the effects on duration of admissions, level of functioning, risk of aggressive behavior, use of medication, sleep, and potential side-effects, of this environment, compared to patients admitted to an inpatient environment that lacks the new lighting technology.

Potential impact: Findings from this study could improve our understanding of relapse and remission in severe mental disorders, the importance of the sleep-wake cycle in health and disease, and could impact the way we design future hospital units (for physical as well as mental disorders).

2. Background

The circadian system: Almost all living organisms display some form of synchronicity with the light-dark cycle of the sun, circadian rhythms. In humans, circadian rhythms are orchestrated from the main biological clock in the *suprachiasmatic nuclei* (SCN) in the hypothalamus, and are responsible for a range of physiological and behavioral changes related to sleep and wakefulness.[1,2] When in synchronicity, this circadian system allows us to be vigilant and awake during the day and it promotes de-arousal and sleep during the night. However, disruptions to the circadian rhythms can affect our mental and somatic health, and can result in poor cognitive performance, depression, insomnia, metabolic abnormalities, obesity, immune impairment, and a greater risk of cancer.[1] This is particularly relevant for patients with mental disorders who frequently display some kind of sleep-wake disruption.[3,4] New evidence from our research group, using data from the acute ward at Østmarka, has shown that large day-to-day variability in the sleep-wake cycle is associated with longer duration of admission and higher levels of aggressive behavior and violent incidents.[5,6] For decades it has been assumed that this sleep-wake disruption was a secondary symptom of a primary mental disorder, but recently this has changed. Experimental and clinical data now suggest a reciprocal relationship between sleep-wake disruption and mental disorders where they perpetuate and aggravate each other, whilst improved sleep is associated with improvement in mental state.[7-10] This makes stabilization of the sleep-wake cycle and circadian rhythm an important therapeutic target.[6]

Effects of light on humans: Light is the most important *zeitgeber* of the circadian system. More than ever, we are exposed to artificial light sources at night. This provides opportunities to being productive, but artificial light at night also delay circadian rhythms and increase alertness.[11,12] Exposure to light emitting electronic devices at night for four consecutive days may disrupt the circadian rhythms by suppressing melatonin secretion by 50% and delaying melatonin onset by 1.5 hours.[11] Such exposure also reduce the amount of REM sleep and reduce alertness in the morning.[11,13] On the other hand, removing artificial light at night and keeping participants in darkness after sun-set for three days, will advance the timing of melatonin onset and sleep phase.[14] Thus, exposure to artificial light at night may have a large negative impact on the human circadian system, while exposure to darkness at night may have the opposite effect.

Non-image forming light perception: The discovery of a new type of receptor cell in the retina which specifically reacts to light of certain wavelengths in the blue spectrum (wavelengths < 530 nano meters, nm) has led to an increased understanding of how light affects the human circadian system.[15] These receptor cells, called *intrinsically photosensitive retinal ganglion cells* (ipRGC), use melanopsin (OPN4) as their intrinsic photopigment.[16] They do not provide visual information, but send projections to the SCN and are the key input to our circadian system.[1] Interestingly, recent studies have also found that light can have a direct impact on mood through connections from the ipRGC to the limbic system, striatum and the brain stem.[2] This research on the ipRGC has given rise to a way of creating a "virtual darkness"-condition through specifically blocking the light frequencies to which the *ipRGC* responds. Studies have shown that wearing blue-blocking glasses which filters blue light < 530 nm can have a similar effect as total darkness on melatonin onset and counteract the effects of light exposure at night.[17,18]

From basic science to treatment: In a novel treatment trial, Henriksen et al. demonstrated that blue-blocking glasses at night could be used as acute treatment for mania.[19] Using blue-blocking glasses between 18:00 hours and 08:00 hours for seven days led to large improvement in symptoms for patients with a manic episode.[19] Blue-blocking glasses at night have also been found to advance the circadian rhythm of patients with delayed sleep phase syndrome,[20] and could stabilize sleep patterns in mania.[21] Presumably, the effect is caused by imposing a very regular light/dark rhythm by silencing the ipRGC at night, leading to a stabilization of the circadian rhythm, and decreasing

arousal at night. However, Henriksen and colleagues soon experienced that some patients could not make use of the blue-blocking glasses because they had periods of agitation or confusion.

Technological possibilities: New light technology has made it possible to create indoor lighting with highly specific light spectrum. Specifically, it is possible to program light sources to not include light with frequencies < 530 nm at night. This may counteract the negative effects of light at night by avoiding stimulation of the ipRGC and allow the creation of living spaces and hospitals which promotes sleep, stabilizes the circadian system, and decreases arousal at night. Previous research on shiftwork has shown that controlling ambient light at night with regards to blue light content preserves endogenous melatonin similarly to blue blocking glasses.[22]

The new ward at Østmarka acute psychiatric department: Using this technology we have been able to construct a new psychiatric ward at St. Olavs Hospital, Østmarka where all light sources are blocked for blue light at night (between 19:00h and 07:30h), while providing normal light in the rest of the day. This allows us to create a physical environment in the hospital where all patients can receive a non-pharmacological treatment as an integrated part of being in the hospital. This is a new way of offering treatment which has never previously been attempted in a hospital, and the effects and potential side-effects needs to be assessed and tested in a randomized controlled clinical trial.

Before the ward opened for admission, we conducted a randomized cross-over trial with 12 healthy participants as a proof-of-concept trial (see earlier REK application: 2017/916/REK midt). Preliminary analyses have shown that we found a significant and relevant effect on our primary outcome of melatonin secretion, with participants having 15% melatonin suppression in the blue-depleted unit compared to 45% suppression in the normal hospital unit ($p < 0.001$). The timing of the dim light melatonin onset was also advanced with 1:10 hours after the blue-depleted condition compared to the normal hospital light condition ($p = 0.007$). This is suggests that the light intervention has a similar effect on the circadian system that has been found in previous studies using blue-blocking glasses. Please note that these preliminary data from the trial is not published and has not been through peer-review.

Benefit for patients and society: The treatment is less invasive compared to other treatment options, and is likely to have far less side effects than medication commonly used for acute stabilization of mental disorders. We have conducted a pilot trial where we did not find negative effects of the light changes – either for participants or for personnel. So far, approximately 450 patients have been admitted to the blue-depleted half of the ward as part of ordinary clinical practice. We have not been able to discover any unwanted effects of the light and we have not been able to see any negative answers in the Patient Satisfaction Questionnaire that patients complete before discharge from the unit. The clinical impression is that patients experience the blue depleted light as pleasant. If this project can demonstrate shorter time to recovery and discharge from the unit, less aggression and use of mechanical restrictions, and less use of medication in the intervention group, this will have a clear benefit for the patients and staff, particularly patients who are involuntary admitted.

The Minister of Health and Care Services sent a letter to the Norwegian Hospitals, dated Nov 25th 2015, where it was stated that "Patients in mental health care shall, as far as possible, be able to choose non-pharmacological treatments". This need for non-pharmacological treatment options have been echoed by patient user groups. However, for patients who are admitted to psychiatric units for acute treatment, particularly for manic or psychotic episodes, very few evidence-based non-pharmacological treatment options exist and almost all patients are treated with pharmacological treatment options.

New methods of treatment are seldom developed for inpatient settings in psychiatry, and they are almost never tested in large randomized controlled trials. As such the project can potentially have a

high benefit for patients and society. New hospital units are currently being planned at several Norwegian psychiatric hospitals. To have data to either support or rebuke the effectiveness of such an intervention will be important for decision makers in mental health care when planning new hospitals.

The major cost of the new light technology is the installation. After installation, any number of patients can potentially be treated at no additional cost with no added resources beyond ordinary staff at the ward. The primary outcome of the project is to test if the new light technology can reduce duration of admissions with 10%-15% per annum, which would allow improved efficiency.

3. Aims and goals

In a randomized controlled trial, we will test if patients who are admitted to an acute psychiatric hospital unit where lights are blue-depleted between 18:30h and 07:30h have improved clinical outcomes compared to patients who are admitted to an identical unit with normal lights.

Specifically, the main hypotheses for the trial are that, compared to patients admitted to the normal light condition, patients admitted to the blue-depleted unit will have:

1. Shorter time to improved function
2. Shorter duration of hospital admission
3. Improved sleep-wake cycle and circadian stability
4. Less use of sedative medications
5. Less aggressive behavior and incidents.
6. Less use of physical restraints

The secondary aim is to explore potential side effects and test if there are differential outcomes across different mental disorders or other background variables.

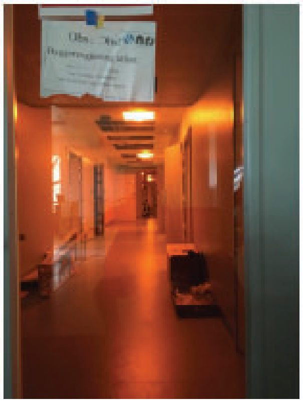

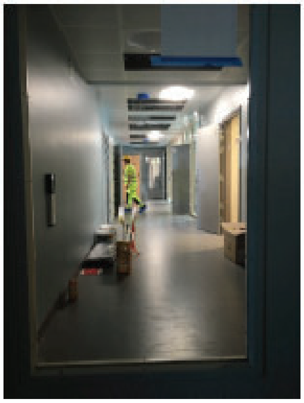

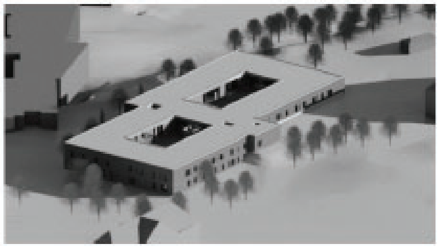

Figure 1 (left) *shows the blue-depleted light condition.* ***Figure 2 (middle)*** *shows the ordinary light condition.* ***Figure 3 (right)*** *shows an overview of the new hospital ward at St. Olavs Hospital, Østmarka. The blue-depleted lights will be active between 18:30h and 07:30h and installed in 20 patient rooms and common areas around the top atrium, ordinary light is installed in the 20 patient rooms and common areas around the lower atrium. The pictures have been taken during the construction phase before the hospital is ready for use.*

Study design, methods and analyses

Setting

A new acute psychiatric unit has been built at St. Olavs Hospital, Østmarka. The unit has 40 patient rooms around two atriums. A programmable light technology has been installed in half the ward, while the other half has ordinary light. See figures 1-3. In the 20 rooms with programmable light, there are also blue-blocking screens in the windows which will be activated at the same time as the blue-depleted lighting to ensure that incident daylight is also filtered for blue light. Electronic media devices (smart phones, TVs, etc) will have filters in front of the screens that have been tested by the research group and does not transmit any blue light. During the day, the light in the two wards will be identical (color temperature 3000K).

Patients

Individuals with mental disorders are admitted to acute inpatient units for three main reasons: acute distress associated with risk of suicide, significant increase in illness severity, or major impairment in day-to-day functioning arising from mental or behavioral dysregulation. The main goals of admission are to stabilize the condition and to observe and diagnose the condition. Median duration of admissions is 4 days, and the range is 0 to 153 days (data from 2016).

Research design

A randomized parallel-group treatment trial where patients admitted to the acute ward will be randomized 1:1 to the different 'light environments' (i.e. an area with normal lighting vs an area with blue-depleted lighting at night).

Randomization will take place at intake. Patients will be randomized using the web-based randomization program developed by NTNU Unit for Clinical Research (undertaken by the admitting psychiatrist following appropriate instruction and training). In situations with no vacant rooms in one of the two parts of the ward, the patient will not be randomly allocated. After the trial period, we will analyze the results for patients who have been randomized and for patients who have not been randomized.

Assessments

All assessments are part of the standard clinical assessment of patients at the acute ward and will not burden the patients beyond what is today's clinical standard.

<u>Duration of admissions:</u> The primary outcome measure will be differences in duration of admission (estimated as total patient bed-days used per annum and as mean duration of admission per individual). The date and time of admission and discharge will be extracted from the electronic records used routinely for all patients attending the hospital.

<u>Level of functioning:</u> The Clinical Global Impression (CGI) will be scored for each patient, every morning in consensus meetings with the hospital staff. The CGI has been extensively used in clinical research previously.[23]

<u>Aggressive incidents:</u> Aggressive incidents are routinely recorded using the Staff Observation Aggression Scale – Revised (SOAS-R) and the Brøset Violence Checklist (BVC). Aggression have previously been shown to be associated with sleep disturbance for patients admitted to the psychiatric acute unit.[5]

<u>Use of physical restraint:</u> All use of physical restraint is documented in hospital medical records and we will be used for analyses.

Involuntary admission: The type of admission at intake (voluntary or involuntary) and duration of involuntary admission before changed to voluntary admission will be collected from the hospital patient registry.

Use of medication: Use of sedative medication will be determined from the medical prescribing records and the data recorded by nurses throughout the admission.

Sleep: Sleep and activity patterns for each individual will be assessed using the Xethru sensor. The Xethru sensor is installed in patient rooms and will be used to assess sleep all nights in the hospital. This is a low-powered ultra-wideband radar that sends a sequence of radio-wave pulses and measures the time it takes for each pulse to be reflected. Using pulse doppler analyses, the sensor measure distance and relative movements less than 1mm with a high degree of precision. Using this data, respiration can be detected by a contact-free sensor. This is a new technology which allows contact-free assessment of sleep/wakefulness with 92% accuracy compared to polysomnographic (PSG) recordings (Pallesen et al., *in press*).

Primary Diagnosis: Diagnoses are set based on the research criteria in the International Classification of Disorders – 10th ed.[24] in diagnostic meetings with the clinicians at the ward where there is always at least two specialists of psychiatry or clinical psychology present.

Patient satisfaction: Will be assessed using the standard patient satisfaction questionnaire upon discharge from the hospital. This is part of ordinary routine at the ward.

Power calculation and sample size

The power calculation and sample size were estimated for the primary outcome measure- namely patient bed-days used per annum and duration of admission. This outcome was chosen as hospitalization represents the major cost of psychiatric care and treatment. For example, the cost of patient care for one day in an acute psychiatric ward is approximately NOK 10.000. The cost of the installation of the light system is approximately NOK 5.000.000 (=USD 590.000). As such, a 10-15% reduction in the inpatient bed-days used per annum and/or a one day reduction in the average length of an acute admission, would both subsidize the entire cost of the lighting system and allow significant additional savings in expenditure on inpatient services that could be re-invested in other aspects of care and treatment.

Data from the hospital system showed that there were 1639 acute admissions during the last 12 months (May 2016 to April 2017), with a mean length of stay of 6.3 days (median 4 days; range: 1 to 158 days). This equates to >10,000 patient bed-days per annum. A 10-15% reduction represents 1000-1500 fewer patient bed-days per annum, with a reduction in the mean length of stay from about 6 to 5 days (and a reduction in the median length of stay from 4 to about 3.5 days). Given the above assumptions a sample of 194 participants in each condition will give an 80% chance (at an alpha = 0.05) to detect a difference of 1 day using an intent to treat (ITT) analysis and >85% power to detect a reduction of 1000 patient bed-days used over the course of the year. As there is no previous study on which to base calculations of early dropouts or attrition rates, we have assumed a 25% dropout-attrition rate, hence we will recruit 500 participants to the RCT.

4.2 Organization and infrastructure

The only difference to ordinary clinical practice in this trial will be to include a randomization procedure in order to allocate patients to the different conditions rather than having the doctor at intake allocating the patients to an available room. However, running a treatment trial in an acute ward is a demanding task, and four PhD students (all licensed medical doctors or clinical psychologists) will work on the daily operations of the trial in addition to one research nurse. The

trial is supported by the head of the division of mental health care (Liv Sjøvold), the head of department (Pål Sandvik) and the head of the acute wards (Knut Langsrud).

Interdisciplinary cooperation: This project involves cooperation between the following departments at NTNU: Mental Health and Neuroscience. In addition, it involves cooperation between two clinics at St. Olavs University Hospital: Mental Health and Clinical Neurophysiology.

The research group:

- *Håvard Kallestad*, PhD, specialist of clinical psychology at St. Olavs University Hospital and researcher at NTNU Department of Mental Health. Kallestad is the PI of the current project.
- *Knut Langsrud*, MD, psychiatrist and head of the acute wards at St. Olavs University Hospital, Østmarka.
- *Terje Torgersen,* MD, PhD, psychiatrist at St. Olavs Hospital and head of research at the acute ward, St. Olavs Hospital.
- *Gunnar Morken*, MD, PhD, is professor of psychiatry and director of research, innovation and education at St Olavs University Hospital and Faculty of Medicine and Health Sciences, NTNU, and psychiatrist at the acute wards at St. Olavs University Hospital, Østmarka. Professor Morken will along with Dr. Kallestad, Dr. Langsrud, and Dr. Torgersen oversee the current project.
- *Trond Sand,* MD, PhD, is professor oat the Department of Neuromedicine and Movement Science, NTNU and head of the Department of Clinical Neurophysiology at St. Olavs Hospital.
- PhD candidates working on the project and responsible for daily operations.
  - *Daniel Vethe,* cand. psychol, is a clinical psychologist at the sleep clinic at St. Olavs Hospital department of Østmarka, and PhD candidate at NTNU Department of Mental Health.
  - *Cecilie Lund Vestergaard,* MD, is a PhD candidate at NTNU Department of Mental Health.
  - *Kaia Kjøstad,* cand. psychol. is a PhD candidate at NTNU Department of Mental Health.
  - *Sissel Marguerite Belanger,* cand. psychol, is a PhD candidate at NTNU Department of Mental Health.
- *Barbara Matusiak*, PhD, is professor of Architecture and head of the Daylight Lab at Department of Architecture and Technology. Professor Matusiak has tested all light sources and filters that will be used in the current project and her knowledge about how light and architecture it affects human experience is central to this project.
- *Morten Engstrøm*, MD, PhD, is associate professor at the Department of Neuromedicine and Movement Science, NTNU and a clinical neurophysiologist at the Department of Clinical Neurophysiology at St. Olavs University Hospital. Dr. Engstrøm is an expert on the physiology of sleep and is the chair of the Norwegian Sleep Research Society. He leads a parallel research project on the Xethru sensor and will be involved in scoring and interpretation of the sleep data in the current study.
- *Jan Scott*, PhD, FRCPsych is a professor and internationally acclaimed researcher in mood disorders at the University of Newcastle, UK and undertakes research on activity and sleep-wake patterns in health and disease. She is a leading researcher on non-pharmacological and adjunctive interventions for mental disorders (including studies of cost-effectiveness and health economics) and has published extensively in high impact journals. She has cooperated on the study design and will help in the interpretation of the data analyses and assist in drafting manuscripts arising from the project.

5. User participation

The leader of the User Group in Mental Health, St. Olavs Hospital, Hege Hafstad Johansen, support the project. The User Group, represented by Hege Hafstad Johansen and Bjørn Einar Moe, have been involved in all processes related to research at the new ward from the start and has had several meetings with the research group where study designs have been discussed.

6. References

1. Foster RG, Wulff K. The rhythm of rest and excess. *Nature reviews Neuroscience.* 2005;6(5):407-414.
2. LeGates TA, Fernandez DC, Hattar S. Light as a central modulator of circadian rhythms, sleep and affect. *Nature reviews Neuroscience.* 2014;15(7):443-454.
3. Wulff K, Gatti S, Wettstein JG, Foster RG. Sleep and circadian rhythm disruption in psychiatric and neurodegenerative disease. *Nature reviews Neuroscience.* 2010;11(8):589-599.
4. Kallestad H, Hansen B, Langsrud K, et al. Impact of sleep disturbance on patients in treatment for mental disorders. *BMC Psychiatry.* 2012;12:179.
5. Langsrud K, Kallestad H, Vaaler A, Almvik R, Palmstierna T, Morken G. Sleep at night and association to aggressive behaviour; Patients in a Psychiatric Intensive Care Unit. *Psychiatry Res.* 2018;263:275-279.
6. Langsrud K, Vaaler AE, Kallestad H, Morken G. Sleep patterns as a predictor for length of stay in a psychiatric intensive care unit. *Psychiatry Res.* 2016;237:252-256.
7. Goldstein AN, Walker MP. The role of sleep in emotional brain function. *Annu Rev Clin Psychol.* 2014;10:679-708.
8. Manber R, Edinger JD, Gress JL, San Pedro-Salcedo MG, Kuo TF, Kalista T. Cognitive behavioral therapy for insomnia enhances depression outcome in patients with comorbid major depressive disorder and insomnia. *Sleep.* 2008;31(4):489-495.
9. Harvey AG, Soehner AM, Kaplan KA, et al. Treating insomnia improves mood state, sleep, and functioning in bipolar disorder: a pilot randomized controlled trial. *J Consult Clin Psychol.* 2015;83(3):564-577.
10. Perlis ML, Grandner MA, Brown GK, et al. Nocturnal Wakefulness as a Previously Unrecognized Risk Factor for Suicide. *J Clin Psychiatry.* 2016;77(6):e726-733.
11. Chang AM, Aeschbach D, Duffy JF, Czeisler CA. Evening use of light-emitting eReaders negatively affects sleep, circadian timing, and next-morning alertness. *Proc Natl Acad Sci U S A.* 2015;112(4):1232-1237.
12. Cho Y, Ryu SH, Lee BR, Kim KH, Lee E, Choi J. Effects of artificial light at night on human health: A literature review of observational and experimental studies applied to exposure assessment. *Chronobiol Int.* 2015;32(9):1294-1310.
13. Cho CH, Lee HJ, Yoon HK, et al. Exposure to dim artificial light at night increases REM sleep and awakenings in humans. *Chronobiol Int.* 2016;33(1):117-123.
14. Stothard ER, McHill AW, Depner CM, et al. Circadian Entrainment to the Natural Light-Dark Cycle across Seasons and the Weekend. *Curr Biol.* 2017;27(4):508-513.
15. Brainard GC, Hanifin JP, Greeson JM, et al. Action spectrum for melatonin regulation in humans: evidence for a novel circadian photoreceptor. *The Journal of neuroscience : the official journal of the Society for Neuroscience.* 2001;21(16):6405-6412.
16. Panda S, Provencio I, Tu DC, et al. Melanopsin is required for non-image-forming photic responses in blind mice. *Science.* 2003;301(5632):525-527.
17. Sasseville A, Paquet N, Sevigny J, Hebert M. Blue blocker glasses impede the capacity of bright light to suppress melatonin production. *J Pineal Res.* 2006;41(1):73-78.
18. van der Lely S, Frey S, Garbazza C, et al. Blue blocker glasses as a countermeasure for alerting effects of evening light-emitting diode screen exposure in male teenagers. *J Adolesc Health.* 2015;56(1):113-119.
19. Henriksen TE, Skrede S, Fasmer OB, et al. Blue-blocking glasses as additive treatment for mania: a randomized placebo-controlled trial. *Bipolar Disord.* 2016;18(3):221-232.
20. Esaki Y, Kitajima T, Ito Y, et al. Wearing blue light-blocking glasses in the evening advances circadian rhythms in the patients with delayed sleep phase disorder: An open-label trial. *Chronobiol Int.* 2016;33(8):1037-1044.
21. Henriksen TE, Skrede S, Fasmer OB, Hamre B, Gronli J, Lund A. Blocking blue light during mania - markedly increased regularity of sleep and rapid improvement of symptoms: a case report. *Bipolar Disord.* 2014;16(8):894-898.
22. van de Werken M, Gimenez MC, de Vries B, Beersma DG, Gordijn MC. Short-wavelength attenuated polychromatic white light during work at night: limited melatonin suppression without substantial decline of alertness. *Chronobiol Int.* 2013;30(7):843-854.
23. Busner J, Targum SD. The clinical global impressions scale: applying a research tool in clinical practice. *Psychiatry (Edgmont).* 2007;4(7):28-37.
24. WHO. *The ICD-10 Classification of Mental and Behavioural Disorders, Clinical Description and Diagnostic Guidelines.* Oslo: Gyldendal Akademisk Forlag; 1992.

Scott et al. Trials (2019) 20:472
https://doi.org/10.1186/s13063-019-3582-2

Trials

STUDY PROTOCOL Open Access

A pragmatic effectiveness randomized controlled trial of the duration of psychiatric hospitalization in a trans-diagnostic sample of patients with acute mental illness admitted to a ward with either blue-depleted evening lighting or normal lighting conditions

Jan Scott[1,2], Knut Langsrud[1,3], Daniel Vethe[1,3], Kaia Kjørstad[1,3], Cecilie L. Vestergaard[1,3], Patrick Faaland[1,3], Stian Lydersen[1], Arne Vaaler[3], Gunnar Morken[1,3], Terje Torgersen[3] and Håvard Kallestad[1,3,4*]

Abstract

Background: There is increasing recognition of the need to stabilize sleep-wake cycles in individuals with major mental disorders. As such, clinicians and researchers advocate the use of interventions targeted at sleep and circadian dysrhythmias as an adjunct to the standard treatments offered for acute illness episodes of a broad range of diagnoses. To determine the trans-diagnostic generalizability of chronotherapy, we explore the benefits of admitting individuals with an acute illness episode to a psychiatric inpatient unit where changes in light exposure are integrated into the therapeutic environment.

Methods/design: A two-arm, pragmatic effectiveness, randomized controlled treatment trial, where individuals admitted for acute inpatient psychiatric care will be allocated to a ward with blue-depleted evening light or to a ward with the same layout and facilities but lacking the new lighting technology. The trial will test whether the experimental lighting conditions offer any additional benefits beyond those associated with usual treatment in an acute psychiatric inpatient unit. The main objectives are to examine any differences between groups in the mean duration of hospitalization in days. Additional analyses will compare group differences in symptoms, functioning, medication usage, and side effects and whether length of stay is associated with stability of sleep-wake cycles and circadian rhythms. Ancillary investigations should determine any benefits according to diagnostic subgroups and potential drawbacks such as any adverse effects on the well-being of professionals working across both wards.

(Continued on next page)

* Correspondence: havard.kallestad@ntnu.no
[1]Department of Mental Health, Norwegian University of Science and Technology, Trondheim, Norway
[3]Division of Mental Health Care, St. Olavs University Hospital, Trondheim, Norway
Full list of author information is available at the end of the article

© The Author(s). 2019 **Open Access** This article is distributed under the terms of the Creative Commons Attribution 4.0 International License (http://creativecommons.org/licenses/by/4.0/), which permits unrestricted use, distribution, and reproduction in any medium, provided you give appropriate credit to the original author(s) and the source, provide a link to the Creative Commons license, and indicate if changes were made. The Creative Commons Public Domain Dedication waiver (http://creativecommons.org/publicdomain/zero/1.0/) applies to the data made available in this article, unless otherwise stated.

(Continued from previous page)

Discussion: This unit offers a unique opportunity to explore how exposure to different lighting conditions may modify sleep-wake cycles and how any changes in sleep-wake cycle may impact on the clinical and functional outcomes of individuals experiencing an acute episode of a severe mental disorder that requires inpatient care. The findings could influence the future design of hospital units offering care to patients with mental or physical disorders.

Trial registration: ClinicalTrials.gov, ID: NCT03788993. Retrospectively registered on 28 December 2018.

Keywords: Mental disorders, Acute treatment, Inpatient, Chronotherapy, Light, Blue-depleted light, Admission, Sleep, Circadian rhythms,

Background

Recent decades have seen increased attention to the impact of disturbed sleep on general health [1]. For example, sleep-wake cycle abnormalities linked with circadian dysrhythmias are associated with physical disorders such as diabetes, obesity, and a greater risk of cancer, etc. [2]. As light is a central *zeitgeber* of the circadian system, some researchers have explored the benefits of phototherapy or chronotherapy for selected medical illnesses, especially in those individuals who have a concurrent comorbid mental disorder [3]. The latter is noteworthy as sleep problems are uniquely important in the field of mental health [4]. For instance, sleep abnormalities may be prodromal symptoms heralding the onset of a major mental disorder, sleep-wake cycle disruptions are criterion symptoms for diagnosing unipolar and bipolar disorders and circadian dysrhythmias may exacerbate suicidal behaviors [5]. Evidence from our research demonstrates that day-to-day variability in sleep-wake cycles is associated with longer duration of acute psychiatric admissions and frequency of aggression or violent incidents [6, 7]. Lastly, sleep problems are often the last symptoms to resolve during recovery from an acute episode of a mood or psychotic disorder [8, 9]. Overall, experimental and clinical research emphasize the reciprocal relationship between sleep-wake disruptions and mental disorders showing that they perpetuate and exacerbate each other and that improved sleep is associated with improvements in mental state [10–13].

The observations noted have increased awareness of the need to stabilize sleep-wake cycles in individuals with major mental disorders and highlighted the importance of providing interventions targeted at circadian dysrhythmias as an adjunct to other treatments for acute illness episodes [14]. Psychological and pharmacological interventions are efficacious approaches for sleep-wake cycle disturbances in adults without comorbid mental disorders [15–17]. However, their use in individuals with an acute exacerbation of a major mental illness can be problematic, including attenuation of the benefit-to-risk ratio for therapies or contra-indications to the use of some medications [15–17]. Partly as a response to these concerns, but also because of new research on circadian rhythms, attention has shifted to the potential role of interventions based on controlled exposures to environmental stimuli that act on biological rhythms [18]. These strategies initially focused on, e.g., morning bright-light therapy for seasonal affective disorders, sleep-wake disorders, and certain sub-types of depression; but there has been an increasing recognition of the importance of darkness at night to improve sleep-wake disorders and affective disorders.

Dark therapy is a treatment where the patient is in darkness an extended period (e.g., between 1800 h to 0800 h), and was initially described in two case-studies [19, 20] before it was tested in a clinical trial with bipolar patients in a manic episode [21]. Although the results were promising, clinical use has been limited as the patient have to be in complete darkness for about 12–14 h. Because the circadian effect of light on humans is primarily mediated by the intrinsically photoreceptive retinal ganglion cells (ipRGC) that have peak sensitivity to blue light [22], it was hypothesized [23], and later shown, that a clinical effect can also be achieved by specifically blocking the blue part of the light spectrum using blue-blocking glasses, rather than being in darkness [24, 25]. No severe side effects were reported, but two manic patients had emerging depressive symptoms that were diminished in less than 1 day [25]. Other trials have also used blue-blocking glasses at night as a treatment for sleep-wake disorders and affective disorders [26–29]. Still, these trials have been small and/or in homogeneous samples with a specified disorder, and have required the study participants to adhere to a protocol at specified times of the day (resting in forced darkness or wearing glasses).

The above represent interesting treatment advances. However, given the prevalence of sleep-wake cycle disturbances in individuals with mental disorders, it is logical to extend trials of the use of such interventions to broader trans-diagnostic populations. Further to enhance generalizability, it would help to avoid giving personal responsibility to individuals who are acutely unwell regarding the timing of their exposure to different

intensities or spectra of light. A pragmatic alternative is to create a therapeutic environment where changes in light exposure are regulated automatically and where programmable lighting conditions form an integral part of a hospital unit. New light-emitting diode (LED) lights can be programmed to emit low levels of blue light which creates a blue-depleted light environment in the hospital in the evening and night. This is an intriguing option as, to date; little consideration has been given to how contemporary technology might be employed to augment any benefits of acute treatment in an inpatient facility. Historically, acute psychiatric admission units have offered asylum and a place of safety, whilst ward routines and structured activities help to reduce arousal, regularize sleep-wake cycle patterns and improve self-esteem, etc. However, the focus is primarily on physical and pharmacological treatments that reduce symptoms and suicidality, enhance social functioning and sufficiently improve the individual's mental state to allow a timely return to outpatient or community care. Less attention has been given to the creation of a state-of-the-art inpatient milieu [30].

Our clinical and research staff has been involved in the planning and design of a newly built psychiatric unit and this process has allowed us to consider how the inpatient environment might be modified to try to enhance recovery from acute illness. The unit comprises of two wards: one ward incorporates state-of-the-art lighting technology whilst the other ward has an identical layout and facilities but has normal lighting conditions. This unit offers a unique opportunity to explore how exposure to different lighting conditions may modify sleep-wake cycles and how any changes in sleep-wake cycle may impact on the clinical and functional outcomes of individuals experiencing an acute episode of a severe mental disorder that requires inpatient care. The findings could influence the future design of hospital units offering care to patients with mental or physical disorders.

Aims

We aim to recruit 400 individuals who give written informed consent to participate in a two-arm pragmatic effectiveness randomized controlled clinical treatment trial (RCT). Based on projected admission rates, we believe that this sample size is at the lower limit of the estimated study population that will be available for inclusion (as we are permitted to continue recruitment for at least six consecutive months).

Eligible individuals will be allocated to a ward with a lighting system that produces an environment with blue-depleted evening light or to a ward with the same layout and facilities but lacking the new lighting technology. The trial will test whether the environment with programmable lighting conditions offers any additional benefits beyond those associated with standard treatment in an acute psychiatric inpatient unit. The main objective is to examine if there is any difference between groups in the mean duration of hospitalization. Also, we will explore whether level of symptoms, functioning, episodes of suicidality or aggressive behavior, medication usage, and self-reported side effects differ between groups and whether a shorter duration of admission is associated with greater stability of sleep-wake cycles and circadian rhythms.

Given that this trial takes place in a unique setting, we will undertake several ancillary investigations to determine the range of benefits or adverse effects for subpopulations of patients (e.g., different diagnostic subgroups, etc.) and examine any potential benefits or drawbacks to the use of this new lighting system, including actigraphic recordings of sleep-wake patterns of nurses and any self-reported effects on well-being that are recorded by professionals who experience working in both wards.

Methods/design

The protocol for the RCT follows the Standard Protocol Items for Randomized Trials (SPIRIT) Statement guidelines [31] and is registered at the ClinicalTrials.gov website with identifier: NCT03788993. The SPIRIT Checklist and other appropriate details are included as an online supplement (Additional file 1).

Trial design, setting, and interventions

This is a single-center, unblinded, two-arm, parallel-group, pragmatic effectiveness RCT of differences in the mean duration of acute psychiatric hospitalization in days for individuals exposed to experimental lighting compared with normal lighting conditions.

The study is located at a newly built acute psychiatric unit at St. Olavs Hospital, Østmarka (Trondheim, Norway) which serves a catchment area of 300,000 people. The unit has 40 patient rooms divided equally between two wards that are built around two atria. Each hospital ward has the same layout and facilities with five rooms (25% of the total) designated as "psychiatric intensive care beds" (targeted at the most severely ill patients and offering a higher staff-to-patient ratio than the rest of the ward). The light intensity (photopic lux) is similar in both wards, but individuals are exposed to a different spectrum of light in each ward (e.g., melanopic lux is kept below 25 in the blue-depleted unit).

During the recruitment phase, patients admitted to the unit are randomized 1:1 to the experimental or control (normal) lighting conditions.

1. Experimental condition: a 20-bedded ward with tunable LED lamps. At 1800 h the lighting

undergoes a 30-min transition during which the green and blue LEDs are dimmed to produce blue-depleted amber-colored lighting. At 0650 h a 10-min transition program changes the light color to normal indoor lighting (3000 K of color temperature) which then continues until 1800 h. The light intensity is dimmed to 20% (of the maximum) from 2300 h to 0650 h

As well as the LED system, blue-blocking window filters are deployed in the evening. All television sets have permanent blue-blocking filters and the outdoor area has external lights that block blue light. Use of electronic media is not restricted (unless access is limited in accordance with an agreed treatment plan), but patients are provided with blue-blocking screens that can be attached to the front of all electronic devices. If a patient leaves the blue-depleted unit after 1830 h they are offered blue-blocking glasses to wear. The amount of blue light in the ward was assessed prior to commencing the RCT. This demonstrated that the light exposure is well-matched to the amount shown in laboratory settings to minimally suppress melatonin [32, 33] and this was tested in an onsite pilot study with healthy adult volunteers (ISRCTN12419665).

2. Control condition: a 20-bedded ward with normal indoor lighting installed. The light intensity is dimmed to 20% (of the maximum) during the night (from 2300 h to 0650 h)

Participants and procedure

The flowchart for the RCT is shown in Fig. 1.

Recruitment

Admissions occurring at any time during each 24-h period and on any day of the week throughout the 6-month recruitment period are considered eligible for study inclusion.

No specific recruitment strategy is employed, but should recruitment fail to reach the required sample size (for any reason) within an allocated 6-month time period, we will recommence recruitment in late 2019, i.e., all recruitment will be undertaken during months with similar daytime light exposure (contact HK for further details).

Eligibility criteria

A frequent barrier to research into acute psychiatric admissions is that, at the time of hospitalization, many individuals lack mental capacity to give informed consent (they may be severely ill, suicidal, lack insight or be hospitalized involuntarily) and/or clinicians and researchers may regard a discussion about research participation inappropriate or unethical. However, as noted in acute medicine research, excluding a large proportion of patients from a RCT purporting to investigate acute admissions can bias the included sample so that it is unrepresentative of the inpatient population and undermines the generalizability and real-world impact of any study findings [34]. For this reason, we did not employ any patient exclusion criteria but, with ethical approval, used a post-randomization, deferred (also known as delayed) consent procedure [34–36]. The RCT eligibility criteria are as follows:

Inclusion criteria All individuals aged ≥ 18 years who are admitted to the acute inpatient unit during the study period are eligible for randomization. Any patients who are re-admitted during the study period are eligible for re-randomization.

Exclusion criteria There are no pre-randomization exclusion criteria, but individuals may be withdrawn from the study immediately post randomization (see "Withdrawal criteria" below).

Randomization

Randomization is based on an allocation concealment procedure.

As soon as the decision to admit the patient is confirmed, the individual is randomly allocated 1:1 to one of the two arms of the RCT using a web-based randomization program with a variable block design. This occurs without any consultation with wards (regarding bed availability, case mix or staffing levels, etc).

The procedure was developed and is managed by the Unit for Clinical Research (at the Department of Medicine and Health, NTNU) and can be implemented at any time of the day or night. Randomization is undertaken by the nurse coordinating the intake process (who participated in pre-trial instruction and training), but they cannot influence the process in any way. When the intake nurse logs in to a web-based program, the randomization program issues an authentication code that is sent to the unit (a telephone back-up system is available should there be any problems with the web-based program).

Withdrawal criteria

As randomization occurs at the point of admission, all exclusions are de facto post randomization. Immediately post randomization, there are two potential reasons for withdrawal from the RCT:

1. Lack of availability of rooms (as allocated at randomization): acute wards operate at high levels of bed occupancy, so sometimes there will be no

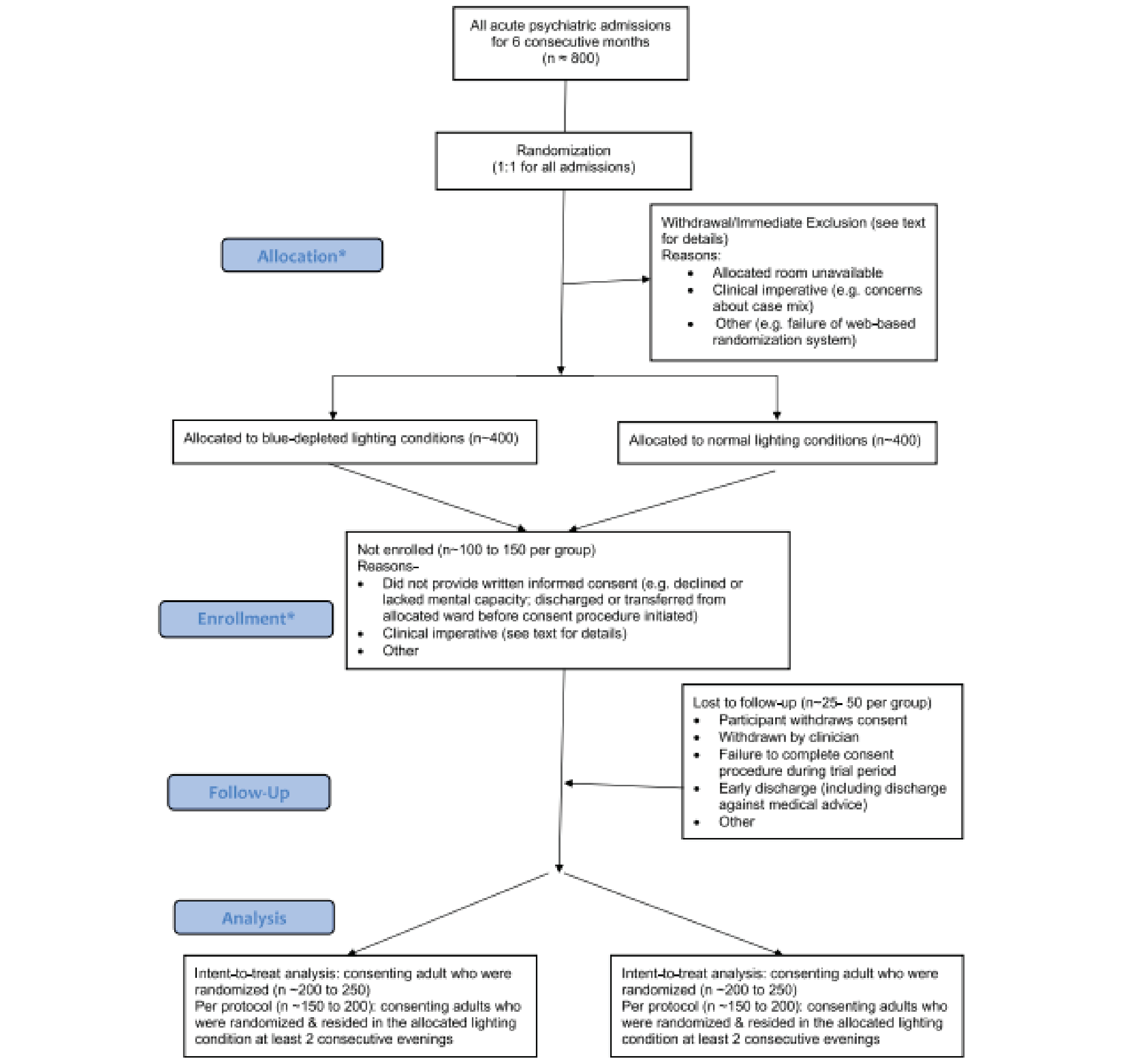

Fig. 1 Study flowchart (*the study employs deferred consent, so allocation precedes enrollment)

rooms available in the ward to which the individual is allocated (i.e., the randomization process cannot be completed)

2. Clinical imperative: sometimes a senior clinician may decide that it is inappropriate to admit an individual to the room to which they are randomized. The most frequent reasons for this decision are clinical concerns about (a) how this admission would affect the case mix within the ward (e.g., it may be inappropriate to co-locate several patients with acute mania) and/or (b) following through with the randomization process may compromise the safety, care or treatment of existing inpatients or of the individual being admitted (e.g., it may not be possible to provide the appropriate staff-to-patient ratio required if all individuals with higher levels of need are co-located)

During the admission withdrawal may occur because:

1. The individual is unwilling to give written informed consent at any time during their admission (when approached according to the deferred consent procedure) or is unable to give informed consent for the duration of the study (i.e., they remain

persistently and severely ill and/or lack mental capacity)

2. The consent procedure was incomplete: an individual may be discharged early or have an unplanned discharge (so they may not be approached about study participation or have only given verbal, but not written consent)
3. A patient will be withdrawn from the study if they are absent for > 24 h from the ward to which they randomized (e.g., they may be transferred to a medical ward for several days; a patient may request or clinicians may instigate transfer to another ward; medical or nursing staff may decide that a patient should be transferred to the other ward participating in the RCT because of clinical, case mix or staffing issues, etc.)
4. An individual can decline to participate at any stage of the study and/or a mental health professional can recommend withdrawal of an inpatient from the RCT if they have any clinical concerns regarding an individuals' participation (e.g., if they believe that a patient has experienced an adverse event associated with exposure to the blue-depleted light)

A record will be kept of all reasons for withdrawal.

Assessments

Descriptions of all measures are provided below, and the timing of study assessments is summarized in Table 1.

A key consideration in the selection of assessment tools was that they were already used routinely or could easily be incorporated into ward procedures, and that clinical staff were familiar with, or had received training in, the use of the instruments. The design and setting of the study mean that it is unfeasible to blind patients, clinicians or investigators. However, we have used electronic hospital records and advanced technology to collect objective data on sleep-wake cycles.

Baseline demographic and clinical information

The intake assessment records detailed information on the following:

a) Age, sex, ethnicity, marital status, living situation, years of education, employment status
b) Current diagnosis or diagnoses (according to the *International Classification of Diseases 10*) [37]. As in our previous research, consensus expert opinion is employed to review all diagnoses at discharge. If the intake and discharge diagnoses differ, the latter

Table 1 Targets and timing of assessments

Assessment	Collected via	Time			
		Intake/first 24 h	Daily	Discharge	Hospital records
Duration of admission in days	Data extracted from electronic records				X
Admission status (voluntary or involuntary)	Data extracted from electronic records	X			
Clinical diagnosis (including comorbid diagnoses)	Mental health professionals[a]	X		X	
Demography and clinical history (including current, past and forensic)	Mental health professionals[a]	X			X
Physical health (including anthropometrics)	Nurse	X			
Clinical Global Impression (CGI)	Mental health professionals[a]	X	X	X	
Clinical Global Impression-Severity (CGI- S)	Mental health professionals[a]	X		X	
Clinical Global Impression-Improvement (iCGI-I)	Mental health professionals[a]	X	X	X	
Risk of suicide	Mental health professionals[a]		X		
Risk of or actual aggressive behavior and associated interventions	Nurse		X		
Monitoring of sleep-wake cycle and daytime activity	Xethru radar sensor		X		
Prescribed medications	Data extracted from electronic records	X			X
Adherence to lighting condition	Nurse		X		
Satisfaction and perceived benefits	Study participant			X	
Side effects	Study participant			X	
Adverse events	Mental health professionals[a]		X		

See text for additional details of rating scales and assessments employed
[a]Primarily psychiatrists or clinical psychologists, but may include trained nurses and/or duty psychiatrist

is employed in analyses (as acute intake diagnoses may be less reliable)

c) Type of admission (voluntary or involuntary), number of psychiatric admissions and total number of inpatient bed-days in the 2 years prior to the index admission and current prescribed medication
d) Details of current presentation including evidence of sleep problems (during the preceding month), level of functioning, alcohol and substance misuse, risk of or actual harm to self or others, current physical health
e) Past psychiatric and forensic history, and history of comorbid medical illnesses

Primary outcome

The primary outcome measure is mean duration of admission in days per individual. Admission is defined as the date and time of commencement of the intake assessment (recorded electronically when the randomization code is generated); discharge is defined as the date and time that the patient left the lighting condition to which they were randomized for > 24 h.

Secondary outcomes

Secondary outcomes are focused on clinical changes over time and treatments or other interventions reported during admission.

a) Clinical assessments

i. Objective assessments:

Sleep-wake cycle: individual sleep and activity patterns will be assessed using de-identified data collected via radar (Xethru sensors) installed in each room. The sensor is a low-powered ultra-wideband radar that allows contact-free assessment of sleep-wake patterns with high accuracy, sensitivity, specificity, and Cohen's kappa compared with polysomnographic (PSG) recordings (mean values 0.93, 0.96, 0.70, and 0.67, respectively) [38].

Employing best available scoring algorithms, raw data from daily recordings will be used to estimate total sleep time (TST), sleep onset latency (SOL), number of nocturnal awakenings, wake after sleep onset (WASO), and final wake time for each participant, along with day-to-day variability in sleep onset, sleep offset, and TST.

ii. Observer assessments

The Clinical Global Impression Scale (CGI) is rated on a 1–7 scale (high scores indicate worse clinical or functional status) and is a well-established outcome measure in trans-diagnostic studies that has moderate to high correlation with disorder specific assessments, both self-reported and clinician administered (e.g., Hamilton Rating Scale for Depression, the Montgomery-Asberg Depression Rating Scale, the Beck Depression Inventory, Hamilton Rating Scale for Anxiety, Positive and Negative Syndrome Scale, Leibowitz Social Anxiety Scale, Brief Psychiatric Rating Scale, Scale for the Assessment of Negative Symptoms) [39, 40].

In this RCT, psychiatrists, psychologists, and nursing staff trained in the use of the CGI, will record a consensus score for each study participant during the daily ward meetings. During the weekends, the psychiatrist on duty and nursing staff will provide the CGI consensus ratings.

The CGI ratings will be based on all available information (nurse observations, clinical assessments, hospital medical records, etc.) and will be used in two ways in this RCT: (a) to monitor day-to-day changes in mental state and functioning, and (b) to record overall change from admission to discharge.

- Clinical Global Impression, Severity subscale (CGI-S) is a Likert scale ranging from 1 to 7 (from "Normal, not at all ill" to "Among the most extremely ill patients") [39]. The CGI-S ratings are benchmarked relative to the total inpatient population and are scored on two occasions only: the morning after admission to the unit and at discharge (based on the preceding 24 h). The intraclass correlation coefficient of CGI-S between independent raters have been shown to be 0.64 for the initial assessment and 0.70 for a second assessment after 14 days [41]
- Clinical Global Impression, Improvement subscale (improved version: iCGI-I) captures change over time with ratings ranging from − 6 (maximum deterioration) to + 6 (ideal improvement) [41]. The iCGI-I is used (a) to monitor day-to-day changes in mental state and functioning, and (b) to record overall change from admission to discharge. The intraclass correlation coefficient of iCGI-I between independent raters has been shown to be 0.74 [41]
- Risk of Harm to Self or Others: suicide risk is assessed daily (rated according to level of risk and/or need of continuous observation) and risk of aggressive behavior is assessed three times per 24 h using the Brøset Violence Checklist (BVC) [42, 43]. The BVC is a six-item scale and the sum score indicates risk of violence (low = 0) and has acceptable interrater reliability (kappa = 0.41 for the sum score) and specificity (Area Under the Curve of Receiver Operating Characteristics = 0.82) [44].

Incidents of aggressive behavior will be systematically recorded using the Staff Observation Aggression Scale-Revised (SOAS-R) [45] and interventions employed will be recorded. The SOAS-R has acceptable interrater reliability (between kappa = 0.61 to kappa = 0.74) [46]

b) Treatments and interventions

i. Medications: daily doses and classes of medications or other treatments or interventions prescribed per individual during admission will be recorded
ii. Change in admission status: if a patient is admitted involuntarily, we will record the time until their status is reclassified as voluntary (as a marker of improved insight and mental capacity). Similarly, for some individuals time to change from voluntary to involuntary status will need to be recorded

Patient-related experiences and other outcomes

For everyone involved with the new unit, a key aspiration is to try to capture information regarding patient experiences of the experimental condition and to evaluate benefits and harms. In this RCT, we will assess acceptability (adherence, perceived satisfaction, and benefits) or harms (side effects and adverse effects) using observer and self-rated assessments.

a) Adherence

For individuals allocated to the blue-depleted conditions, adherence with the intervention is assessed using an item checklist to record any exposure to normal lighting (duration and reasons), whether blue-blocking glasses were worn as appropriate (e.g., when exiting the unit) and whether blue-blocking filters were employed on media devices

b) Satisfaction and benefits

Mean levels of patient satisfaction with an admission are routinely assessed using the standard patient satisfaction questionnaire completed at discharge. The questionnaire was developed by the Norwegian Institute of Public Health, is used throughout the Norwegian Health Care system and consists of 10 items scored on a 5-point Likert scale (1 = low satisfaction). Some items are relevant to examining experiences of the different lighting conditions, side effects (see below) and perceived benefit of the admission (1 = no benefit)

c) Side effects and adverse events

The frequency of any side effects or adverse events experienced by individuals admitted to each lighting condition will be recorded using the eight-item Headache and Eye Strain Scale (HES), which has been shown to be sensitive to exposure to different lighting conditions [47]. This is supplemented by eight items that may reflect side effects of acute psychiatric treatments (e.g., dizziness, gastro-intestinal disturbances, daytime sleepiness, poor sleep quality, and restlessness, etc.). Each symptom is rated on a 4-point scale (ranging from absent to severe).

To capture any putative adverse events experienced during the admission we will record the occurrence of any serious or untoward incidents in each ward (such as non-accidental and accidental deaths, near fatal events, severe violence, etc.). Also, we will note if any patients are transferred out of the blue-depleted light environment because of clinical opinion suggesting that it is having a detrimental effect on the individual.

Other outcomes

Several ancillary studies are planned. For example, ethical approval has been granted to undertake an additional study (running concurrently with the RCT) to examine the experiences of clinical staff who rotate their work schedules between the two wards (ISRCTN21603406), including sub-studies of subjective effects on sleep, cognition, and well-being, and objective actigraphic recordings undertaken during exposure to each lighting condition. Data regarding any side effects may be compared across staff and patients, but other studies will be reported separately.

Sample size

The power calculation and sample size were estimated for the primary outcome measure, namely mean number of days hospitalized per individual exposed to the experimental or control lighting conditions. This outcome was chosen as hospitalization represents a major life event for patients, is the largest contributor to cost of care across psychiatric diagnoses, and it offers a proxy measure of any adjunctive benefits associated with the experimental lighting conditions over and above any gains associated with usual inpatient treatment.

The hospital database showed that there were 1639 acute psychiatric admissions between May 2016 and April 2017, with a mean length of stay of 6.3 days (range 1–158 days; total occupied patient bed-days per annum > 10,000). Assuming that the experimental lighting

conditions lead to a reduction in the mean length of stay from about 6 to 5 days (with a standard deviation of about 3.5 days), then 194 participants in each condition will give an 80% chance at an alpha = 0.05 to detect a difference in the length of stay of 1 day using an intent-to-treat (ITT) analysis (and > 85% power to detect a reduction of 1000 patient bed-days occupied over the course of the year). We have assumed that there will be about 800 admissions to the unit over 6 months, and that post-randomization exclusions (lack of consent, etc.) and sample attrition (e.g., due to early discharge) will amount to about 30% ($n = 240$). Whilst the sample size required is 400 individuals (who give written informed consent), recruitment will continue for the entire 6-month period, and we will continue to randomize all admissions. As such, it is possible that we will be able to include up to 500 individuals.

Data analysis plan

Data management

Data retrieved from electronic hospital records and from assessments will be de-identified and the files will be stored on secure data storage servers.

Statistical analyses

The primary statistical analyses will be performed by an independent statistician who is masked to the lighting conditions experienced by each group.

We will use a linear mixed-model analysis to examine the difference in mean duration of admission in days per individual according to group. As this primary outcome is heavily skewed and not normally distributed, we will use bootstrapping. The main analyses will be based on the ITT population (i.e., all individuals who gave consent and were randomized). Per-protocol (PP) analyses will be limited to participants who are hospitalized for at least two consecutive evenings in the inpatient unit. There will also be some individuals who were not randomized but who give informed consent for the use of some data in additional analyses.

We use a block randomization with random block length (which avoids adding to the burden of the intake process or delaying the admission procedure). As such, we will adjust for a pre-specified subset of demographic and clinical covariates (selected from those listed in the baseline assessments) which are known predictors of duration of a psychiatric admission: these are age, sex, diagnosis, presence of comorbidities, status at admission (involuntary or voluntary), and number of bed-days in the previous 2 years [48, 49]. It should be noted that some patients will be re-admitted and re-randomized during the study period, so we will use a two-level model (with admissions within patient as level 1 and patient as level 2).

For the analysis of the primary outcome, we expect complete data on duration of admission. For analyses of secondary outcomes, we will use multiple imputations to handle missing data as appropriate. We plan to use a mixed model with random intercept and random slope for secondary analyses which have one or more values per admission. Additional analyses will include subgroup (e.g., length of stay according to diagnostic groups; use of medications according to group, etc.) and exploratory analyses (e.g., examining whether improvement in sleep variables mediates duration of admission and risk of harm to self or others; prevalence of self-reported side effects in patients compared to nurses).

Study monitoring

A Data and Safety Monitoring Committee will meet weekly to oversee the study progression, technical issues, and the safety of patients. The committee is comprised of the investigators involved with recruitment, clinical representatives from the unit (mainly psychiatrists and nurses), and statistical advisors; other representatives will be co-opted as required.

The trial sponsor routinely audits of one or more of the ongoing projects each year (selection may be at random or can be via a specified procedure). This monitoring ensures that the trial adheres to the protocol, procedures, and ethical standards and provides independent oversight and quality assurance. No interim analyses are planned.

Patient and public involvement

Patient and public involvement has been a feature of the development of the inpatient service and the proposed research. For example, the leader of the User Group for Mental Health, St. Olavs Hospital, has reviewed the project and offered public support. Also, representatives of the advocacy group have been involved in all the processes related to research at the new unit from the start and have had several meetings with the research group that discussed the study design. Ongoing advice and support have been provided by medical and nursing colleagues working at the unit and others working at St. Olavs Hospital. This included dialog about what assessments could be incorporated into ward routines and the training needs of staff involved in the projects. International experts were consulted regarding the procedure for deferred consent and others offered advice regarding the program for and delivery of the chronotherapeutic interventions.

Dissemination

There is considerable international interest in the lighting technology and the use of this program as an adjunct to standard treatment in an inpatient environment. As

well as publication of findings regarding primary and secondary outcomes, we will publish descriptive articles, present information about the unit and the RCT findings at national and international conferences and will allow site visits by clinicians, researchers and patient advocates who wish to view the unit. The investigators will adhere to international guidelines regarding multi-authorship of manuscripts. No data will be released until all key outputs have been accepted and published in peer-review journals. However, interested parties can contact the senior investigator to discuss data-related issues.

Discussion

There are several significant challenges to undertaking a pragmatic effectiveness RCT in an emergency or acute care setting. The broad range of reasons for acute admission and heterogeneity of psychiatric diagnoses included in the RCT necessitated the selection of a clinically meaningful primary outcome measure and of assessment procedures that minimized any research-related burden placed on patients or clinicians. Whilst an ITT approach is essential to any RCT, there is likely to be considerable "noise" in this primary analysis (e.g., patients may discharge themselves against medical advice; others are admitted in a crisis and then discharged within 24 h). As such, we anticipate that the PP analyses will be important in enhancing understanding of specific patient subgroups that may benefit from exposure to the novel lighting condition, and whether anyone experiences adverse effects. Additional planned analyses should offer important insights into putative benefits or issues that may influence whether other units introduce similar lighting systems in the future. Lastly, we are mindful that the lighting parameters are based on data obtained from healthy controls, and that we may need to further modify the program for exposure to blue-depleted light to optimize benefits for individuals with acute mental disorders.

Trial status

The trial is ongoing and started patient recruitment on 23 October 2018. The study is expected to continue until the end of December 2019. No important protocol amendments are anticipated. However, if any would become necessary, the senior investigator or deputy would be responsible for communicating this to other investigators, the Ethics Committee, grant bodies, etc. Any important changes would be included in the study listing on the website for clinical trials in the Norway so that future participants would be aware of any new eligibility criteria. Protocol versions: the first version of the protocol was approved by the Ethics Committee on 6 June 2018; with two minor amendments submitted on 17 October 2018 and 19 December 2018. This is the second and final version approved on 4 January 2019.

Additional file

Additional file 1: Standard Protocol Items for Randomized Trials (SPIRIT) 2013 Checklist for the protocol. (DOCX 35 kb)

Abbreviations

BVC: Brøset Violence Checklist; CGI: Clinical Global Impression Scale; CGI-S: Clinical Global Impression Scale, Severity subscale; HES: Headache and Eye Strain Scale; iCGI-I: Improved Clinical Global Impression Scale, Improvement subscale; ITT: Intention to treat; LED: Light-emitting diode; NTNU: Norwegian University of Science and Technology; PP: Per protocol; PSG: polysomnography; RCT: Randomized controlled clinical treatment trial; REK: Regional Etisk Komité (Regional Ethical Committee).; SOAS-R: The Staff Observation Aggression Scale-Revised; SOL: Sleep onset latency; SPIRIT: Standard Protocol Items for Randomized Trials; TST: Total sleep time; WASO: Wake after sleep onset

Acknowledgements

We would like to thank the patient user group, Regionalt brukerutvalg Helse Midt-Norge, for its contributions when designing the trial.

Authors' contributions

HK and KL conceived the study. Study design was undertaken by the research team: JS, KL, DV, KK, CLV, PF, SL, AV, GM, TT, and HK. JS produced the first draft of the protocol paper with additional input from and SL and HK. HK tuned the light system. All authors contributed to the drafting of the submitted version of the study protocol and all authors approved the final version of manuscript.

Funding

The project protocol has been reviewed by the Research Council of Norway (project number 275987). St. Olavs Hospital has provided funding for the trial (grant numbers 17/10533–134 and 18/10647–110). Funding has been obtained for two investigators to undertake PhD studies at NTNU (grant numbers 81850077 and 81850104). The funders had no role in designing the study or the decision to submit the protocol for publication.

Availability of data and materials

Not applicable. The manuscript does not contain any data.

Ethics approval and consent to participate

The study protocol was approved by the Regional Ethics Committee of Central Norway on 6 June 2018 (REK 2018/946). The Ethics Committee support the rationale for and use of a deferred consent and agreed that this procedure allowed randomization at the time of acute admission at the discretion of the investigators (and according to recognized guidelines and criteria that were made explicit during the ethical review of the protocol). It was emphasized that informed consent must be sought at the earliest possible time once each individual patient was deemed to have mental capacity by the clinician in charge of the patient [34–36, 50, 51] and that this should be prior to discharge. The consent form specifies that study participants can discontinue participation in the study without stating any reason, and that all study participants are covered by Norwegian System of Patient Injury Compensation system in case of any harm. The consent form is in Norwegian and available upon request. Following the ethics approval, the review by the Ethics Committee and a trial description was made public on the Ethics Committee's webpages, the trial was listed on The Current Research Information System In Norway, Cristin-ID 602154, and patient information about the trial and participation was listed on the website for clinical trials in Norway.

Consent for publication

Not applicable.

Competing interests

The authors declare that they have no competing interests.

Author details

[1]Department of Mental Health, Norwegian University of Science and Technology, Trondheim, Norway. [2]Institute of Neuroscience, Newcastle

University, Newcastle upon Tyne, UK. [3]Division of Mental Health Care, St. Olavs University Hospital, Trondheim, Norway. [4]Department of Research and Development, St. Olavs University Hospital, PO Box 3250, Sluppen, 7006 Trondheim, Norway.

Received: 8 February 2019 Accepted: 16 July 2019
Published online: 01 August 2019

References

1. Grandner MA. Sleep, health, and society. Sleep Med Clin. 2017;12(1):1–22.
2. Foster RG, Wulff K. The rhythm of rest and excess. Nat Rev Neurosci. 2005;6(5):407–14.
3. Brouwer A, van Raalte DH, Diamant M, Rutters F, van Someren EJ, Snoek FJ, et al. Light therapy for better mood and insulin sensitivity in patients with major depression and type 2 diabetes: a randomised, double-blind, parallel-arm trial. BMC Psychiatry. 2015;15:169.
4. Baron KG, Reid KJ. Circadian misalignment and health. Int Rev Psychiatry. 2014;26(2):139–54.
5. Rumble ME, Dickson D, McCall WV, Krystal AD, Case D, Rosenquist PB, et al. The relationship of person-specific eveningness chronotype, greater seasonality, and less rhythmicity to suicidal behavior: a literature review. J Affect Disord. 2018;227:721–30.
6. Langsrud K, Kallestad H, Vaaler A, Almvik R, Palmstierna T, Morken G. Sleep at night and association to aggressive behaviour; patients in a psychiatric intensive care unit. Psychiatry Res. 2018;263:275–9.
7. Langsrud K, Vaaler AE, Kallestad H, Morken G. Sleep patterns as a predictor for length of stay in a psychiatric intensive care unit. Psychiatry Res. 2016;237:252–6.
8. Wulff K, Gatti S, Wettstein JG, Foster RG. Sleep and circadian rhythm disruption in psychiatric and neurodegenerative disease. Nat Rev Neurosci. 2010;11(8):589–99.
9. Kallestad H, Hansen B, Langsrud K, Ruud T, Morken G, Stiles TC, et al. Impact of sleep disturbance on patients in treatment for mental disorders. BMC Psychiatry. 2012;12:179.
10. Goldstein AN, Walker MP. The role of sleep in emotional brain function. Annu Rev Clin Psychol. 2014;10:679–708.
11. Manber R, Edinger JD, Gress JL, San Pedro-Salcedo MG, Kuo TF, Kalista T. Cognitive behavioral therapy for insomnia enhances depression outcome in patients with comorbid major depressive disorder and insomnia. Sleep. 2008;31(4):489–95.
12. Harvey AG, Soehner AM, Kaplan KA, Hein K, Lee J, Kanady J, et al. Treating insomnia improves mood state, sleep, and functioning in bipolar disorder: a pilot randomized controlled trial. J Consult Clin Psychol. 2015;83(3):564–77.
13. Perlis ML, Grandner MA, Brown GK, Basner M, Chakravorty S, Morales KH, et al. Nocturnal wakefulness as a previously unrecognized risk factor for suicide. J Clin Psychiatry. 2016;77(6):e726–33.
14. Sheaves B, Freeman D, Isham L, McInerney J, Nickless A, Yu LM, et al. Stabilising sleep for patients admitted at acute crisis to a psychiatric hospital (OWLS): an assessor-blind pilot randomised controlled trial. Psychol Med. 2018;48(10):1694–704.
15. Creado S, Plante DT. An update on the use of sedative-hypnotic medications in psychiatric disorders. Curr Psychiatry Rep. 2016;18(9):78.
16. Jansson-Frojmark M, Norell-Clarke A. Cognitive behavioural therapy for insomnia in psychiatric disorders. Curr Sleep Med Rep. 2016;2(4):233–40.
17. Roehrs T, Verster JC, Koshorek G, Withrow D, Roth T. How representative are insomnia clinical trials? Sleep Med. 2018;51:118–23.
18. Dallaspezia S, Suzuki M, Benedetti F. Chronobiological therapy for mood disorders. Curr Psychiatry Rep. 2015;17(12):95.
19. Wehr TA, Turner EH, Shimada JM, Lowe CH, Barker C, Leibenluft E. Treatment of rapidly cycling bipolar patient by using extended bed rest and darkness to stabilize the timing and duration of sleep. Biol Psychiatry. 1998;43(11):822–8.
20. Wirz-Justice A, Quinto C, Cajochen C, Werth E, Hock C. A rapid-cycling bipolar patient treated with long nights, bedrest, and light. Biol Psychiatry. 1999;45(8):1075–7.
21. Barbini B, Benedetti F, Colombo C, Dotoli D, Bernasconi A, Cigala-Fulgosi M, et al. Dark therapy for mania: a pilot study. Bipolar Disord. 2005;7(1):98–101.
22. Brainard GC, Hanifin JP, Greeson JM, Byrne B, Glickman G, Gerner E, et al. Action spectrum for melatonin regulation in humans: evidence for a novel circadian photoreceptor. J Neurosci. 2001;21(16):6405–12.
23. Phelps J. Dark therapy for bipolar disorder using amber lenses for blue light blockade. Med Hypotheses. 2008;70(2):224–9.
24. Henriksen TE, Skrede S, Fasmer OB, Hamre B, Gronli J, Lund A. Blocking blue light during mania—markedly increased regularity of sleep and rapid improvement of symptoms: a case report. Bipolar Disord. 2014;16(8):894–8.
25. Henriksen TE, Skrede S, Fasmer OB, Schoeyen H, Leskauskaite I, Bjorke-Bertheussen J, et al. Blue-blocking glasses as additive treatment for mania: a randomized placebo-controlled trial. Bipolar Disord. 2016;18(3):221–32.
26. Burkhart K, Phelps JR. Amber lenses to block blue light and improve sleep: a randomized trial. Chronobiol Int. 2009;26(8):1602–12.
27. Esaki Y, Kitajima T, Ito Y, Koike S, Nakao Y, Tsuchiya A, et al. Wearing blue light-blocking glasses in the evening advances circadian rhythms in the patients with delayed sleep phase disorder: an open-label trial. Chronobiol Int. 2016;33(8):1037–44.
28. Esaki Y, Kitajima T, Takeuchi I, Tsuboi S, Furukawa O, Moriwaki M, et al. Effect of blue-blocking glasses in major depressive disorder with sleep onset insomnia: a randomized, double-blind, placebo-controlled study. Chronobiol Int. 2017;34(6):753–61.
29. Shechter A, Kim EW, St-Onge MP, Westwood AJ. Blocking nocturnal blue light for insomnia: a randomized controlled trial. J Psychiatr Res. 2018;96:196–202.
30. Feifel D. Transforming the psychiatric inpatient unit from short-term pseudo-asylum care to state-of-the-art treatment setting. Psychiatry (Edgmont). 2008;5(9):47–50.
31. Chan AW, Tetzlaff JM, Altman DG, Laupacis A, Gotzsche PC, Krleza-Jeric K, et al. SPIRIT 2013 Statement: defining standard protocol items for clinical trials. Ann Intern Med. 2013;158(3):200–7.
32. Nowozin C, Wahnschaffe A, Rodenbeck A, de Zeeuw J, Hadel S, Kozakov R, et al. Applying melanopic lux to measure biological light effects on melatonin suppression and subjective sleepiness. Curr Alzheimer Res. 2017;14:1042-52.
33. Rahman SA, St Hilaire MA, Lockley SW. The effects of spectral tuning of evening ambient light on melatonin suppression, alertness and sleep. Physiol Behav. 2017;177:221–9.
34. Levine RJ. Deferred consent. Control Clin Trials. 1991;12(4):546–50 discussion 51-2.
35. Abramson NS, Meisel A, Safar P. Deferred consent. A new approach for resuscitation research on comatose patients. JAMA. 1986;255(18):2466–71.
36. Foex BA. The problem of informed consent in emergency medicine research. Emerg Med J. 2001;18(3):198–204.
37. World Health Organization. The ICD-10 Classification of Mental and Behavioural Disorders: diagnostic criteria for research. Geneva: World Health Organization; 1993.
38. Pallesen S, Gronli J, Myhre K, Moen F, Bjorvatn B, Hanssen I, et al. A pilot study of impulse radio ultra wideband radar technology as a new tool for sleep assessment. J Clin Sleep Med. 2018;14(7):1249–54.
39. Busner J, Targum SD. The Clinical Global Impressions Scale: applying a research tool in clinical practice. Psychiatry (Edgmont). 2007;4(7):28–37.
40. Bandelow B, Baldwin DS, Dolberg OT, Andersen HF, Stein DJ. What is the threshold for symptomatic response and remission for major depressive disorder, panic disorder, social anxiety disorder, and generalized anxiety disorder? J Clin Psychiatry. 2006;67(9):1428–34.
41. Kadouri A, Corruble E, Falissard B. The improved Clinical Global Impression Scale (iCGI): development and validation in depression. BMC Psychiatry. 2007;7:7.
42. Linaker OM, Busch-Iversen H. Predictors of imminent violence in psychiatric inpatients. Acta Psychiatr Scand. 1995;92(4):250–4.
43. Woods P, Almvik R. The Brøset Violence Checklist (BVC). Acta Psychiatr Scand Suppl. 2002;412:103–5.
44. Almvik R, Woods P, Rasmussen K. The Brøset Violence Checklist (BVC): sensitivity, specificity and inter-rater reliability. J Interpers Violence. 2000;15(12):1284–96.
45. Palmstierna T, Wistedt B. Staff observation aggression scale, SOAS: presentation and evaluation. Acta Psychiatr Scand. 1987;76(6):657–63.
46. Nijman HL, Palmstierna T, Almvik R, Stolker JJ. Fifteen years of research with the Staff Observation Aggression Scale: a review. Acta Psychiatr Scand. 2005;111(1):12–21.
47. Viola AU, James LM, Schlangen LJ, Dijk DJ. Blue-enriched white light in the workplace improves self-reported alertness, performance and sleep quality. Scand J Work Environ Health. 2008;34(4):297–306.
48. Kahan BC, Rushton H, Morris TP, Daniel RM. A comparison of methods to adjust for continuous covariates in the analysis of randomised trials. BMC Med Res Methodol. 2016;16:42.

49. Vittinghoff E, Glidden DV, Shiboski SC, McCulloch CE. Regression methods in biostatistics linear, logistic, survival, and repeated measures models. 2nd ed. New York: Springer; 2012.
50. Hill AB. Medical ethics and controlled trials. Br Med J. 1963;1(5337):1043–9.
51. Truog RD, Robinson W, Randolph A, Morris A. Is informed consent always necessary for randomized, controlled trials? N Engl J Med. 1999;340(10):804–7.

Publisher's Note

Springer Nature remains neutral with regard to jurisdictional claims in published maps and institutional affiliations.

Ready to submit your research? Choose BMC and benefit from:

- fast, convenient online submission
- thorough peer review by experienced researchers in your field
- rapid publication on acceptance
- support for research data, including large and complex data types
- gold Open Access which fosters wider collaboration and increased citations
- maximum visibility for your research: over 100M website views per year

At BMC, research is always in progress.

Learn more biomedcentral.com/submissions

BMC

REQUEST FOR PARTICIPATION IN A RESEARCH PROJECT

DEVELOPMENT OF A THERAPEUTIC HOSPITAL BUILDING. A RANDOMIZED CONTROLLED TRIAL.

This is a question for you about taking part in a research project to investigate the effects of different types of light for patients admitted to a psychiatric hospital ward. In recent years, new knowledge has been established about the importance of light and darkness for sleep, circadian rhythm and mental health. In Østmarka, the acute ward is therefore divided into two: One half has orange light in the evening, while the other half has standard light in the evening. It's random who gets a room with orange evening light and who gets a room with standard light. In this experiment, we want to examine the treatment results for patients who have been admitted to the acute ward at Østmarka.

WHAT DOES THE PROJECT INVOLVE?

When you were admitted to the ward, a lottery was held as to whether you would get a room in the part of the ward that has orange evening lights or the part that has standard evening lights. Apart from this, nothing is different for you as a patient. No additional tests or samples will be carried out in connection with the study.

In the project, we will collect and register information about you. The information recorded about you will only be used as described in the purpose of the study. We will use information from medical records to assess whether there are advantages to orange evening light instead of standard light. The information from the journal will be analyzed without being linked back to you. It will not be possible to identify you in the results of the study when these are published.

Data from the medical record that will be used are: an assessment of symptoms, function, sleep, restlessness, length of stay, medication you have been given, whether you have been forcibly admitted and whether you have been subjected to coercive measures. We will also use the patient satisfaction questionnaire that you fill in at the end of your stay. When the study is finished, only authorized personnel connected to the project have access to the list of names and can trace back to you.

POSSIBLE ADVANTAGES AND DISADVANTAGES

We are not aware of any side effects from being in a room with orange evening light, but it is possible that you will experience being more tired in the evening.

VOLUNTARY PARTICIPATION AND OPPORTUNITY TO WITHDRAW YOUR CONSENT

Participation in the project is voluntary. If you wish to participate, you sign the declaration of consent on the last page. You can withdraw your consent at any time and without giving any reason. This will have no consequences for your further treatment. If you withdraw from the project, you can request to have collected information deleted, unless the information has already been included in analyzes or used in scientific publications. If you later wish to withdraw or have questions about the project, you can contact project manager and psychological specialist Håvard Kallestad (telephone 72823030 or e-mail address havard.kallestad@ntnu.no).

WHAT HAPPENS TO THE INFORMATION ABOUT YOU?

The information registered about you must only be used as described in the purpose of the project. You have the right to inspect the information that is registered about you and the right to have any errors in the information that is registered corrected. You also have the right to access the security measures when processing the information.

All information will be processed without names and national identification numbers or other directly identifying information. A code links you to your information through a list of names. Only project manager Håvard Kallestad and section supervisor for the acute ward Knut Langsrud have access to this list.

The information about you will be anonymized or deleted no later than five years after the end of the project.

SHARING OF DATA AND TRANSFERS ABROAD

By participating in the project, you also agree that the information collected can be transferred abroad as part of research collaboration and publication. The code that links you to your personally identifiable information will not be disclosed. We will at all times use the collaboration partners that are most appropriate. It is not planned for de-identified information to be sent to countries outside the EU/EEA, but if this becomes relevant, the project manager will ensure that information is handled in a safe manner. An updated list of collaboration partners can be obtained from the project manager.

INSURANCE

St. Olav's Hospital is covered by Norwegian patient compensation.

ECONOMY

The study is financed through research funds from St. Olav's Hospital, NTNU and the Council for Mental Health, the Extrastiftelsen. There are no conflicts of interest.

APPROVAL

The study has been approved by the Regional Committees for Medical and Healthcare Research Ethics (decision 2018/946). The patient organization "Brukergruppa ved St. Olavs Hospital" has approved the study.

According to the new Personal Data Act, the data controller at St. Olav's Hospital, department Østmarka, and project manager Håvard Kallestad have an independent responsibility to ensure that the processing of your data has a legal basis. This project has a legal basis in the EU's personal data protection regulation article.

You have the right to complain about the processing of your information to the Norwegian Data Protection Authority.

CONTACT INFORMATION

If you have any questions about the project, you can contact project manager and psychology specialist Håvard Kallestad at St. Olav's Hospital, department for research and education, telephone 72823030 and e-mail address havard.kallestad@ntnu.no. You can contact the institution's data protection officer if you have questions about the processing of your personal data in the project (Merete Blokkum, mobile 92254635 or e-mail address Merete.Blokkum@stolav.no).

I CONSENT TO PARTICIPATE IN THE PROJECT AND TO MY PERSONAL INFORMATION AND MY BIOLOGICAL MATERIAL BEING USED AS DESCRIBED

Place and date

Participant's signature

Participant's name in printed letters
