# Supplementary material for: Clinical benefits of modifying the evening light environment in an acute psychiatric unit: A single-centre, two-arm, parallel-group, pragmatic effectiveness randomised controlled trial
Source: PLoS Med. 2024 Dec 6;21(12):e1004380. doi: 10.1371/journal.pmed.1004380 (PMC11661622; doi:10.1371/journal.pmed.1004380)
Supplement: S2 File — (PDF) [file pmed.1004380.s002.pdf]

S2 Supplementary information. Statistical Analytic Plan (SAP)

Testing the Effectiveness of an Evening Blue-depleted Light Environment in an Acute Psychiatric Ward

Statistical Analysis Plan

Version 2.0

STATISTICAL ANALYSIS PLAN for BAB-study

Administrative information

Sponsor name	St Olavs Hospital
Sponsor address	St. Olavs Hospital, Department Østmarka<br>Trondheim, Norway, 7040
Regional Ethics Committee of Central Norway approval no.	REK 2018/946
Trial title	Testing the Effectiveness of an Evening Blue-depleted Light Environment in an Acute Psychiatric Ward
Short title (/Trial ID)	BAB-studien
Trial registration number	ClinicalTrials.gov: NCT03788993

SAP and protocol version

SAP version and date	v1.0, July 2021
Published protocol (DOI)	https://doi.org/10.1186/s13063-019-3582-2

SAP revision history

The SAP was developed based on the published protocol.

SAP version	Date changed	Comments
0.1	17.06.21	Stian Lydersen drafted analysis plan for primary outcome
0.2	01.07.21	Melanie Rae Simpson drafted SAP based on published protocol and analysis plan for primary outcome
0.3	27.08.21	Draft revised to include details about planned analyses for secondary and safety outcomes variables.
1.0	29.09.21	Minor revisions after discussions between study team and statisticians, including: clarification of recruitment and randomisation procedure (§2.2) and additional analysis for secondary outcomes (§5.2.4)
2.0	05.12.22	The following amendments were made to the SAP after assessing the dataset and prior to the unblinding of group allocation.<br>• Clarification of calculation of length of stay (§5.1)<br>• Clarification of covariate classification / calculation added as an appendix (§7)<br>• Finalisation of analysis strategy for primary outcome (§5.2) and treatment effect heterogeneity with continuous outcomes (§5.2.2)<br>• Revised analysis strategy for secondary outcomes (particularly BVC / SOAS-R, §5.3)<br>• Specification that medication use will be analysed in subsequent articles (§5.3)<br>• Specification that number of psychiatric diagnoses per individual, as well as diagnostic subtype will be analysed (§5.2 and 7.3).

STATISTICAL ANALYSIS PLAN for BAB-study

Signatures

	Name	Title	Signature	Date
Written by:	Melanie Rae Simpson	Statistician	M. SIMPSON	6th Dec 2022
Reviewed by:	Stian Lydersen	Trial statistician	S Lydersen	6thDec2022
Approved by:	Håvard Kallestad	Principle investigator	[illegible]	6th Dec 2022

Abbreviations

AE	Adverse Event
BVC	Brøset Violence Checklist
CGI	Clinical Global Impression Scale
CGI-S	Clinical Global Impression Scale, Severity subscale
CONSORT	Consolidation Standards of Reporting Trials
CRF	Case Report Form
HES	Headache and Eye Strain Scale
ICD10	International Classification of Disease 10
iCGI-I	Improved Clinical Global Impression Scale, Improvement subscale
IMP	Investigational Medical Product
ITT	Intention-to-treat
LED	Light-emitting diode
NTNU	Norwegian University of Science and Technology
NW	Nocturnal awakenings
PP	Per protocol
PSG	Polysomnography
RCT	Randomised controlled trial
REK	Regional Etisk Komite (Regional Ethical Committee)
SAP	Statistical Analysis Plan
SE%	Sleep efficiency (percentage of time in bed spent asleep)
SOAS-R	The Staff Observation Agression Scale-Revised
SOL	Sleep onset latency
SPIRIT	Standard Protocol Items for Randomised Trials
TST	Total sleep time
WASO	Wake after sleep onset
AE	Adverse Event
BVC	Brøset Violence Checklist
CGI	Clinical Global Impression Scale
CGI-S	Clinical Global Impression Scale, Severity subscale
CRF	Case Report Form
HES	Headache and Eye Strain Scale
iCGI-I	Improved Clinical Global Impression Scale, Improvement subscale
IMP	Investigational Medical Product
ITT	Intention-to-treat
LED	Light-emitting diode
NTNU	Norwegian University of Science and Technology
PP	Per protocol
PSG	Polysomnography
RCT	Randomised controlled trial
REK	Regional Etisk Komite (Regional Ethical Committee)
SAP	Statistical Analysis Plan
SOAS-R	The Staff Observation Agression Scale-Revised
SOL	Sleep onset latency
SPIRIT	Standard Protocol Items for Randomised Trials
TST	Total sleep time
WASO	Wake after sleep onset

Table of contents

Administrative information ii

SAP and protocol version ii

SAP revision history ii

Signatures iii

Abbreviations iv

Table of contents 5

1. Introduction 7

1.1 Purpose and scope of the statistical analysis plan 7

1.2 Background and rationale for the trial 7

1.3 Objectives 7

1.3.1 Primary objective 7

1.3.2 Secondary and exploratory objectives 7

2. Study design 8

2.1 Study design 8

2.2 Randomization and treatment assignment 8

2.3 Determination of sample size 8

2.4 Framework 8

2.5 Statistical interim analyses 8

2.6 Timing of final analysis 8

2.7 Data sources and timing of endpoint assessments 8

3. Statistical principles 9

3.1 Confidence intervals and p-values 9

3.2 Adherence, protocol deviations and protocol violations 9

3.2.1 Adherence 9

3.2.2 Withdrawn from study 9

3.2.3 Protocol deviations 9

3.3 Analysis populations 10

3.3.1 Intention-to-treat population 10

3.3.2 Per-Protocol Analysis Set 10

3.3.3 Subgroup definitions 10

3.4 Allocation concealment and blinding 10

3.4.1 Allocation concealment 10

3.4.2 Blinded statistician 10

4. Presentation of study population 10

4.1 Screening data, eligibility, recruitment and withdrawal 10

4.2 Baseline patient characteristics 10

5. Analysis 11
5.1 Definition of primary outcome 11
5.1.1 Time of admission 11
5.1.2 Time of discharge 11
5.1.3 Length of stay 11
5.2 Analysis of primary outcome 11
5.2.1 Missing data 12
5.2.2 Subgroup analyses and treatment effect heterogeneity 12
5.2.3 Additional analyses 12
5.3 Analysis of secondary outcomes 13
5.3.1 Analysis of continuous secondary outcomes 14
5.3.2 Analysis of binary secondary outcomes 15
5.3.3 Missing data 15
5.3.4 Additional analyses 15
5.4 Analysis of safety outcomes 15
5.5 Analysis of exploratory outcomes 16
6. Reference 16
7. Appendix A – Clarifications of covariates 17
7.1 Diagnoses 17
7.2 Comorbid substance or personality disorder 17
7.3 Involuntary or voluntary admission 17
7.4 Number of admissions and bed-days in the past 2 years 18

STATISTICAL ANALYSIS PLAN for BAB-study

1. Introduction

This Statistical Analysis Plan (SAP) should be read in conjunction with the published trial protocol [1] (Available at: https://doi.org/10.1186/s13063-019-3582-2). The information available here provides a more detailed description of the "Statistical analysis" section.

1.1 Purpose and scope of the statistical analysis plan

This document details the proposed analysis of the main paper(s) reporting results from the trial Testing the Effectiveness of an Evening Blue-depleted Light Environment in the Acute Psychiatric Ward. These main paper(s) will follow the strategy set out here. Any deviations from the analyses outlined in this SAP will be described and justified in the final report of the trial, including the inclusion of any analyses suggested by journal editors and referees. Modifications will be carefully considered and, as far as possible, will follow the broad principles set out here.

First and foremost, this SAP describes the analysis of the primary and secondary outcomes. Subsequent exploratory analyses are also expected to follow the broad principles of this SAP but are not described in detail here. Additionally, there is a parallel study being conducted investigating the experience of staff employed on the wards and the analyses planned for this study are not covered here.

The details presented here shall not prohibit accepted practices, such as data transformation prior to analysis. When possible, such data management and modelling decisions will be undertaken prior to revealing the treatment allocation.

The final analysis strategy will be available on request when the principal papers are submitted.

1.2 Background and rationale for the trial

There is increasing recognition of the need to stabilize sleep-wake cycles in individuals with major mental disorders. As such, clinicians and researchers advocate for the use of interventions targeted at sleep and circadian dysrhythmias as an adjunct to the standard treatments offered for acute illness episodes of a broad range of diagnoses. This project explored the benefits of admitting individuals with major mental disorders to an acute psychiatric inpatient unit where changes in light exposure are integrated into the therapeutic environment of a new psychiatric unit.

1.3 Objectives

1.3.1 Primary objective

The trial will test whether the environment with programmable lighting conditions offers any additional benefits beyond those associated with standard treatment in an acute psychiatric inpatient unit. Specifically, participants will be allocated to a ward with a lighting system that produces an environment with blue-depleted evening light or to a ward with the same layout and facilities but lacking the new lighting technology. The main objective is to examine if there is any difference between groups in the mean duration of hospitalization.

1.3.2 Secondary and exploratory objectives

The secondary objectives of this trial are to explore whether the blue-depleted evening light conditions influences are particularly beneficial in certain diagnostic subgroups, as well as the effect on the level of symptoms, functioning, episodes of suicidality or aggressive behaviour, medication usage, and self-reported side effects differ between groups and whether a shorter duration of admission is associated with greater stability of sleep-wake cycles and circadian rhythms.

2. Study design

2.1 Study design

This is a single-centre, unblinded, two-arm, parallel-group, pragmatic effectiveness randomised controlled trial (RCT).

2.2 Randomization and treatment assignment

A detailed description of the randomisation procedure can be found in the published protocol. Briefly, there will be a 1:1 block randomisation with varying block sizes, using the webCRF program (developed and managed at the Clinical Research Unit Central Norway). Patients who may need admission to the acute psychiatric hospital unit will initially be assessed by other health professionals (general practitioners, out-of-hours emergency services, and other somatic and psychiatric hospital departments). If hospitalisation is deemed necessary, patients will be directed to the acute admissions facility. For the purposes of the trial, patients will be randomised prior to arrival at the unit (i.e. prior to any psychiatric assessment at the acute unit). Clinical considerations may mitigate against following the random allocation procedure, in which cases some participants may be withdrawn immediately from the trial (see section §3.2.2).

2.3 Determination of sample size

The sample size calculation is described in detail in the published protocol[1].

2.4 Framework

This trial is designed to establish the superiority of blue-depleted evening lighting environment compared to normal lighting on the duration of admission to acute psychiatric wards:

- The primary null hypothesis is that the duration of admission does not differ between the lighting environment group
- The primary alternative hypothesis is that the duration of admission differs based on the lighting environment.

There is only one primary outcome in this trial. The other efficacy analyses will be regarded as secondary or exploratory.

2.5 Statistical interim analyses

No interim analyses were planned or performed.

2.6 Timing of final analysis

Data was collected until 14 days after the last participant was randomised. Participants who were still admitted at this time were considered as discharged with their length of stay ending on this date.

2.7 Data sources and timing of endpoint assessments

The primary outcome is duration of admission which is assessed upon discharge. Other secondary and exploratory outcomes are assessed either daily or upon discharge (See Table 1 in the published protocol[1]). Data will be obtained from two main sources: Electronic patient records and Xethru radar sensors as described in the published protocol.

The Xethru radar sensors will be used to obtain data on each patient's sleep-wake cycle. As described in the published protocol, developed algorithms will be used to estimate the total sleep time (TST), sleep onset latency (SOL), number of nocturnal awakenings (NW), wake after sleep onset (WASO), and sleep efficiency (SE%)[2].

3. Statistical principles

3.1 Confidence intervals and p-values

All efficacy and safety estimates will be presented with two-sided 95 % confidence intervals. Accompanying two-sided p-values will be calculated and compared to a 5 % significance level. No adjustments for multiplicity will be made since there is only one primary null hypothesis in this trial. This is in line with "Guideline on multiplicity issues in clinical trials EMA/CHMP/44762/2017".

3.2 Adherence, protocol deviations and protocol violations

Randomised individuals may withdraw or be withdrawn from the allocated treatment group for various reasons as outlined in the published protocol, or they may not be adherent to the light conditions. The published protocol did not distinguish between withdrawals from the intention-to-treat and per protocol analyses, which are clarified in the following sections.

3.2.1 Adherence

For individuals allocated to the blue-depleted light conditions, adherence with the intervention is assessed using an item checklist to record any exposure to normal lighting (duration and reasons), whether blue-blocking glasses were worn as appropriate (e.g., when exiting the unit) and whether blue-blocking filters were employed on media devices.

3.2.2 Withdrawn from study

Randomisation occurs at the point of admission which means that all withdrawals are post-randomisation. Participants withdrawn from the study for the following reasons will be removed from further analyses:

Immediately post randomisation:

- Lack of availability of rooms for the allocated light-condition
- Clinical imperative: A senior clinician decides that admission to the allocated room is inappropriate, e.g. due to case mix within the ward, staff-to-patient ratios

Any time during admission:

- Unwilling to give written informed consent at any time during admission
- Unable to give informed consent for the duration of the stay
- Consent procedure was incomplete
- Declines participation at any stage during the study

3.2.3 Protocol deviations

During the admission withdrawal may occur because:

- The participant was absent for >24 hours from the ward to which they were randomised, for example due to transfer to another hospital for medical/somatic reasons. Any participant who is absent from the ward voluntarily is considered to have been discharged and if they were to return, they would be re-randomised and recorded as another admission. These participants will be included in the intention-to-treat analyses and the duration of admission will be until the date of discharge.
- Clinical concerns regarding an individuals' participation

These scenarios will be considered protocol deviations, and the participants will be removed from any per-protocol analyses (see also §3.3.2).

3.3 Analysis populations

3.3.1 Intention-to-treat population

The intention-to-treat (ITT) population will include all participants who were randomised and provided informed written consent and excluding those withdrawn for reasons outlined in §3.2.2.

3.3.2 Per-Protocol Analysis Set

The per protocol analysis set will include all participants in the ITT population who were hospitalised for at least two consecutive evenings, and who had no protocol deviations (§3.2.3).

3.3.3 Subgroup definitions

A number of baseline characteristics have been identified *a priori* as both predictive of the length of stay and possible sources of treatment effect heterogeneity. These include the variables specified in §5.1

3.4 Allocation concealment and blinding

3.4.1 Allocation concealment

As described, all admitted patients will be randomised prior to arrival at the unit.

3.4.2 Blinded statistician

The analyses will be performed by the trial statistician who will be masked to the lighting conditions experienced by each group for the primary analysis and as far as practically possible for subsequent analyses. This document details the analyses planned *a priori* along with the anticipated assumption checks of the chosen analysis models. These model assumption checks and decisions about the final model will be performed prior to unblinding of the statistician and, if necessary, detailed in an amendment to the SAP that will be published with together with the trial results.

4. Presentation of study population

4.1 Screening data, eligibility, recruitment and withdrawal

All participants admitted during the trial period will be randomised. A Consolidated Standards of Reporting Trials (CONSORT) flow diagram will be used to summarise the number of participants as shown in the published protocol. Reasons for withdrawal post-randomisation will be summarised per treatment arm.

4.2 Baseline patient characteristics

As described in the protocol, the following participant demographics and baseline characteristics to be summarised include:

- Demographics: Age, sex, ethnicity, marital status, living situation, years of education, employment status
- Current diagnosis or diagnoses, according to International Classification of Disease 10 (ICD 10) at discharge
- Type of admission (voluntary or involuntary)
- Previous admission history: number of admissions, total number of inpatients bed-days in the 2 years prior
- Details of current presentation based on health records: sleep problems during previous month, level of functioning, alcohol and substance misuse, risk of or actual harm to self or others, current physical health
- Past psychiatric and forensic history, and history of comorbid illnesses

These demographics and baseline characteristics will be tabulated for each treatment arm and overall using descriptive statistics:

- Continuous variables: N, mean and standard deviation, and median and 25th and 75th percentiles as appropriate
- Categorical variables: counts and percentages for categorical variables.

No formal statistical comparisons of baseline characteristics will be conducted.

5. Analysis

5.1 Definition of primary outcome

5.1.1 Time of admission

The time of admission is considered the time and date which the patient arrived at the ward and was assessed. For some individuals, this time was not recorded and the time of randomisation was taken as the time of admission.

5.1.2 Time of discharge

The time of discharge is registered at midday of the day of discharge. For most participants this represents approximately the time they would have left the ward.

5.1.3 Length of stay

The length of stay was calculated as the date and time of discharge minus the date and time of admission, scaled to represent time in days.

5.2 Analysis of primary outcome

The primary outcome is duration of admission in days in the ITT population. Some patients will be re-admitted and re-randomized during the study period. As such, we initially planned to use a two-level linear mixed-model with duration of stay as dependent variable, with admissions within patient as level 1, and patient as level 2. The primary covariate is group (blue-depleted evening lighting versus normal lighting condition). In an analysis prior to unblinding we found that a two-level model gave an estimated random effect variance approximately equal to zero (and a corresponding p=1.00) with an inestimable confidence interval. Hence, we chose linear regression without multi levels for the final analysis.

As initially planned, we will adjust the analyses for a set of pre-specified baseline variables which can be strong predictors of the outcome, as generally recommended by Kahan et al[3] and Vittinghoff et al[4].

These covariates are:

- Age (continuous variable)
- Sex (binary variable)
- Diagnosis (categorized as psychotic episodes/disorders, manic episode, severe depressive episode, or none of these)
- Comorbidities
  - Alcohol/substance use disorders (binary variable)
  - Borderline or other personality disorders (binary variable)
- Involuntary versus voluntary status at admission (binary variable)
- Number of admissions in the previous 2 years (continuous variable)
- Number bed-days in the previous 2 years (continuous variable)

See Appendix A for clarifications of how these covariates were calculated and coded. An additional subgroup analysis will be conducted considering the number of psychiatric diagnoses per individual (see §5.2.2).

As this primary outcome is heavily skewed and not normally distributed, we use bootstrapping with the bias-corrected and accelerated (BC_a) method and B=5000 bootstrap samples. The computational time for these analyses was acceptable and we did not need to consider a lower number of bootstrap samples, such as B=1000, as described in version 1.0 of SAP.

5.2.1 Missing data

The primary outcome is based on hospital records of date of admission and date of discharge and will have no missing data. Similarly, the baseline variables included in the adjusted model should not have any missing data, perhaps with the exception of the number of admission and numbers of bed-days in the previous 2 years if the patient has recently moved to the Trondheim region. If this occurs, we anticipate that very few participants will have missing information for these variables. The need to employ missing data strategies, such as multiple imputations, will be considered depending on the proportion of missing information.

5.2.2 Subgroup analyses and treatment effect heterogeneity

To assess for treatment effect heterogeneity, subgroup analyses will be performed based on the covariates listed above (§5.1). Additionally, a subgroup analysis of number of psychiatric diagnoses given during admission will be performed, categorising the number of diagnoses per individual as 0, 1, 2 and 3+ diagnosis. For continuous covariates (age, number of previous admissions and duration of previous admissions), we assessed the appropriateness of including these in the linear regression model with an interaction term for investigating treatment effect heterogeneity prior to unblinding of the data. Since the number of previous admission and the total duration of previous admission during the past two years was very highly skewed, we opted to categorise these variables for the purposes of subgroup analyses.

- **Number of previous admissions:** categorised as none, 1 to 2, or ≥3 previous admissions within past 2 years
- **Total duration of previous admissions:** categorised as no, 1 – 10, or ≥11 days previously admitted within past 2 years

Treatment effect heterogeneity due to age was assessed with and interaction term between treatment allocation and age as a continuous variable.

5.2.3 Additional analyses

The primary analysis and subgroup analyses will also be performed on the per protocol population.

5.3 Analysis of secondary outcomes

Table 1: Overview of secondary outcomes and their planned analyses

Secondary outcomes	Time(s) recorded	Timepoint / timeframe for analysis	Planned analysis	Brief description of recorded data
**Clinical assessments and treatment during admission**				
Clinical Global Impression Scale – Improvement (iCGI-I)				Range: -6 (maximum deterioration) to 6 (ideal improvement)
- During admission (day-to-day change)	Daily	During admission	Linear	
- Change from admission	Discharge	At discharge	Linear	
Clinical Global Impression – Severity sub scale (CGI-S)	Baseline and discharge	At discharge	Linear	Likert scale with range: 1 (Normal, not at all ill) to 7 (Among the most extremely ill patients)
Suicide risk	Daily	During admission	Logistic	One item clinical assessment
Need for continuous observation (due to suicide risk)	Daily	During admission	Logistic	One item clinical assessment
Risk of harm to others (Brøset Violence Checklist)	8-hrly	During admission	Linear	6 item scale assessing 6 observable behaviours scored on binary scale. Risk of violence based on total scores: low = 0; moderate = 1-2, higher >2
Staff Observation Agression Scale-Revised (SOAS-R)	Daily	During admission	Linear	Context and severity of aggressive incidents recorded on scale with range 0 to 100 (0 = not severe, 100 = very severe)
Medication use	Daily	During admission	Logistic/Linear[a]	Daily doses and classes of medication recorded
Change in admission status	Daily	During admission	Logistic[b]	Changes from involuntary to voluntary and *vice versa* will be recorded
Sleep wake cycle parameters	Daily	During admission	Linear	Algorithm estimated variables (from Xethru radar sensors). This variable will be analysed and presented in a subsequent publication.
**Patient-related experiences and other outcomes**				
Adherence (item checklist)[c]	Daily	During admission	Logistic	Duration and reason for exposure to normal lighting; use of blue-blocking glasses on exiting unit and filters on media devices. Analysed last since this leads to unblinding of data.
Satisfaction and benefits	Discharge	At discharge	Linear	11 items scored on 5-point Likert scale (1 = low satisfaction)

[a]Analysis of medication use will be presented in subsequent papers, first considering use of anxiolytic and hypnotic medication in addition to the frequency of extra medication that is taken as required (pro re nata). The dosage, frequency and changes in the use of different medication classes will also be considered in exploratory analyses; [b]Change in admission status will be investigated both as the proportion of involuntarily admitted patients who change to voluntary admission, and the number of days of involuntary admission. [c]Variable completed for blue depleted light group only and therefore analysed first after unblinding.

5.3.1 Analysis of continuous secondary outcomes

For continuous variables measured only at discharge (iCGI-I and measure of satisfaction), between group comparisons will be conducted using linear regression adjusting for the same covariates as the in the primary analysis.

For the daily recording of iCGI-I we were able to implement the planned analysis strategy using a three-level linear mixed-model with respective secondary outcome as the dependent variable, day during admission as level 1, admissions within patient as level 2, and patient as level 3. The covariates will include treatment allocation, the prespecified baseline variables and an interaction term between treatment and day of admission.

For the other continuous secondary outcomes, a mixed linear regression was deemed unfeasible upon review of the dataset, prior to any analysis being conducted and we revised the analysis strategy for these variables as follows:

*5.3.1.1 **Description of revised plan for Clinical Global Impression Severity sub scale (CGI-S)***

With only two measurement points (admission and discharge) it was reasonable to conduct a simple linear regression analysis with follow-up CGI-S score at discharge as the dependent variable and adjusting for CGI-S score at admission.

*5.3.1.2 **Description of revised plan Risk of harm to others (Brøset Violence Checklist)***

This six-item checklist creates a score from zero to six. Events with a score of two or more are considered to be serious events. Few participants were responsible for the majority of recorded events.

Notionally, this checklist was to be completed once per shift throughout the admission, however this was not consistently done. Particularly for longer admissions, there was a tendency for the BCV to be completed less often towards the end of admission. On the other hand, clinical experience would suggest that the scale is often completed for when individuals are violent or agitated. As such, missingness in this variable per shift was considered likely to be missing not at random, yet all serious events are most likely recorded with a BVC ≥ 2.

We therefore decided to count the number of times each participant had a BVC ≥ 2 throughout each admission and as number of times this occurred per 100 days. These two measures were analysed using the same strategy as the primary outcome analysis, with adjustment for the same covariates and bootstrapping estimate the 95 % confidence intervals due to the skewed distribution of this variable. Where the calculated confidence interval extended below the possible limit (zero), the lower confidence interval limit was set to zero.

Due to the highly skewed distribution of the number of serious events on the BVC scale, we also calculated the risk ratio for having any such event during an admission in each group.

*5.3.1.3 **Description of revised plan for Staff Observation Aggression Scale-Revised (SOAS-R)***

Similar to the BVC, few individuals were responsible for the majority of reported events in the SOAS-R. Events with a score of nine or more are considered to be serious events. Staff are only expected to record a value for the SOAS-R score when they observe a patient being aggressive, and multiple staff members may record reports for the same event. We therefore categorised an event as a new event if it was reported at least 30 minutes after any previous event for that patient. The highest score for any event was taken as the SOAS-R score for that event. Any patient who had no recorded events during their admission were assumed to have had no aggressive episodes.

Since few participants were responsible for multiple events, some of which occurred on the same day, it was considered not feasible to apply a mixed linear regression model with day of admission as

level 1. Instead, we calculated the number of serious events (SOAS-R ≥ 8) per patient throughout the admission and scaled to per 100 days. As for BVC there two measures were analysed using the same strategy as the primary outcome analysis, with adjustment for the same covariates and bootstrapping to estimate the confidence intervals due to the skewed distribution of this variable.

As for BVC, due to the highly skewed distribution of the number of serious events on the SOAS-R, we also calculated the risk ratio for having any such event during an admission in each group.

5.3.2 Analysis of binary secondary outcomes

Binary secondary outcomes recorded on a daily basis were planned to be analysed using a three-level mixed logistic regression model with the same levels and covariates as described in the mixed linear models above (§5.2.1). Due to failure to convergence of the model, these variables were simplified into a single binary variable across the entire admission (i.e. binary variables representing suicide risk any time during the admission and need for supervision at any time during admission).

Descriptive analysis revealed that a higher risk of suicide was observed more frequently during the first days of admission, and the first days are expected to be a time when the blue-depleted light environment is unlikely to have had any effect. To address the challenges with model convergence and still allow investigation of the effect of treatment allocation on suicide risk and need to supervision, we used logistic regression analyses with cluster sandwich variance estimators and limited to the analyses to the first 14 days of admission when there were still at least 30 participants per group. Each model included treatment allocation, admission day, and interaction between treatment allocation and admission day, as well as the previously described covariates.

5.3.3 Missing data

We anticipate a high level of missing data for some secondary outcome variables. Under the assumption that they are "missing at random", the estimates produced by the mixed regression analyses should provide unbiased estimates of the effect of the lighting conditions on these secondary outcomes. The use of multiple imputations was considered for secondary outcomes since these could not be analysed with mixed regression modelling as planned, however with less than 10 % of values missing for any given secondary analysis, we opted present the complete case results only.

5.3.4 Additional analyses

Subgroup analyses based on psychiatric diagnostic groups are planned for each of the secondary outcomes and will be considered in subsequent papers. Other exploratory subgroup analyses for on the covariates listed in §5.1 will also be considered, particularly if they are found to be important sources of treatment effect heterogeneity for the primary outcomes. These subgroup analyses may be presented in subsequent manuscripts after publication of the primary trial results.

5.4 Analysis of safety outcomes

As specified in the published protocol, the frequency of any side effects or adverse events will be recorded during the trial period. The eight-item Headache and Eye Strain (HES) Scale will be specifically used at discharge to measure adverse effects due to lighting conditions[5], along with another eight-item scale reflecting side effects of acute psychiatric treatments. The average value of these scores will be compared between groups using a t-test. Other reported adverse events will be summarised as binary variables and analysed using Chi-squared test. This will include an analysis of the proportion of patients which were transferred from their allocated ward after clinical opinion. For any adverse events occurring multiple times per participant per admission we will consider alternative statistical analyses such as Poisson regression.

5.5 Analysis of exploratory outcomes

Planned exploratory analyses to assessed in subsequent papers include:

- Causal mediation analyses to determine if improvement in sleep variables and intra-individual variability in sleep variables mediate the effect of the blue-depleted lighting conditions on duration of admission and risk of harm to self or others
- The dosage, frequency and changes in the use of different medication classes will be considered in exploratory analyses later

6. Reference

1. Scott J, et al. A pragmatic effectiveness randomized controlled trial of the duration of psychiatric hospitalization in a trans-diagnostic sample of patients with acute mental illness admitted to a ward with either blue-depleted evening lighting or normal lighting conditions. *Trials* 2019; **20**(1): 472.
2. Heglum HSA, et al. Distinguishing sleep from wake with a radar sensor A contact-free real-time sleep monitor. *Sleep* 2021; **44**(8): zsab060.
3. Kahan BC, et al. The risks and rewards of covariate adjustment in randomized trials: an assessment of 12 outcomes from 8 studies. *Trials* 2014; **15**: 139.
4. Vittinghoff E, et al. Regression methods in biostatistics linear, logistic, survival, and repeated measures models. 2nd ed. New York: Springer; 2012.
5. Viola AU, et al. Blue-enriched white light in the workplace improves self-reported alertness, performance and sleep quality. *Scand J Work Environ Health* 2008; **34**(4): 297-306.

7. Appendix A – Clarifications of covariates

7.1 Clinical Diagnosis

At admission, participants were broadly classified as having one of the following diagnoses: psychotic episodes/disorders, manic episode, severe depressive episode, or none of these (i.e., other).

At the time of discharge, a multi-disciplinary clinical team held a 'diagnostic meeting' at which they recorded the primary diagnosis and one or more secondary diagnoses for each study participant. The diagnostic classification used in the analyses was based on all diagnoses that were recorded for a participant at the time of and/or during the admission as well as the treatment they received (during their admission.

Diagnosis categorisation	ICD-10 code(s)
Psychotic episode / disorder	All diagnoses in F20 (F20-F29)
Manic episode	F30, F31.1 or F31.2
Severe depression	F32.2, F32.3, F31.4, F31.5, F33.2, or F33.3
None of these	Any other diagnosis (not identified in the other reported categories)

7.2 Comorbid substance or personality disorder

Participants were classified as having concomitant alcohol / substance use disorder, or personality disorder. Originally, we intended to make a single variable which categorised participants as either having alcohol/substance use disorder or borderline/other personality disorders, or neither of these diagnoses. Due to there being some overlap between participants with both diagnoses, we instead created two binary variables each indicating the presence or absence of these disorders separately. This decision was made after reviewing the baseline characteristic data, and prior to any analyses.

Comorbidity categorisation	ICD-10 code(s)
Alcohol / substance use disorder	All diagnoses in chapter F10 (F10-F19)
Borderline and other personality disorders	All diagnoses in chapter F60 (F60-F69)

7.3 Number of psychiatric diagnoses

The number of psychiatric diagnoses per individual was included in additional subgroup analysis for the primary outcome, length of stay. Based on the diagnostic categories assigned at discharge, the number of chapter F diagnosis was counted, and participants were categorised as having zero, one, two, or three or more diagnoses.

7.4 Involuntary or voluntary admission

Under the Act relating to the application and implementation of mental health care ["Psykisk helsevernloven"], Norwegian legislation classifies admissions to psychiatric hospitals as voluntary (under §2.1 of the act) or involuntary (under §3.2 or §3.3). The admission status is determined on second day of admission by an independent specialist in psychiatry (i.e. independent form admitting doctor).

Patients whose admission status was recorded in the dataset as pertaining to §4.10 and §5.2 were also considered to be admitted involuntarily as these sections are related to involuntary transfer from another facility and involuntary treatment after judicial sentencing, respectively.

7.5 Number of admissions and bed-days in the past 2 years

For each admission, the number of previous admissions to psychiatric units and total number of bed-days in the two years prior to the index admission. Some participants experienced periods where they were continuously admitted in psychiatric care facility, but may have been transferred between facilities. Each admission to a new facility was counted as an admission.

If the participant had any previous admission where they were admitted more than two years prior to the index admission, but were discharged within two years, that admission was also counted as an admission during the previous two years and the total number of bed-days within that period were included.
