# Supplementary material for: Clinical benefits of modifying the evening light environment in an acute psychiatric unit: A single-centre, two-arm, parallel-group, pragmatic effectiveness randomised controlled trial
Source: PLoS Med. 2024 Dec 6;21(12):e1004380. doi: 10.1371/journal.pmed.1004380 (PMC11661622; doi:10.1371/journal.pmed.1004380)
Supplement: S3 File — Section A: Trial registration. Section B: Daylight in recruitment period. Section C: Reporting of the trial. Section D: Study period, Section E: Clinical imperative: withdrawing a participant from the RCT. Section F: Number needed To Treat (NNT). Section G: Study Monitoring. Section H: Additional analysis: extended follow-up of length of stay. (PDF) [file pmed.1004380.s003.pdf]

S4 Supplementary information, section A-H

A. Trial registration

Registrations to the Regional Committee for Medical and Health Research Ethics in Central Norway are published in a searchable database. The pre-registration items in the WHO Trial Data Set (v 1.3.1) are included in this registration. Submission contents are publicly accessible at no cost, it is open to prospective registrants, managed by a not-for-profit organization, have a mechanism to ensure the validity, scientific and ethical soundness of the registration data.

There were negotiations with hospital managers and nursing staff regarding in house training needs and any logistical issues (e.g. staffing levels in the unit, timing recruitment to try to ensuring study entry could be undertaken during selected months of the year with similar daylight hours) that would influence the formal start date of the trial. We also undertook a pilot study with healthy volunteers to test if the lighting systems produced the excepted effects on sleep and the circadian system (to check lighting programs). Although the protocol was available before the enrolment of the first study participant (October 23rd 2018), the researchers were occupied with some of the tasks noted, and so completing the clinicaltrials.org website submission was delayed slightly. Following submission, the website then requested revisions to clarify some procedures (following review by clinicaltrials.gov reviewers). Thus, final registration of the protocol on the clinicaltrials.org website was approved on December 28th 2018.

B. Daylight in recruitment period

Trondheim, Norway, is located at a latitude of 63.4 degrees north and there are large changes in daylight throughout the year. Because patients are allowed to go outside in the atrium without personnel or to go for a walk with personnel, we limited inclusion to a time of year when sunset occurred before 1900h in order to mitigate the risk of direct sunlight exposure at night when exiting the hospital. This also ensured that all recruitment and assessments took place during Standard Time. Moreover, in the morning in the summer half-year, morning bright light could enter eight east facing rooms in the intervention ward, possibly affecting treatment outcomes for these patients.

C. Reporting of the trial

As planned, the first submission arising from the RCT describes the key clinical outcomes. Other planned publications and ancillary studies evaluate objective assessments of sleep-wake cycles (using radar technology), adherence to lighting conditions, changes in treatments recorded during inpatient admissions.

D. Study period

As noted in the protocol, we aimed to recruit a minimum of 400 individuals who give written informed consent to participate in a two-arm pragmatic effectiveness randomized controlled clinical treatment trial (RCT), with the expectation that we would actually include about 200-250 individuals per group. This recruitment target takes into account 'failure to complete randomization' (i.e. the patient is randomized to a specified lighting condition, but is not admitted to the bed in the ward with the allocated lighting condition; this may occur for a number of reasons that were listed in the original protocol) and the delayed consent procedure.

The recruitment period was about 6 months (Oct 23th 2018 until April 12th 2019), but we set a limit on randomization to March 31st and for obtaining delayed consent of April 12th. This time frame was selected as it corresponds to the winter period in this region of Norway. After March 31st, daylight saving time changes and also we experience very different environmental light exposure (long days, short nights, etc). This is relevant as it means that any extension to the recruitment period needs to be delayed until the environmental light exposure matches that experienced by individuals who entered the RCT at the start of recruitment. As such, we agreed (in advance of starting the RCT) with the ethics committee and trial steering group, that should we fall short of the target sample size after six months or recruitment, then the recruitment process would be suspended and would be re-opened in October 2019 (when the environmental light exposure would be the same as during the original winter recruitment period).

Although our projected randomization rate was as expected (and we had randomized >600 individuals by month 4-5 of the study), two issues had arisen with completion of randomization. The first was totally unpredictable, namely that a regional electricity power failure meant that we could not properly randomize admissions for a 72-hour weekend period (during with >15 admissions occurred). After discussion, it was agreed that the manual system of randomization that was introduced over that weekend could not be regarded as equivalent to the automated system, so these cases were immediately excluded from the RCT sample. Second, the bed occupancy rate in January and February 2019 had been running at about 100%, meaning that many randomizations could not be completed (i.e. the patient was randomized but could not then be admitted to the lighting condition to which they were allocated).

On March 8th 2019, we undertook modelling of the projected number of randomizations that would be completed (i.e. admitted to the lighting condition allocated at randomization), had given written informed consent by April 12th 2019, and the projected admission rates. We believed that this sample size was at the lower limit of the estimated study population (as we are permitted to continue recruitment for at least six consecutive months). We modelled the best- and worst-case scenario. The best-case scenario suggested we would achieve the target sample by April 12th (>400), but the worst-

case scenario suggested we may only have randomized, completed correct allocation and obtained the delayed consent in about 350-360 cases.

Given the above, and the pre-trial agreement that recruitment could be suspended and re-opened in October 2019, this is course of action we chose. As we do not break the codes for randomization (completed as allocated or failed) or match these to which individuals have given consent (via the deferred procedure), we cannot know the exact number of trial participants we need to recruit in October. However, the original protocol stated we would recruit 400-500 participants. With this in mind, we will re-open randomization for six weeks, starting October 2019, and close the consent procedure by the end of November 2019.

E. Clinical imperative: withdrawing a participant from the RCT

Examples of casemix exclusion could include: history of violence or threatening behaviour (physical or verbal) towards other patients at the same unit as the patient was randomized to; several patients with severe behavioral dysregulation in the same ward that would negatively impact the standard treatment for the patient or compromise patient safety; known drug dealer and patient with drug addiction.

F. Number Needed To Treat (NNT)

In this RCT, we estimated NNTs as the number of patients that need to be exposed to the experimental lighting condition for one of them to benefit compared with the control condition. It is defined as the inverse of the absolute risk reduction (AAR). Hence estimation of the NNT is as follows:

NNT = 1/ARR

Where ARR = CER (Control Event Rate) – EER (Experimental Event Rate).

Where the variables named in the equation are from the table below.

Group/Outcome	Outcome Positive	Outcome Negative	Totals
**Experimental**	a	b	n_1=a+b
**Control**	c	d	n_2=c+d
**Totals**	m_1=a+c	m_2=b+d	N=n_1+n_2

It should be noted that many evidence-based medicine programmes provide online calculators that enable researchers to estimate NNT and 95% CI by simply inputting basic information (rather than

calculating NNT and 95% CI by hand). Traditionally, NNTs and 95% CI are always rounded up to the nearest whole number.

Example

In the RCT, 443 of 476 the participants in the ITT analysis had data from the CGI-S score (229 individuals in the control group and 214 in the experimental group). A CGI-S score of =<3 can be used to indicate a mild illness severity (good outcome) and a score of 2' 4 indicating moderate to severe illness. Moderate to severe illness at discharge was identified in 125 individuals in the control group and 87 individuals in the experimental group. Thus, it appears that the Blue depleted Light Environment reduced the proportion of people with moderate to severe illness from 55 % in the control group to 41 % in the experimental group, the absolute risk reduction is:

ARR = CER – EER = (125/229) – (87/214) = 0.55-0.41 = 0.14 (i.e. 14%)

ARR = 0.14 (95 % CI 0.05 to 0.23)

Therefore, the number needed to treat for improvement is:

NNT = 1/ARR = 1/0.14 = 7.12

NNT= 7 (95 % CI 4 to 22)

G. Study monitoring

There was an electricity power outage across the region meant no computer randomization was possible for 48 hours. Patients admitted during this time were excluded as shown in figure 1. There were two technical issues in the experimental condition: the blue-blocking window screens did not ascend at one occasion and the lighting fixtures gave the wrong frequency during the transition phase in a hallway on one occasion. On both occasions, the problem was resolved quickly and did not affect the study procedures.

H. Additional analysis: extended follow-up of length of stay

This trial recruited participants in two periods (October to March 2019 and October to November 2019). Data collection ceased fourteen (14) days after the conclusion each recruitment period. The rationale behind the decision to cease data collection was to minimise the effect of seasonal variation in daylight. This was particularly relevant for the first recruitment period which ended in April 2019. Five (5) participants who were still admitted at the end of the first recruitment period and three (3) at the end of the second recruitment period were discharged after data collection had ceased. Of these, two were randomised to the blue-depleted light environment and the other six to ward with a standard lighting environment. As described in the SAP, participants who remained admitted at the end of data collection were considered to have a length of stay equal to their length of stay at that time. With few participants in this situation, all of whom already had a long admission period, this was considered a pragmatic solution. However, in the review process of this manuscript we acknowledge that we have underestimated the length of stay for these few participants which is unlikely to have influenced the primary analysis of all participants, but may have influenced subgroup analyses with small number of participants. A supplementary post-hoc sensitivity analysis was completed to identify if this decision had influenced the results after collecting the true length of stay from journal records, particularly consider diagnostic subgroups where the number of participants in the diagnostic subgroups was small (Table S6). These participants had remained admitted between 4 and 96 days after data collection ceased (median 12.5 days; IQR 9.75 to 22.25; mean 24 days).
