# Supplementary material for: Clinical benefits of modifying the evening light environment in an acute psychiatric unit: A single-centre, two-arm, parallel-group, pragmatic effectiveness randomised controlled trial
Source: PLoS Med. 2024 Dec 6;21(12):e1004380. doi: 10.1371/journal.pmed.1004380 (PMC11661622; doi:10.1371/journal.pmed.1004380)
Supplement: S1 CONSORT Checklist — (PDF) [file pmed.1004380.s004.pdf]

S3 Checklist

CONSORT 2010 checklist of information to include when reporting a randomised trial*

Section/Topic	Item No	Checklist item	Reported on page No
**Title and abstract**			
	1a	Identification as a randomised trial in the title	line1 and 2
	1b	Structured summary of trial design, methods, results, and conclusions (for specific guidance see CONSORT for abstracts)	line 22 to 55
**5Introduction**			
Background and objectives	2a	Scientific background and explanation of rationale	Introduction, Paragraph 1-3, line 89 to 151.
	2b	Specific objectives or hypotheses	Introduction, Paragraph 4, Aims, line 153 to 160.
**Methods**			
Trial design	3a	Description of trial design (such as parallel, factorial) including allocation ratio	Methods, Paragraph 1, line 163 to 165. Methods, Consort Flow Chart, line 430.
	3b	Important changes to methods after trial commencement (such as eligibility criteria), with reasons	n/a
Participants	4a	Eligibility criteria for participants	Methods, Eligibility criteria, line 210 to 215.
	4b	Settings and locations where the data were collected	Methods, Study design and participants, line 187 to 202.

Interventions	5	The interventions for each group with sufficient details to allow replication, including how and when they were actually administered	Methods, Experimental and control conditions, line 239 to 275.
Outcomes	6a	Completely defined pre-specified primary and secondary outcome measures, including how and when they were assessed	Methods, Primary outcome, line 306 to 312. Methods, Secondary outcomes, line 314 to 367.
	6b	Any changes to trial outcomes after the trial commenced, with reasons	n/a
Sample size	7a	How sample size was determined	Methods, Sample size calculations, line 374 to 381.
	7b	When applicable, explanation of any interim analyses and stopping guidelines	n/a
Randomisation:			
Sequence generation	8a	Method used to generate the random allocation sequence	Methods, Study design and participants, paragraph 2, line 196 to 202.
	8b	Type of randomisation; details of any restriction (such as blocking and block size)	Methods, Study design and participants, paragraph 2, line 196 to 202.
Allocation concealment mechanism	9	Mechanism used to implement the random allocation sequence (such as sequentially numbered containers), describing any steps taken to conceal the sequence until interventions were assigned	Methods, Study design and participants, paragraph 2, line 196 to 202
Implementation	10	Who generated the random allocation sequence, who enrolled participants, and who assigned participants to interventions	Methods, Study design and participants,

			paragraph 2, line 196 to 202. Methods, Eligibility criteria, Paragraph 4, line 218 to 225.
Blinding	11a	If done, who was blinded after assignment to interventions (for example, participants, care providers, those assessing outcomes) and how	Methods, Statistical analysis, line 384 to 386.
	11b	If relevant, description of the similarity of interventions	Methods, Experimental and control conditions, line 239 to 275.
Statistical methods	12a	Statistical methods used to compare groups for primary and secondary outcomes	Methods, Statistical Analysis, line 383 to to 423. S2 Supporting Information, Statistical Analytic Plan.
	12b	Methods for additional analyses, such as subgroup analyses and adjusted analyses	Methods, Statistical Analysis, line 412 to 423. S2 Supporting Information, Statistical Analysis Plan. S4 Supporting Information, Numbers needed to treat.
**Results**			
Participant flow (a diagram is strongly recommended)	13a	For each group, the numbers of participants who were randomly assigned, received intended treatment, and were analysed for the primary outcome	Figure 2, line 430
	13b	For each group, losses and exclusions after randomisation, together with reasons	Figure 2, line 430

Recruitment	14a	Dates defining the periods of recruitment and follow-up	Methods, Study design and participants, line192 to 196. S2 Supporting Information, Statistical Analytic Plan.
	14b	Why the trial ended or was stopped	n/a
Baseline data	15	A table showing baseline demographic and clinical characteristics for each group	Table 1, line 448. S6 Table
Numbers analysed	16	For each group, number of participants (denominator) included in each analysis and whether the analysis was by original assigned groups	Table 2, line 456
Outcomes and estimation	17a	For each primary and secondary outcome, results for each group, and the estimated effect size and its precision (such as 95% confidence interval)	Table 2, line 456.
	17b	For binary outcomes, presentation of both absolute and relative effect sizes is recommended	n/a
Ancillary analyses	18	Results of any other analyses performed, including subgroup analyses and adjusted analyses, distinguishing pre-specified from exploratory	Results, Clinical Outcomes, line 458 to 508. Table 2, line 456. S5 Table, S6 Table, S7 Table, S8 Table, S9 Table, S10 Table.
Harms	19	All important harms or unintended effects in each group (for specific guidance see CONSORT for harms)	Results, Clinical Outcomes, line 505 to 508. Table 2, line 456. S11 Figure.
**Discussion**			
Limitations	20	Trial limitations, addressing sources of potential bias, imprecision, and, if relevant, multiplicity of analyses	Discussion, Paragraph 2-8, lines 510 to 653

Generalisability	21	Generalisability (external validity, applicability) of the trial findings	Discussion, Paragraph 2-8, lines 510 to 653.
Interpretation	22	Interpretation consistent with results, balancing benefits and harms, and considering other relevant evidence	Discussion, Paragraph 2-8, lines 510 to 653.
**Other information**			
Registration	23	Registration number and name of trial registry	Methods, Ethics statement and registration, lines 171 to 185.
Protocol	24	Where the full trial protocol can be accessed, if available	Methods, Ethics statement and registration, lines 171 to 185. S1 Supporting information, Approved registration and protocol.
Funding	25	Sources of funding and other support (such as supply of drugs), role of funders	The online Editorial Manager Submission System. S1 Supporting information, Approved registration and protocol.
