# Supplementary material for: Clinical benefits of modifying the evening light environment in an acute psychiatric unit: A single-centre, two-arm, parallel-group, pragmatic effectiveness randomised controlled trial
Source: PLoS Med. 2024 Dec 6;21(12):e1004380. doi: 10.1371/journal.pmed.1004380 (PMC11661622; doi:10.1371/journal.pmed.1004380)
Supplement: S1 Table — (PDF) [file pmed.1004380.s005.pdf]

S5 Table. Baseline medication assessment

Description of how medications used by participants in the study are categorized

**Category name**	**Included medications**<br>**(trade names are converted)**
Antipsychotics	Amisulpride<br>Aripiprazole<br>Chlorprothixene<br>Clozapine<br>Quetiapine<br>Levomepromazine<br>Olanzapine<br>Paliperidone<br>Risperidone<br>Zuclopenthixol
Antidepressants	Amitriptyline<br>Bupropion<br>Citalopram<br>Escitalopram<br>Phenelzine<br>Fluoxetine<br>Mianserin<br>Mirtazapine<br>Sertraline<br>Venlafaxine
Lithium	Lithium
Antiepileptics	Carbamazepine<br>Lamotrigine<br>Levetiracetam<br>Pregabalin<br>Valproate
Benzodiazepines / Z-hypnotics	Alprazolam<br>Diazepam<br>Clonazepam<br>Lorazepam<br>Nitrazepam<br>Oxazepam<br>Zopiclone
Antihistamines	Alimemazine<br>Hydroksyzine
Other	Buprenorphine/Naloxone<br>Disulfiram<br>Clonidine<br>Lisdexamfetamine<br>Melatonin<br>Methylphenidate
