# Supplementary material for: Clinical benefits of modifying the evening light environment in an acute psychiatric unit: A single-centre, two-arm, parallel-group, pragmatic effectiveness randomised controlled trial
Source: PLoS Med. 2024 Dec 6;21(12):e1004380. doi: 10.1371/journal.pmed.1004380 (PMC11661622; doi:10.1371/journal.pmed.1004380)
Supplement: S2 Table — (PDF) [file pmed.1004380.s006.pdf]

S6 Table. Baseline characteristics

Variable		Blue-depleted evening light environment (n = 232)	Standard light environment (n = 244)	Total (n = 476)
Civil status, n (%)				
	Married	53 (22.8)	56 (23.0)	109 (22.9)
	Partner	116 (50.0)	124 (50.8)	240 (50.4)
	Single	26 (11.2)	16 (6.6)	42 (8.8)
	Divorced/separated	1 (0.4)	3 (1.2)	4 (0.8)
	Unknown or not reported	36 (15.5)	45 (18.4)	81 (17.0)
Ethnicity, n (%)				
	European	189 (81.5)	180 (73.8)	369 (77.5)
	African	3 (1.3)	3 (1.2)	6 (1.3)
	Asian	5 (2.2)	13 (5.3)	18 (3.8)
	Other	2 (0.9)	6 (2.5)	8 (1.7)
	Unknown or not reported	33 (14.2)	42 (17.2)	75 (15.8)
Living situation, n (%)				
	Own home	135 (58.2)	132 (54.1)	267 (56.1)
	Partial support	33 (14.2)	42 (17.2)	75 (15.8)
	No fixed abode	17 (7.3)	15 (6.1)	32 (6.7)
	Unknown or not reported	47 (20.3)	55 (22.5)	102 (21.4)
Employment status, n (%)				
	Not employed	53 (22.8)	53 (21.7)	106 (22.3)
	Part time (<= 50 %)	57 (24.6)	57 (23.4)	114 (23.9)
	Part-time (>50 %)	5 (2.2)	9 (3.7)	14 (2.9)
	Full time	39 (16.8)	52 (21.3)	91 (19.1)
	Unknown or not reported	78 (33.6)	73 (29.9)	151 (31.7)
Education, n (%)				
	Primary school	44 (19.0)	49 (20.1)	93 (19.5)
	High school	107 (46.1)	109 (44.7)	216 (45.4)
	Higher education	19 (8.2)	23 (9.4)	42 (8.8)
	Unknown or not reported	62 (26.7)	63 (25.8)	125 (26.3)
Psychiatric diagnosis, n (%)				
	Psychotic episode / disorder	36 (15.5)	51 (20.9)	87 (18.3)
	Mania episode	14 (6.0)	17 (7.0)	31 (6.5)
	Severe depressive episode	15 (6.5)	13 (5.3)	28 (5.9)
	Other	167 (72.0)	163 (66.8)	330 (69.3)
Duration of current illness, n (%)				
	0 - 14 days	59 (25.4)	59 (24.2)	118 (24.8)
	14 - 30 days	30 (12.9)	25 (10.2)	55 (11.6)
	Over 30 days	72 (31.0)	79 (32.4)	151 (31.7)
	Unknown or not reported	71 (30.6)	81 (33.2)	152 (31.9)
No. of admissions in past 2 yrs, mean (SD)		2.6 (5.2)	2.9 (5.0)	2.7 (5.1)
No. of admissions in past 2 yrs, n (%)				
	None	124 (53.4)	130 (53.3)	254 (53.4)
	1 to 2	39 (16.8)	39 (16.0)	78 (16.4)
	3 or more	69 (29.7)	75 (30.7)	144 (30.3)
Total days admitted in past 2 yrs, mean (SD)		34.4 (75.6)	38.5 (87.0)	36.5 (81.6)
No. admission days in past 2 yrs, n (%)				
	None	123 (53.0)	127 (52.0)	250 (52.5)
	1 to 10 days	45 (19.4)	49 (20.1)	94 (19.7)
	11 days or more	64 (27.6)	68 (27.9)	132 (27.7)
Previous suicide attempts, n (%)				
	No attempts	68 (29.3)	75 (30.7)	143 (30.0)

1 attempt	28 (12.1)	26 (10.7)	54 (11.3)
2 or more attempts	46 (19.8)	36 (14.8)	82 (17.2)
Unknown or not reported	90 (38.8)	107 (43.9)	197 (41.4)
Difficulties sleeping, n (%)			
Never/seldom	29 (12.5)	24 (9.8)	53 (11.1)
Occasionally	37 (15.9)	44 (18.0)	81 (17.0)
Three or more times per week	92 (39.7)	87 (35.7)	179 (37.6)
Unknown or not reported	74 (31.9)	89 (36.5)	163 (34.2)
Frequent awakenings, n (%)			
Never/seldom	32 (13.8)	33 (13.5)	65 (13.7)
Occasionally	36 (15.5)	42 (17.2)	78 (16.4)
Three or more times per week	88 (37.9)	78 (32.0)	166 (34.9)
Unknown or not reported	76 (32.8)	91 (37.3)	167 (35.1)
Early wakening, n (%)			
Never/seldom	41 (17.7)	43 (17.6)	84 (17.6)
Occasionally	40 (17.2)	43 (17.6)	83 (17.4)
Three or more times per week	73 (31.5)	64 (26.2)	137 (28.8)
Unknown or not reported	78 (33.6)	94 (38.5)	172 (36.1)
Violence in past year, n (%)			
Yes	15 (6.5)	19 (7.8)	34 (7.1)
No	152 (65.5)	152 (62.3)	304 (63.9)
Unknown or not reported	65 (28.0)	73 (29.9)	138 (29.0)
Previous criminal conviction, n (%)			
Yes	18 (7.8)	18 (7.4)	36 (7.6)
No	141 (60.8)	150 (61.5)	291 (61.1)
Unknown or not reported	73 (31.5)	76 (31.1)	149 (31.3)
Tobacco use (past 6 mo.), n (%)			
Yes	39 (16.8)	32 (13.1)	71 (14.9)
No	82 (35.3)	84 (34.4)	166 (34.9)
Unknown or not reported	111 (47.8)	128 (52.5)	239 (50.2)
Alcohol use (past 6 mo.), n (%)			
Yes	38 (16.4)	29 (11.9)	67 (14.1)
No	101 (43.5)	109 (44.7)	210 (44.1)
Unknown or not reported	93 (40.1)	106 (43.4)	199 (41.8)
Marijuana use (past 6. mo.), n (%)			
Yes	65 (28.0)	55 (22.5)	120 (25.2)
No	28 (12.1)	32 (13.1)	60 (12.6)
Use of psychiatric medication, n (%)			
No medication	153 (65.9)	146 (59.8)	299 (62.8)
Antipsychotics	65 (26.6)	64 (27.6)	129 (27.1)
Antidepressants	28 (11.5)	28 (12.1)	56 (11.8)
Lithium	10 (4.1)	1 (0.4)	11 (2.3)
Antiepileptic medication	19 (7.8)	18 (7.8)	37 (7.8)
Benzodiazepines / Z-hypnotics	31 (12.7)	28 (12.1)	59 (12.4)
Antihistamines	3 (1.2)	4 (1.7)	7 (1.5)
