# Supplementary material for: Clinical benefits of modifying the evening light environment in an acute psychiatric unit: A single-centre, two-arm, parallel-group, pragmatic effectiveness randomised controlled trial
Source: PLoS Med. 2024 Dec 6;21(12):e1004380. doi: 10.1371/journal.pmed.1004380 (PMC11661622; doi:10.1371/journal.pmed.1004380)
Supplement: S3 Table — (PDF) [file pmed.1004380.s007.pdf]

S7 Table. Observed values for primary and secondary outcomes

	Blue-depleted evening light environment				Standard light environment			
	N	Mean (SD)	Median (25th-75th)	Range	N	Mean (SD)	Median (25th-75th)	Range
**Length of stay (days)**	232	7.06 (9.56)	3.71 (1.75 to 6.94)	0.25 to 49.22	244	6.72 (8.09)	3.68 (1.52 to 8.84)	0.07 to 47.15
**CGI-I**	215	2.15 (1.47)	2 (1 to 3)	-2 to 6	227	1.83 (1.43)	2 (1 to 3)	-2 to 5
**CGI-S**	214	3.33 (0.87)	3 (3 to 4)	1 to 6	229	3.58 (0.96)	4 (3 to 4)	2 to 6
**Brøset Violence Checklist**								
No. of serious events	232	0.03 (0.24)	0 (0 to 0)	0 to 3	243	0.30 (1.11)	0 (0 to 0)	0 to 9
No. of serious events / 100 days	232	0.29 (2.23)	0.00 (0.00 to 0.00)	0.00 to 26.43	243	3.33 (15.77)	0.00 (0.00 to 0.00)	0.00 to 192.00
**SOAS-R**								
No. of serious events	232	0.18 (1.08)	0 (0 to 0)	0 to 11	244	0.12 (0.50)	0 (0 to 0)	0 to 4
No. of serious events / 100 days	232	1.03 (6.15)	0.00 (0.00 to 0.00)	0.00 to 69.47	243	1.73 (10.60)	0.00 (0.00 to 0.00)	0.00 to 140.49
**Satisfaction**	119	3.75 (0.70)	3.9 (3.4 to 4.3)	1.0 to 4.9	121	3.65 (0.68)	3.7 (3.3 to 4.1)	1.6 to 4.8
**Headache & eye scale**	106	1.99 (0.62)	1.9 (1.5 to 2.4)	1.0 to 3.9	109	1.94 (0.62)	1.9 (1.5 to 2.3)	1.0 to 3.9
**Other side effects**[b]	109	2.00 (0.50)	2.0 (1.6 to 2.3)	1.0 to 3.2	114	2.11 (0.55)	2.1 (1.8 to 2.4)	1.0 to 4.0
**Total side effects**[c]	107	1.99 (0.51)	2.0 (1.7 to 2.3)	1.1 to 3.3	110	2.03 (0.53)	2.0 (1.7 to 2.4)	1.0 to 3.9
**Logistic regression**	**N**	**n**	**%**		**N**	**n**	**%**	
**Risk of suicide**	206	88	42.7		206	83	40.3	

**Required supervision due to risk of suicide**	206	38	18.4	206	40	19.4
**Change from involuntary to voluntary status**	40	11	27.5	42	20	47.6

iCGI-I: Clinical Global Impression Scale - Improvment (clinicians' assessment of improvement at time of discharge); CGI-S: Clinical Global Impression - Severity sub-scale (Severity at discharge); BVC: Brøset Violence Checklist (score >= 2 considered "severe"); SOAS-R: Staff Observation Agression Scale-Revised (score >= 9 considered severe). Each regression model is adjusted for diagnostic category, age, sex, whether the current admission was voluntary or involuntary, presence of comorbid substance abuse, presence of comorbid personality disorder, and number of previous admission and number of days admitted during the past 2 years. [a]This logistic regression analysis includes only those who were admitted involuntarily at the review on day 2; [b]Other side effects included dry eyes, mouth or nose; inner disquiet, anxiety, sleepiness during the daytime, poor nighttime sleep quality, too much nighttime sleep, tiredness during the day, restlessness during the day, dizziness, sweating, diarrhea, changed or poor appetite, constipation, nausea or unsettled stomach; [c]Total side effects is an average of all items in both Headache and eye scale and the other side effects.
