# Supplementary material for: Clinical benefits of modifying the evening light environment in an acute psychiatric unit: A single-centre, two-arm, parallel-group, pragmatic effectiveness randomised controlled trial
Source: PLoS Med. 2024 Dec 6;21(12):e1004380. doi: 10.1371/journal.pmed.1004380 (PMC11661622; doi:10.1371/journal.pmed.1004380)
Supplement: S4 Table — (PDF) [file pmed.1004380.s008.pdf]

S8 Table. Subgroup analyses of duration of hospitalization

	N	Blue-depleted evening light environment Mean (95 % CI)	Standard light environment Mean (95 % CI)	Mean difference (95 % CI)	p-value	p-value interact.
**All participants**	**476**	**7.1 (6.1 to 8.1)**	**6.7 (5.8 to 7.5)**	**0.4 (-0.9 to 1.8)**	**0.523**	**n/a**
**Diagnosis**						
Psychotic episode	87	7.1 (3.5 to 10.7)	7.6 (5.6 to 9.6)	-0.5 (-4.5 to 3.5)	0.818	0.239
Mania episode	31	15.2 (8.7 to 21.6)	13.3 (8.1 to 18.4)	1.9 (-6.3 to 10.1)	0.649	
Severe depressive episode	28	23.5 (16.9 to 30.2)	14.9 (9.5 to 20.3)	8.6 (0.1 to 17.1)	0.048	
Other	330	4.9 (3.9 to 5.9)	5.1 (4.1 to 6.1)	-0.2 (-1.5 to 1.1)	0.799	
**Sex**						
Female	283	7.7 (6.4 to 9.0)	6.3 (5.3 to 7.4)	1.4 (-0.2 to 2.9)	0.087	0.114
Male	193	6.3 (4.8 to 7.9)	7.2 (5.5 to 9.0)	-0.9 (-3.3 to 1.5)	0.460	
**Personality disorder**						
No	403	7.0 (5.9 to 8.1)	6.8 (5.7 to 7.8)	0.2 (-1.3 to 1.8)	0.777	0.367
Yes	73	7.7 (5.4 to 10.1)	6.1 (4.8 to 7.6)	1.6 (-0.8 to 4.1)	0.199	
**Substance use disorder**						
No	356	7.2 (6.1 to 8.2)	6.9 (5.9 to 8.0)	0.2 (-1.3 to 1.8)	0.782	0.579
Yes	120	6.9 (4.6 to 9.2)	5.8 (4.1 to 7.6)	1.1 (-1.7 to 3.9)	0.436	
**Admission status**						
Voluntary	394	6.3 (5.1 to 7.4)	5.3 (4.5 to 6.2)	1.0 (-0.4 to 2.3)	0.170	0.204
Involuntary	82	11.1 (8.2 to 14.0)	13.1 (9.9 to 16.4)	-2.1 (-6.5 to 2.4)	0.364	

**Number of admissions in past 2 years**						
None	250	6.9 (4.1 to 9.7)	6.5 (5.2 to 7.8)	0.6 (-1.2 to 2.3)	0.528	
1 or 2	94	7.1 (5.6 to 8.5)	6.7 (5.1 to 8.4)	1.2 (-1.8 to 4.2)	0.444	0.781
3 or more	132	7.9 (5.3 to 10.5)	6.9 (4.1 to 9.7)	-0.3 (-3.0 to 2.6)	0.855	
**Number of days admitted in past 2 years**						
None	254	7.5 (5.2 to 9.7)	6.1 (4.8 to 7.3)	0.6 (-1.1 to 2.3)	0.463	
Between 1 and 10	78	6.7 (5.2 to 8.2)	7.2 (4.9 to 9.5)	-0.5 (-4.0 to 2.9)	0.762	0.831
11 or more	144	6.7 (4.1 to 9.2)	7.5 (5.2 to 9.8)	0.5 (-2.0 to 3.1)	0.677	
**Number of psychiatric diagnoses**						
None	33	4.9 (3.7 to 6.2)	6.7 (3.7 to 9.6)	-1.7 (-4.7 to 1.2)	0.253	
One	268	7.2 (5.9 to 8.6)	7.2 (5.9 to 8.6)	-0.1 (-1.9 to 1.8)	0.935	
Two	129	7.6 (5.4 to 9.7)	5.5 (4.2 to 6.7)	2.1 (-0.2 to 4.4)	0.074	0.209
Three or more	45	7.2 (2.9 to 11.4)	6.3 (2.1 to 10.6)	0.8 (-5.2 to 6.9	0.785	
