# Supplementary material for: Clinical benefits of modifying the evening light environment in an acute psychiatric unit: A single-centre, two-arm, parallel-group, pragmatic effectiveness randomised controlled trial
Source: PLoS Med. 2024 Dec 6;21(12):e1004380. doi: 10.1371/journal.pmed.1004380 (PMC11661622; doi:10.1371/journal.pmed.1004380)
Supplement: S5 Table — (PDF) [file pmed.1004380.s009.pdf]

S9 Table. Per protocol analyses of duration of hospitalization (n=349)

	N	Blue depleted evening light environment Mean (95 % CI)	Standard light environment Mean (95 % CI)	Difference (95 % CI)	p-value	p-value interact.
**All participants**	**349**	**9.1 (7.8 to 10.4)**	**8.7 (7.5 to 9.9)**	**0.4 (-1.4 to 2.1)**	**0.689**	**n/a**
**Diagnosis**						
Psychotic episode	74	8.6 (4.5 to 12.6)	8.7 (6.6 to 10.8)	-0.1 (-4.6 to 4.4)	0.956	0.247
Mania episode	30	17.5 (10.9 to 24)	14 (8.8 to 19.2)	3.5 (-4.8 to 11.8)	0.413	
Severe depressive episode	27	23.8 (17.1 to 30.5)	16.4 (10.8 to 22.1)	7.4 (-1.2 to 16)	0.092	
Other	218	6.3 (5.0 to 7.5)	7.1 (5.6 to 8.6)	-0.8 (-2.7 to 1.0)	0.391	
**Sex**						
Female	208	9.6 (8.0 to 11.2)	8.3 (6.9 to 9.7)	1.3 (-0.7 to 3.3)	0.207	0.201
Male	141	8.4 (6.4 to 10.3)	9.4 (7.1 to 11.7)	-1.0 (-4.1 to 2)	0.499	
**Personality disorder**						
No	307	8.8 (7.4 to 10.1)	8.7 (7.4 to 10.1)	0.0 (-1.9 to 2)	0.987	0.212
Yes	42	11.4 (7.8 to 14.9)	8.5 (6.5 to 10.6)	2.8 (-1 to 6.6)	0.143	
**Substance use disorder**						
No	275	9 (7.7 to 10.3)	8.8 (7.5 to 10.2)	0.1 (-1.7 to 2)	0.899	0.612
Yes	74	9.4 (6.1 to 12.7)	8.1 (5.3 to 11.0)	1.3 (-3 to 5.6)	0.550	
**Admission status**						
Voluntary	272	8.2 (6.6 to 9.7)	7.4 (6.3 to 8.6)	0.7 (-1.2 to 2.6)	0.458	0.549
Involuntary	77	12.3 (9.2 to 15.3)	13.1 (9.7 to 16.5)	-0.9 (-5.6 to 3.9)	0.721	
**Number of admissions**						
None	186	9.2 (5.6 to 12.9)	8.5 (6.8 to 10.2)	0.4 (-1.8 to 2.7)	0.714	0.576

1 or 2	67	8.9 (7.1 to 10.7)	8.6 (6.5 to 10.7)	2.1 (-1.9 to 6.2)	0.301	
3 or more	96	10.7 (7.3 to 14.2)	9.2 (5.6 to 12.9)	-0.8 (-4.5 to 2.9)	0.673	
**Number of days admitted**						
None	187	9 (6.3 to 11.6)	8.2 (6.6 to 9.9)	0.5 (-1.8 to 2.7)	0.688	
Between 1 and 10	50	8.7 (6.9 to 10.6)	9.8 (6.3 to 13.3)	-0.4 (-5.4 to 4.6)	0.882	0.947
11 or more	112	9.4 (5.9 to 12.9)	9 (6.3 to 11.6)	0.6 (-2.6 to 3.8)	0.717	
