# Supplementary material for: Clinical benefits of modifying the evening light environment in an acute psychiatric unit: A single-centre, two-arm, parallel-group, pragmatic effectiveness randomised controlled trial
Source: PLoS Med. 2024 Dec 6;21(12):e1004380. doi: 10.1371/journal.pmed.1004380 (PMC11661622; doi:10.1371/journal.pmed.1004380)
Supplement: S6 Table — (PDF) [file pmed.1004380.s010.pdf]

S10 Table. Additional analysis: extended follow-up period for duration of hospitalization (n=476)

| | N | Blue-depleted evening light environment Mean (95 % CI) | Standard light environment Mean (95 % CI) | Mean difference (95 % CI) | p-value | p-value interact. |
|---|---|---|---|---|---|---|
| **All participants** | 476 | 7.5 (6.2 to 8.9) | 7.0 (6.0 to 8.1) | 0.5 (-1.2 to 2.2) | 0.578 | |
| **Diagnosis** | | | | | | |
| Psychotic episode | 87 | 6.5 (2.6 to 10.4) | 6.8 (4.3 to 9.4) | -0.4 (-4.6 to 3.9) | 0.870 | 0.800 |
| Mania episode | 31 | 14.6 (8.0 to 21.1) | 14.5 (8.3 to 20.7) | 0.0 (-8.9 to 9.0) | 0.994 | |
| Severe depressive episode | 28 | 23.7 (17.0 to 30.4) | 18.2 (10.1 to 26.3) | 5.5 (-5.0 to 16.1) | 0.306 | |
| Other | 330 | 5.7 (3.9 to 7.6) | 5.4 (4.2 to 6.7) | 0.3 (-1.6 to 2.2) | 0.744 | |
