# Supplementary material for: Clinical benefits of modifying the evening light environment in an acute psychiatric unit: A single-centre, two-arm, parallel-group, pragmatic effectiveness randomised controlled trial
Source: PLoS Med. 2024 Dec 6;21(12):e1004380. doi: 10.1371/journal.pmed.1004380 (PMC11661622; doi:10.1371/journal.pmed.1004380)
Supplement: S1 Fig — (PDF) [file pmed.1004380.s011.pdf]

S11 Figure. Side effects

The Headache and eye strain (HES) scale is an 8-item scale with symptoms associated with the following symptoms rated from 1 to 4 (severe): irritability, headache, eye strain, eye discomfort, eye fatigue, difficulty focusing, difficulty concentrating and blurred vision. There was no apparent difference in HES score between the two groups (mean difference: -0.04, 95 % CI -0.21 to 0.12, p = 0.600). Similarly, we did not observe any statistically or clinically significant difference in the average score of other potential side effects of psychiatric interventions (mean difference 0.11, 95 % CI -0.03 to 0.25, p = 0.114). These other potential side effects included: dry eyes, mouth or nose; inner disquiet, anxiety, sleepiness during the daytime, poor nighttime sleep quality, too much nighttime sleep, tiredness during the day, restlessness during the day, dizziness, sweating, diarrhea, changed or poor appetite, constipation, nausea or unsettled stomach. There was no clear difference in the score of individual items between groups either (data not shown). No additional side effects were reported in either group.

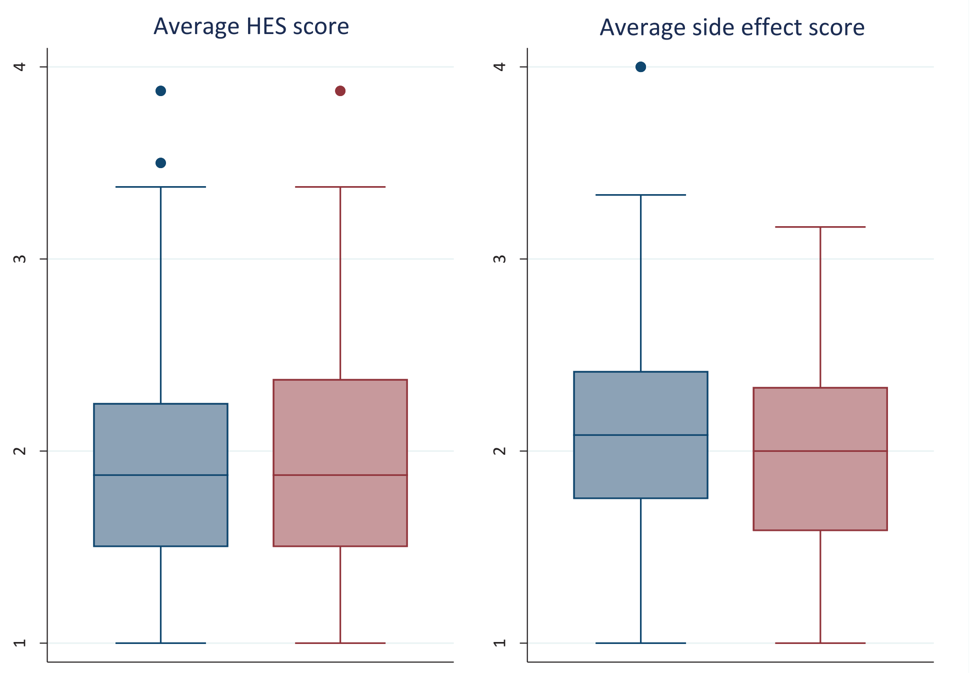

Fig 2S: Bar graphs displaying the distribution of average HES (left) and other side effects (right) scores. Blue bars represent standard light environment and red the evening blue depleted light environment.
